# Effects of parthenolide on amino acid metabolism and oxidative stress in lung adenocarcinoma based on quantitative proteomic analysis, targeted amino acid metabolomics, network pharmacology, and experimental validation

**DOI:** 10.3389/fonc.2025.1642866

**Published:** 2025-09-01

**Authors:** Jiye Liu, Yu Liu, Jiachun Li, Shuang Shen

**Affiliations:** ^1^ Department of Rehabilitation Medicine, Teaching Base of Huludao Central Hospital Affiliated to Jinzhou Medical University, Huludao, Liaoning, China; ^2^ Department of Family Medicine, Shengjing Hospital of China Medical University, Shenyang, Liaoning, China; ^3^ Department of Science and Education, Teaching Base of Huludao Central Hospital Affiliated to Jinzhou Medical University, Huludao, Liaoning, China; ^4^ Department of Pharmacy, Teaching Base of Huludao Central Hospital Affiliated to Jinzhou Medical University, Huludao, Liaoning, China; ^5^ Clinical Laboratory,Teaching Base of Huludao Central Hospital Affiliated to Jinzhou Medical University, Huludao, Liaoning, China

**Keywords:** lung adenocarcinoma, parthenolide, amino acid metabolism, oxidative stress, tumor

## Abstract

**Background:**

Lung adenocarcinoma (LUAD) causes millions of deaths annually worldwide. Parthenolide (PTL), extracted from traditional Chinese herbal medicines, has various biological activities. In this study, we investigated the effects of PTL on amino acid metabolism and oxidative stress in LUAD cells.

**Methods:**

This study identified differential proteins and potential mechanisms of action of PTL in LUAD cells through label-free quantitative proteomics and protein-protein interaction networks. Combined with targeted amino acid metabolomics, we confirmed the results of GO and KEGG analyses. On this basis, the potential targets of PTL in LUAD were identified through network pharmacology, molecular simulation docking, and molecular dynamics simulations. Finally, the effects of PTL on amino acid metabolism and oxidative stress in LUAD were verified using *in vivo* and *in vitro* experiments.

**Results:**

PTL treatment of LUAD cells resulted in significant changes in expression of 157 proteins. GO and KEGG enrichment analyses showed that these proteins were involved in amino acid metabolism and oxidative stress response. Targeted amino acid metabolomics further confirmed that PTL affected amino acid metabolism in LUAD. Network pharmacology, molecular docking, and molecular dynamics simulations identified GCTG as a potential target of PTL in LUAD. Meanwhile, *in vitro* and *in vivo* experimental results indicated that PTL targeting GCTG affected the proliferation, amino acid metabolism, and oxidative stress levels of LUAD cells.

**Conclusion:**

PTL affects proliferation, amino acid metabolism, and oxidative stress levels of LUAD cells via targeting GCTG. Therefore, our study provides new insights into the prevention and treatment of LUAD with PTL, which may lay the foundation for future research directions

## 1 Background

Lung cancer is one of the most common type of cancers worldwide and a leading cause of cancer-related deaths (1). The World Health Organization (WHO) estimates that there are approximately 2 million new cases and 1.76 million deaths each year, and these numbers are constantly increasing (2). Medical experts have deepened their understanding of the biological behavior and pathogenesis of lung cancer over the past few decades and there have been breakthroughs in its treatment (3, 4). However, the 5-year survival rate of lung cancer is still low, and the recurrence rate of the disease and poor quality of life of patients have not been effectively addressed (5). In China, these issues have become even more prominent, becoming a major public health issue that imposes a heavy burden on society and families.

According to the histopathological classification of lung cancer, adenocarcinoma accounts for approximately half of its incidence rate (6, 7). Therefore, diagnosis and treatment of lung adenocarcinoma (LUAD) is an important part of lung cancer research. Although the proliferation rate of LUAD is lower than that of small-cell lung cancer, the occurrence of malignant biological behaviors is relatively late (8, 9). However, owing to the large population of China, early cancer screening is not yet universal. Most patients reach an advanced stage when they visit the hospital, and their 5-year survival rate is not optimistic (10). Currently, surgery, radiotherapy, and chemotherapy are the cornerstones of LUAD treatment; moreover, emerging interventional therapies and immunotherapies in recent years have provided patients with more choices (11, 12). However, the uncertainty of LUAD prognosis and various toxic side effects not only bring more difficulties to the treatment itself, but also seriously affect the quality of life of the patients (13). Therefore, the identification of safer and more effective treatments is urgently warranted in LUAD research.

Traditional Chinese medicine (TCM) has been used in China for thousands of years. Modern medicine has been proven to have significant therapeutic effects on various diseases, including tumors (14). In the treatment of tumors, TCM is mainly used to alleviate adverse reactions after surgery, radiotherapy, and chemotherapy as well as to improve the quality of life of the patients (15, 16). However, each TCM decoction contains many types of herbs, and each herb contains multiple compounds. Therefore, it is difficult to explain the effects of TCM on tumors using specific mechanisms, which greatly hinders the research progress in this field (17). Therefore, there is a critical need to extract effective single components from various herbs and explore their effects on tumors.

Parthenolide (PTL) is a sesquiterpene lactone derived primarily from *Tanacetum parthenium* (18). The nucleophilic properties of the methylene gamma-lactone ring and epoxy group enable them to rapidly interact with biological targets, induce cellular oxidative stress, and exert biological effects (19, 20). PTL is widely used to treat high fever, headache, toothache, rheumatoid arthritis, and other inflammatory diseases (21). In recent years, the antitumor effect of PTL has been observed in many tumors, such as hepatocellular carcinoma and breast cancer, and it does not affect normal cell function.

Several studies have reported the effects of PTL on LUAD. They mostly focus on the action of PTL on the NF-kB pathway and STAT3 pathway, leading to cell apoptosis (22, 23). However, no studies have explored the impact of PTL on LUAD from the perspectives of metabolic reprogramming and oxidative stress. As is well known, the malignant growth of tumors requires continuous acquisition of energy, leading to significant metabolic heterogeneity between tumor tissue and normal tissue. Since the proposal of the Warburg effect, tumor metabolic reprogramming has been the focus of many scientists. Recent research has demonstrated that in addition to sugar metabolism, which provides energy for tumor cell proliferation, amino acid metabolism also plays an irreplaceable role in the occurrence and development of tumors. The occurrence and progression of tumors are often accompanied by an imbalance between the oxidative and antioxidant systems, leading to intracellular DNA damage, protein oxidation, and lipid peroxidation (24). The metabolism of amino acids such as glutamine and serine may cause changes in oxidative stress levels within tumor cells (25, 26). Therefore, we investigated whether PTL affects amino acid metabolism and oxidative stress levels in LUAD cells.

## 2 Methods

### 2.1 Reagents

PTL was purchased from GLPBIO (Montclair, USA) and the human LUAD cell line, H1975, was purchased from the Shanghai Institutes for Biological Sciences, China. The RPMI 1640 medium was purchased from Thermo Fisher Scientific (Waltham, MA, USA). Fetal bovine serum was purchased from Oumashi Biotechnology Co., Ltd. (Shanghai, China). All amino acids and stable isotope-labelled standards were obtained from Sigma-Aldrich (St. Louis, MO, USA). Analytical grade ammonium acetate was obtained from Sigma-Aldrich (St. Louis, MO, USA). Methanol (Optima LC-MS), acetonitrile (Optima LC-MS), and formic acid (Optima LC-MS) were purchased from Thermo Fisher Scientific (FairLawn, NJ, USA). Ultrapure water was purchased from Millipore (Billerica, MA). TCEP (tris (2-carboxyethyl) phosphine) and IAA (iodoacetamide) were purchased from Sigma-Aldrich (St. Louis, MO, USA). Sequence-grade trypsin (sequence grade) was purchased from Promega (Madison, WI, USA). NP-40 (Nonidet P 40) and acetone were purchased from Sangon Biotech (Shanghai, China). The protease inhibitor cocktail was obtained from Kangchen Biotech (Shanghai, China). RNAkey Total RNA Extraction Kit was purchased from Beijing Saiwen Innovation Biotechnology Co., Ltd. The qPCR quantitative analysis and reverse transcription kits were purchased from Novozan Biotechnology(Nanjing,China). The poly (vinylidene fluoride) membrane and ECL reagent were purchased from Sigma (USA). The relevant antibodies were provided by Abclonal (Beijing, China). An amino acid extraction kit was purchased from Solarbio (Beijing, China). The NADPH/NADP detection kit was obtained from Wuhan Saiyu Biotechnology Co., Ltd. The GSH/GSSG detection kit was provided by Nanjing Jiancheng Bioengineering Research Institute.

### 2.2 Cell culture and treatment

H1975 cells were cultured in complete RPMI 1640 medium containing 10% fetal bovine serum, 100 U/mL streptomycin, and 100 U/mL penicillin. H1975 cells were incubated in an environment of 5% CO_2_ and 37°C. According to the appropriate concentration (inhibitory concentration 50 [IC50]) standardized in the initial experiment, PTL was dissolved in 0.05% DMSO, diluted to 10 μM with PBS, added to the treatment group, and an equal amount of complete culture medium or culture medium lacking amino acids was added to the control group.

### 2.3 Label-free quantitative proteomics

The process was divided into nine steps as follows: 1) sample lysis; 2) BCA(Bicinchoninic Acid Assay) quantification; 3) acetone precipitation; 4) protein reconstitution, reduction, alkylation, and enzymatic hydrolysis; 5) removal of SDC(sodium deoxycholate); 6) peptide desalination; 7) Nano HPLC(High Performance Liquid Chromatography) separation; 8) LC-MS/MS(Liquid Chromatography-Tandem Mass Spectrometry); and 9) MaxQuant analysis and LFQ(label-free quantification).

### 2.4 Protein-protein interaction network construction and GO and KEGG analysis

We constructed a PPI network of differentially expressed proteins using the String data platform (https://cn.string-db.org/), and the final graph was implemented using the R package”igraph” (1.2.6). The biological processes, molecular functions, and related signaling pathways of the differentially expressed proteins were analyzed using MetaScape(http://metascape.org/gp/index.html#/main/step1) software. The parameters related to the enrichment analysis were set to a minimum overlap of 3, p-value cutoff of 0.01, and minimum enrichment of 1.5. Using R language - ggplot2 [3.4.4] package was used for GSEA (Gene Set Enrichment Analysis) Analysis.

### 2.5 Standard solution preparation and extraction of metabolites

Stock solutions of the individual amino acids were mixed and prepared in an amino acid-free matrix to obtain a series of amino acid calibrators. Certain concentrations of L-Alanine-d4 and Phenylanine-d2 were mixed as internal standards (IS). The stock solution of all of these and the working solution were stored in -20°C. The samples were added to 450 μL water and 2 mL of acetonitrile/methanol (1:1), which contained mixed internal standards, and vortexed for mixing. Next, the samples were placed in liquid nitrogen and thawed at room temperature. Subsequently, the samples underwent ultrasonic treatment for 4 min, was placed in liquid nitrogen again for 5 min, and thawed at room temperature. The samples were centrifuged at 12,000 rpm for 20 min at 4°C. Finally, the supernatant was injected into a LC-MS/MS system for analysis.

### 2.6 LC-MS method

An ultra-high performance liquid chromatography coupled to tandem mass spectrometry (UHPLC-MS/MS) system (ExionLC™ AD UHPLC-QTRAP 6500+, AB SCIEX Corp., Boston, MA, USA) was used to quantitate amino acids in Novogene Co., Ltd. (Beijing, China). Separation was performed on an ACQUITY UPLC BEH Amide column (2.1×100mm, 1.7μm), which was maintained at 50°C. The mobile phase, consisting of 0.1% formic acid in 5 mM ammonium acetate (solvent A) and 0.1% formic acid in acetonitrile (solvent B), was used at a flow rate of 0.30 mL/min. The solvent gradient was set as follows: initial 80% B, 0.5 min; 80_–_70% B, 2 min; 70_–_45% B, 4 min; 45_–_80% B, 6.01min; 80% B, 9 min.

The mass spectrometer was operated in positive multiple reaction mode (MRM). Parameters were as follows: IonSpray Voltage (5500 V), Curtain Gas (35 psi), Ion Source Temp (550°C), and Ion Source Gas of 1 and 2 (50 and 60 psi).

### 2.7 Construction of pharmacological networks

To explore the relevant targets of the disease, the keyword “lung adenocarcinoma” was searched using OMIM (http://omim.org/), GeneCards (http://www.genecards.org/), and DisGeNET (https://disgenet.com/) databases. Relevant targets of parthenolide were extracted from the SEA search server (https://sea.bkslab.org/), PharmMapper server (https://lilab-ecust.cn/pharmmapper/index.html), and SwissTargetPrediction(http://swisstargetprediction.ch/). The intersection of disease targets, drug targets, and proteomic results was obtained. The intersection of these targets was considered as a potential target for the treatment of LUAD with parthenolide. Subsequently, the standard names of the targets were retrieved from UniProt KB (http://www.uniprot.org/). Relevant targets were obtained from differentially expressed amino acid metabolites using MetScape in Cytoscape 3.8.0. Finally, a compound–target–amino acid network diagram was constructed that included the relationships between parthenolide, related target genes, and amino acids.

### 2.8 Molecular docking

The PDB files of the targets were downloaded from the PDB database (RCSB PDB, http://www.rcsb.org/pdb/). They were preprocessed using Pymol software, and protein hydrogenation and charge calculations were performed using Autodock software and converted into pdbqt files. The SDF file for the compound was downloaded from PubChem (https://pubchem.ncbi.nlm.nih.gov/). Chem3Dpro software was used to calculate its minimum energy, convert it into a mol file, and use autodock to convert it into pdbqt; then Vina molecule docking was used, with the ligand position in the PDB file as the docking interface; the CONF file of each folder was the docking location; Vs_desult was the docking result; The binding energy between components and targets, as well as the number of hydrogen bonds formed, were important criteria for evaluating molecular docking results. The lower the binding energy and the more hydrogen bonds there are, the more stable the binding becomes, and greater the possibility of target molecule interactions. Pymol (open-source) was used to draw 3D and 2D graphs, and Discovery Studio 4.5 Client was used to display docking sites.

### 2.9 Molecular dynamic simulation

A 100 ns MD simulation was performed on the composite using Gromacs 2023. The protein adopted CHARMM 36 force field parameters, and the ligand topology was constructed using GAFF2 force field parameters. The protein-ligand complex was placed in a cubic box under periodic boundary conditions. The box was filled with water molecules using the TIP3P water model. The particle grid Ewald (PME) and Verlet algorithms were used to separate the electrostatic interactions. Subsequently, 100000 steps of the isothermal isostatic ensemble equilibrium and isothermal isobaric ensemble equilibrium were performed using a coupling constant of 0.1 ps and a duration of 100 ps. The van der Waals and Coulomb interactions were calculated using a cut-off value of 1.0 nm. Finally, the system was subjected to molecular dynamics simulations using Gromacs 2023 at a constant temperature (300 K) and pressure (1 bar) for a total duration of 100 ns.

### 2.10 RT-qPCR(Reverse Transcription Quantitative Polymerase Chain Reaction)

Cells were divided into three groups: control, PTL, and PTL + OE-GCTG (also known as GGCT). The concentration of PTL was 10 μM. qPCR experiments were conducted 48 h after cell transfection. Plasmids were prepared using GenePharma (Shanghai, China). qPCR of extracted mRNA was performed according to the manufacturer’s instructions. The primer sequence was as follows: GCTG (upstream): 5’- TCGGCGGCGTTCTTCTGTG-3’; GCTG (downstream):

5’- GCTATCCCTCCATGCCAAGTTTG-3’; GAPDH (upstream):
5’- GGAGTCCACTGGCGTCTTCAC-3’; GAPDH (downstream):
5’- ATTGCTGATGATCTTGAGGCTGTTG-3’.

In addition, RT-qPCR experiments involving human tissues were performed after obtaining informed consent from the patients and were approved by the Ethics Committee of Shengjing Hospital (Ethics Code: 2023PS1232K). For basic experimental analysis. 32 pairs of cancer and adjacent normal tissue samples were collected from patients who underwent the same surgery at Shengjing Hospital between January 2019 and December 2021.

### 2.11 Western blot

Proteins were extracted from the three groups of cells mentioned earlier, quantified, and denatured. Sodium dodecyl sulfate-polyacrylamide gel electrophoresis (SDS-PAGE) was used to separate the same amount of protein, which was then transferred to a polyvinylidene fluoride membrane with a pore diameter of 0.45 μM. Subsequently, the membrane was sealed with a rapid blocking solution and incubated overnight with primary antibody at 4°C. After washing, the membrane was incubated with a secondary antibody at room temperature for 1 h. Finally, ECL reagent was used to visualize the protein bands on a gel imaging system (GE Healthcare). In addition, the grayscale values of each band were analyzed using ImageJ software.

### 2.12 Cell clone formation experiment

In each group, 800 cells/well were inoculated in a 6-well culture plate, which consisted of complete culture medium of 2 ml per well, with three wells in each group. The inoculated cells were shaken well and incubated on a clean bench for 20 minutes, then cultured in the incubator. The medium was changed every 3 days, the cell status observed, the supernatant discarded, and the cells washed once with PBS until the number of cells in most individual clones in the well exceeded 50. Next, 1 mL of 4% paraformaldehyde was added to each well and the cells were fixed at 4°C for 60 min and washed once with PBS. Crystal violet staining solution (1 mL) was added to each well and the cells were stained for 15 min. The cells were then washed several times with PBS, air-dried, photographed, and counted.

### 2.13 Amino acid content determination experiment

Cells were incubated using RPMI 1640 medium without amino acids. Cells were collected into centrifuge tubes and the supernatant was discarded after centrifugation. According to the instructions, 1 mL of the corresponding reagent was added to every 5 million cells, and the cells were sonicated (power 20%, sonication for 3 seconds, interval of 10 seconds, repeated 30 times). Subsequently, the liquid was centrifuged at 10000 rpm and 4°C for 10 minutes, and the supernatant was collected. The enzyme-linked immunosorbent assay (ELISA) reader was preheated for more than 30 min, and the wavelength was adjusted to 570 nm. Amino acid content in the supernatant was measured and calculated according to the manufacturer’s instructions.

### 2.14 NADPH/NADP detection

Eight ml NADPH probe buffer (component B-II) and 2 ml NADPH probe (component B-I) was mixed together. Two μl of storage solution was diluted with PBS to obtain 2 μM working solution. The cells were seeded in a 6-well plate, counted to 1x10^6,^ and treated. The cells were resuspended in 50 μl of component D and repeatedly freeze thawed thrice in liquid nitrogen. A 96-well plate was prepared and 50 μl of PBS solution was added to blank and standard wells 1–6, respectively; 50 μl of NADPH working solution was added to standard well 7. Fifty microliters of the processed sample suspension was added to each experimental well; next, 50 μl of the mix was added to all wells and incubated at room temperature in the dark for 1 h. A ELISA reader was used to detect the OD value at 460 nm.

### 2.15 GSH/GSSG detection

Cells were plated in a 6-well plate and treated before counting. The number of cells in the sample were adjusted to 1x10^6^, and the cells were resuspended using 110 μl of reagent D. Configuration of 50 μM GSH standard and 50 μM GSSG standard was performed. Next, 10 μl PBS was added to the blank well of the 96-well plate, 10 μl GSH standard to the standard well, and 10 μl of the test cell suspension to the experimental well. Then, 100 μl of reagent A and 10 μl of reagent B was added to each well in sequence, and incubated at room temperature for 2 minutes. Then 50 μl of reagent C was added to each well. After 30 s, the OD value at 450 nm was measured using the ELISA reader and the reading was recorded (A1); after 10 minutes, OD value was measured again under the same conditions and the reading was recorded (A2). Result analysis: GSH (μM)=[Measurement well (A2-A1)/standard (A2-A1)] x standard concentration (50 μM). GSSG was determined as described above.

### 2.16 Reactive oxygen species detection

5×10^5^ cells were seeded in a 6-well plate and subjected to the corresponding treatment. Next, 10 μM ROS probe reagent was added to each well (leaving one blank well). After incubation at 37°C for 30 minutes, the treated cells were digested with trypsin and resuspended. After adjusting the parameters, the average fluorescence intensity of the blank well cells was set to 10 and the average fluorescence intensity of the other cells was sequentially detected by adding ROS probes.

### 2.17 Pull-down assay

Biotinylated drug (10 μM) was incubated with streptavidin beads (10 μL) in binding buffer (200 μL total volume) for 1 h at 4°C with rotation.Beads were washed 3× with ice-cold binding buffer to remove unbound drug.Target protein lysate (100 μg) was added to the drug-bound beads and incubated for 2 h at 4°C with rotation.Beads were pelleted using a magnetic rack and washed 3× with binding buffer.

### 2.18 Animal experiments

All experimental procedures involving animals were approved by the Animal Ethics Committee of the Shengjing Hospital (Ethics Code: 2023PS1263K). Twelve female nude mice (BALB/C, 4–5 weeks old, weight 13–15 g) were purchased from Beijing Huafukang Biotechnology Co., Ltd. All nude mice were kept under SPF conditions (22 ± 0.5°C, 50 ± 10% relative humidity, free access to food and water). Nude mice were housed in a SPF-grade animal breeding center for 1 week. Subsequently, 12 nude mice were randomly divided into control, PTL, and PTL+OE-GCTG groups (n=4). Lentivirus preparation was performed by Genepharma. Normal H1975 or OE-GCTG H1975 cells were digested, centrifuged, resuspended in serum-free medium, and counted to a final concentration of 1x10^7^/ml. Cells were randomly inoculated into the posterior lower part of the right forelimb of nude mice using a sterile syringe, with an injection volume of 120 μl. Normal H1975 cells were inoculated in the control and PTL groups, whereas OE-GCTG H1975 cells were inoculated in the PTL + OE-GCTG group. PTL was dissolved in 0.05% DMSO and diluted in PBS. The PTL and PTL+OE-GCTG groups were intraperitoneally injected with PTL [(20 mg/kg, dose selection based on the results of our previous study (unpublished)]; results showed that 20 mg/kg PTL significantly inhibited the volume of LUAD tumors in nude mice. Therefore, this dose was selected for subsequent analyses. The control group was administered an equal volume of DMSO+PBS without any other treatment. The drugs were injected three times a week for four consecutive weeks, and the body weight and tumor volume of the nude mice were measured twice a week. After 4 weeks, the mice were euthanized using the cervical dislocation method after anesthesia. Photographs of the tumor were taken, and relevant experiments were conducted.

### 2.19 Statistical analysis

The extracted proteomic and amino acid metabolomic data were analyzed using univariate statistical analysis, multivariate statistical analysis, hierarchical clustering, and correlation analysis. Univariate statistical analysis was conducted using the Student’s t-test and multiple variation analysis. When the p-value was less than 0.05, the distribution of the measured data showed a decrease of less than 0.67 times or an increase of more than 1.5 times, which was considered a significant difference between cells treated with estradiol. The multivariate statistical analysis included unsupervised principal component analysis (PCA), supervised partial least squares discriminant analysis (PLSDA), and orthogonal partial least squares discriminant analysis (OPLSDA). The discrimination of the tested substances was determined by the variable importance (VIP>1) and p-value (p<0.05) of the projection (VIP) parameter. Gel Pro Analyzer and Image J software were used for grayscale analysis, while SPSS 22.0 and GraphPad Prism7.0 software were used for statistical analysis. Quantitative data were presented in the form of mean ± standard deviation. After testing whether the data in each group followed a normal distribution, a t-test or one-way ANOVA was used to detect intergroup differences. P<0.05 indicated statistical significance.

## 3 Results

### 3.1 Quantitative proteomics analysis shows significant differences in protein expression

**Figure 1:**
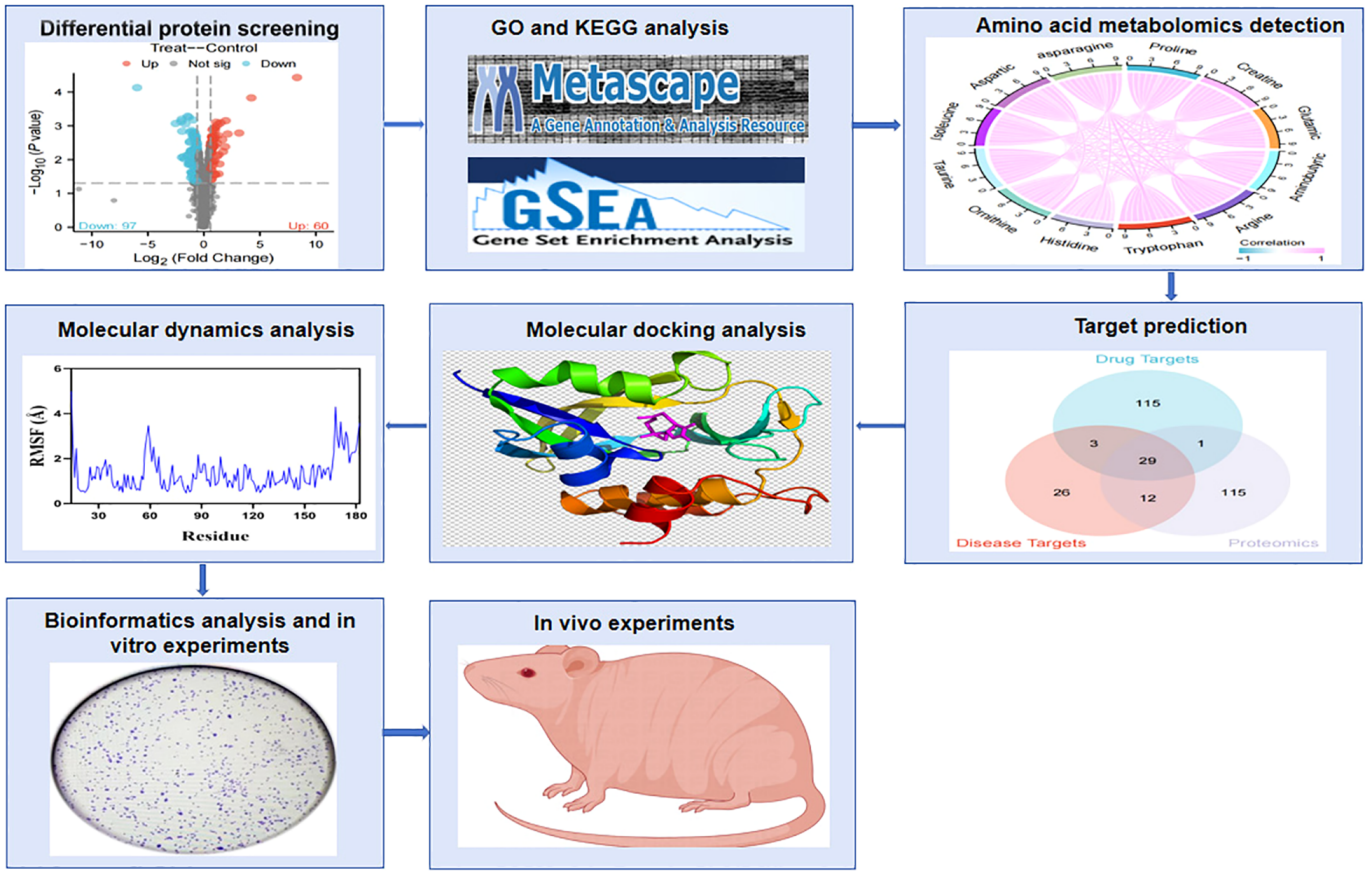
Flowchart of the experimental process. The effects of PTL on amino acid metabolism and oxidative stress in lung adenocarcinoma were revealed through quantitative proteomics, targeted amino acid metabolomics, network pharmacology, and experimental verification. PTL, Parthenolide.

**Figure 2:**
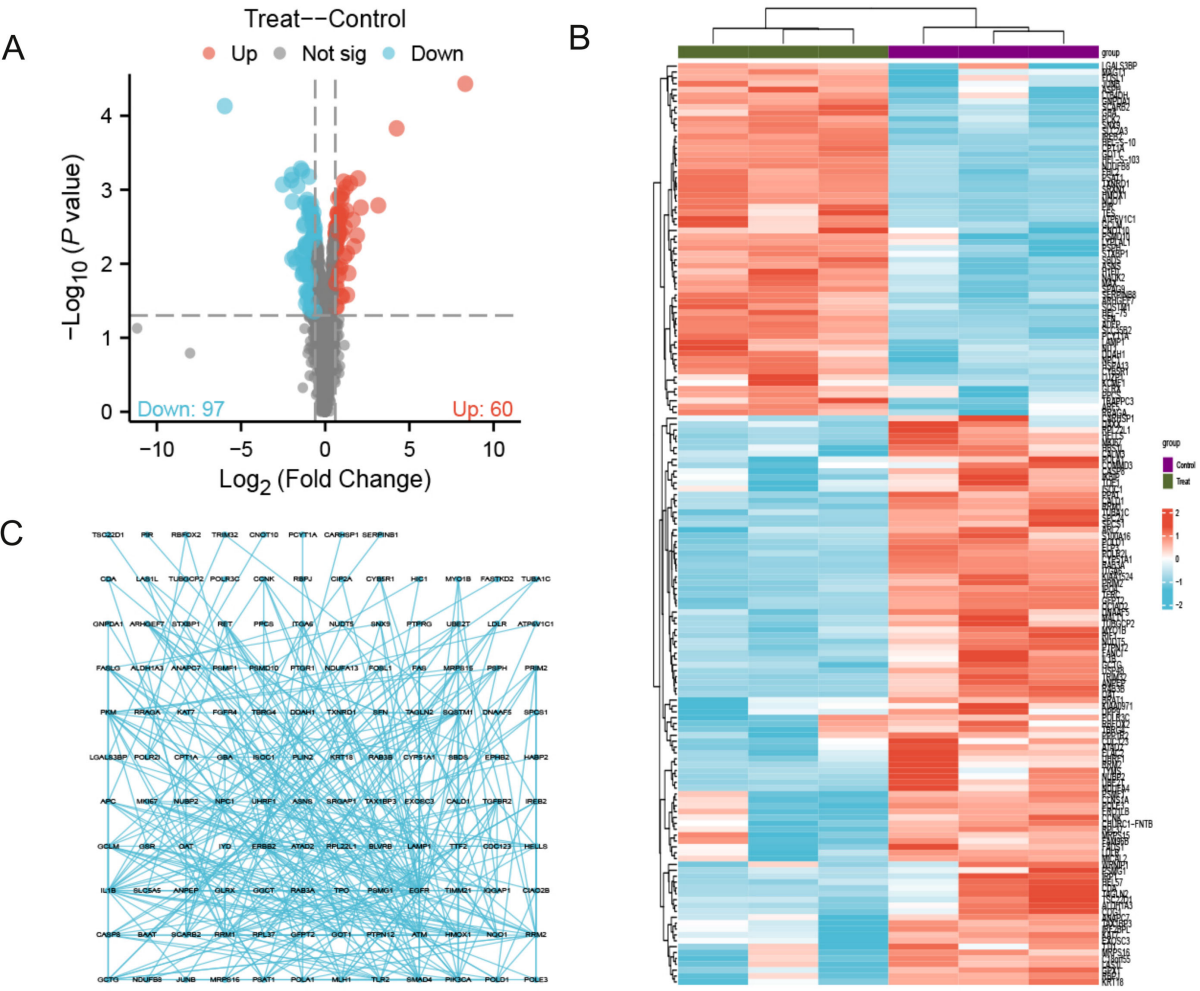
Quantitative proteomics reveals 157 significantly differentially expressed proteins. **(A)** The volcano map contains 60 up-regulated proteins and 97 down regulated proteins; **(B)** Cluster analysis shows that differentially expressed proteins with similar expression patterns form a cluster; **(C)** Protein-protein interaction (PPI) network diagram reveals differential protein interactions. The concentration of PTL was 10 μM. The experiment included three biological replicates.

As shown in **Figure 1**, the flowchart illustrates the study process. A total of 157 differentially expressed proteins were screened by mass spectrometry-based parallel reaction monitoring (PRM), including 60 upregulated and 97 downregulated proteins (**Figure 2A**, **Supplementary Tables S1, S2**). As shown in **Figure 2B**, the significantly altered proteins formed a cluster, indicating that they have similar expression patterns and may be closely related to metabolic processes. As shown in **Figure 2C**, the PPI network revealed significant differential protein interactions in this group, which may indicate that it plays a special role in certain molecular processes or cellular functions.

### 3.2 GO and KEGG analysis results indicate that differential proteins converge in amino acid metabolism processes and anti-oxidative stress pathways

**Figure 3:**
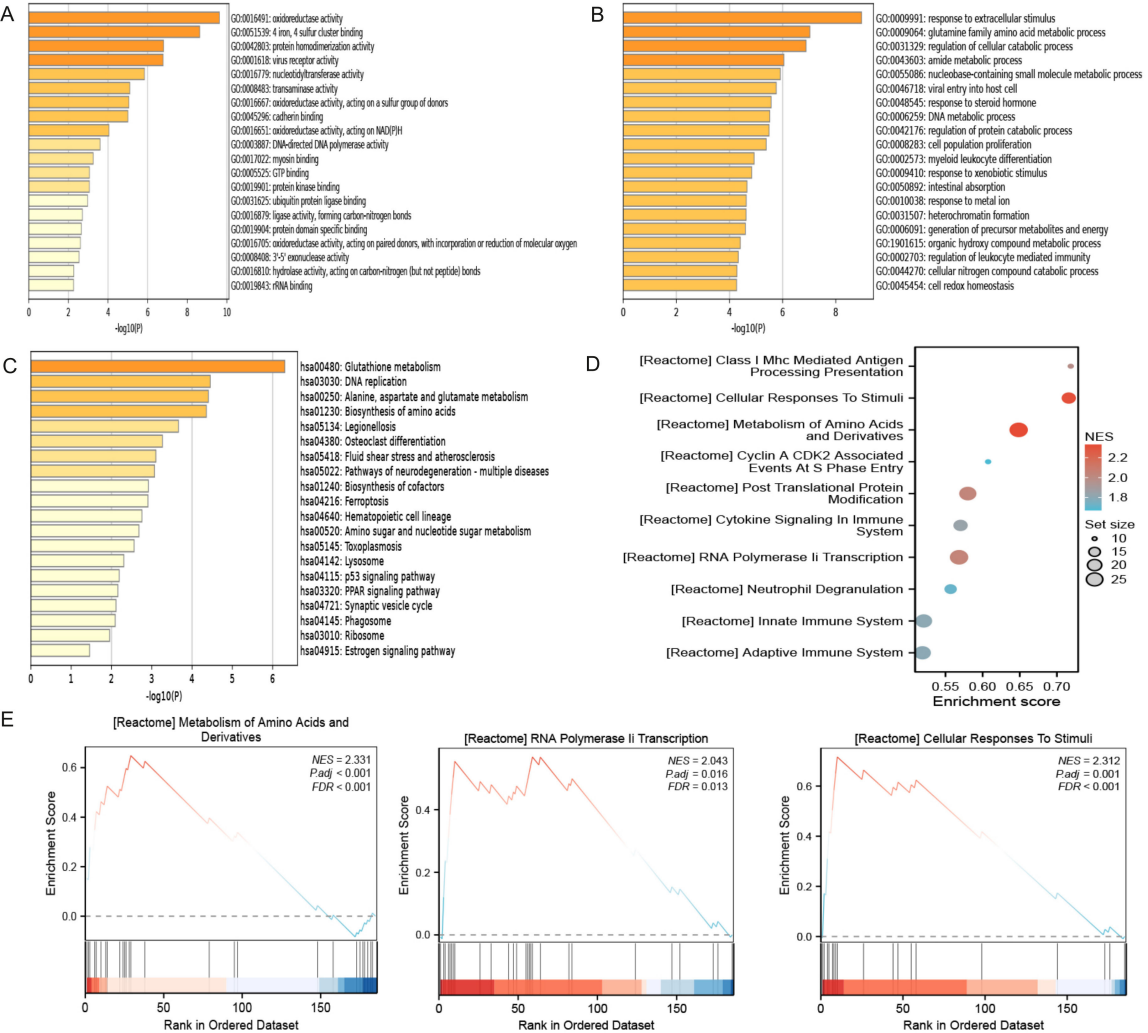
Enrichment terms for differential proteins. **(A)** Molecular functions in GO analysis; **(B)** Biology processes in GO analysis; **(C)** KEGG pathway analysis; **(D)** GSEA enrichment analysis; **(E)** The top three biological processes ranked. KEGG,Kyoto Encyclopedia of Genes and Genomes; GO,Gene Ontology; GSEA,Gene Set Enrichment Analysis.

As shown in **Figure 3A** and **Table 1**, the differentially expressed proteins mainly focus on “oxidoreductase activity,” “protein homodimerization activity,” and “nucleotide transferase activity” in terms of molecular function. As shown in **Figure 3B** and **Table 2**, the differential proteins mainly focus on “response to extracellular stimuli,” “glutamine family amino acid metabolism process,” and “amide metabolism process” in terms of cellular biology processes. Subsequently, these proteins were found to play biological roles in pathways such as “glutathione metabolism,” “alanine, aspartate, and glutamate metabolism,” and “biosynthesis of amino acids” (**Figure 3C**, **Table 3**). Similarly, GSEA indicated that the functions of the differentially expressed proteins were mainly focused on amino acid metabolism and antioxidative stress (**Figure 3D**, **Table 4**). As shown in **Figure 3E**, the three most distinct biological effects were demonstrated by GSEA enrichment analysis. Therefore, the results of the GO and KEGG analyses indicate that the proteins affected by PTL may alter the amino acid metabolism and oxidative stress status in LUAD.

**Table 1:** Enriched GO terms in molecular functions (P<0.05).

ID	Description	Count	P-value
GO:0016491	oxidoreductase activity	21	2.40E-10
GO:0051539	4 iron, 4 sulfur cluster binding	7	2.34E-09
GO:0042803	protein homodimerization activity	17	1.58E-07
GO:0001618	virus receptor activity	7	1.66E-07
GO:0016779	nucleotidyltransferase activity	8	1.45E-06
GO:0008483	transaminase activity	4	7.94E-06
GO:0016667	oxidoreductase activity, acting on a sulfur group of donors	5	8.91E-06
GO:0045296	cadherin binding	10	1.00E-05
GO:0016651	oxidoreductase activity, acting on NAD(P)H	5	8.91E-05
GO:0003887	DNA-directed DNA polymerase activity	3	2.51E-04
GO:0017022	myosin binding	4	5.62E-04
GO:0005525	GTP binding	8	8.71E-04
GO:0019901	protein kinase binding	11	8.91E-04
GO:0031625	ubiquitin protein ligase binding	7	1.07E-03
GO:0016879	ligase activity, forming carbon-nitrogen bonds	3	1.95E-03
GO:0019904	protein domain specific binding	10	2.24E-03
GO:0016705	oxidoreductase activity, acting on paired donors, with incorporation or reduction of molecular oxygen	5	2.45E-03
GO:0008408	3'-5' exonuclease activity	3	2.88E-03
GO:0016810	hydrolase activity, acting on carbon-nitrogen (but not peptide) bonds	4	5.37E-03
GO:0019843	rRNA binding	3	5.50E-03

**Table 2:** Enriched GO terms in biological process (P<0.05).

ID	Description	Count	P-value
GO:0009991	response to extracellular stimulus	17	1.07E-09
GO:0009064	glutamine family amino acid metabolic process	7	9.33E-08
GO:0031329	regulation of cellular catabolic process	17	1.32E-07
GO:0043603	amide metabolic process	17	8.91E-07
GO:0055086	nucleobase-containing small molecule metabolic process	14	1.23E-06
GO:0046718	viral entry into host cell	7	1.74E-06
GO:0048545	response to steroid hormone	10	2.69E-06
GO:0006259	DNA metabolic process	16	3.02E-06
GO:0042176	regulation of protein catabolic process	11	3.24E-06
GO:0008283	cell population proliferation	15	4.17E-06
GO:0002573	myeloid leukocyte differentiation	7	1.17E-05
GO:0009410	response to xenobiotic stimulus	11	1.45E-05
GO:0050892	intestinal absorption	4	2.19E-05
GO:0010038	response to metal ion	10	2.34E-05
GO:0031507	heterochromatin formation	5	2.40E-05
GO:0006091	generation of precursor metabolites and energy	10	2.51E-05
GO:1901615	organic hydroxy compound metabolic process	11	3.89E-05
GO:0002703	regulation of leukocyte mediated immunity	8	4.68E-05
GO:0044270	cellular nitrogen compound catabolic process	9	5.25E-05
GO:0045454	cell redox homeostasis	4	5.37E-05

**Table 3:** Enriched GO terms in KEGG pathways(P<0.05).

ID	Description	Count	P-value
hsa00480	Glutathione metabolism	6	5.01E-07
hsa03030	DNA replication	4	3.55E-05
hsa00250	Alanine, aspartate and glutamate metabolism	4	3.98E-05
hsa01230	Biosynthesis of amino acids	5	4.47E-05
hsa05134	Legionellosis	4	2.19E-04
hsa04380	Osteoclast differentiation	5	5.50E-04
hsa05418	Fluid shear stress and atherosclerosis	5	7.94E-04
hsa05022	Pathways of neurodegeneration - multiple diseases	9	8.51E-04
hsa01240	Biosynthesis of cofactors	5	1.20E-03
hsa04216	Ferroptosis	3	1.23E-03
hsa04640	Hematopoietic cell lineage	4	1.74E-03
hsa00520	Amino sugar and nucleotide sugar metabolism	3	2.09E-03
hsa05145	Toxoplasmosis	4	2.75E-03
hsa04142	Lysosome	4	4.90E-03
hsa04115	p53 signaling pathway	3	6.46E-03
hsa03320	PPAR signaling pathway	3	6.92E-03
hsa04721	Synaptic vesicle cycle	3	7.76E-03
hsa04145	Phagosome	4	8.13E-03
hsa03010	Ribosome	4	1.12E-02
hsa04915	Estrogen signaling pathway	3	3.47E-02

**Table 4:** Gene set enrichment analysis.

ID	Set size	Enrichment score	NES	pvalue	p.adjust	qvalue
REACTOME_METABOLISM_OF_AMINO_ACIDS_AND_DERIVATIVES	26	0.6485253	2.331420	1.39e-05	0.0008	0.0006
REACTOME_CELLULAR_RESPONSES_TO_STIMULI	17	0.7160420	2.312042	3.88e-05	0.0011	0.0009
REACTOME_RNA_POLYMERASE_II_TRANSCRIPTION	26	0.5682348	2.042779	0.0009	0.0162	0.0131
REACTOME_POST_TRANSLATIONAL_PROTEIN_MODIFICATION	23	0.5800484	2.036427	0.0013	0.0179	0.0144
REACTOME_CLASS_I_MHC_MEDIATED_ANTIGEN_PROCESSING_PRESENTATION	10	0.7186957	1.976948	0.0020	0.0216	0.0174
REACTOME_CYTOKINE_SIGNALING_IN_IMMUNE_SYSTEM	17	0.5703051	1.841469	0.0105	0.0962	0.0773
REACTOME_INNATE_IMMUNE_SYSTEM	22	0.5206910	1.803542	0.0150	0.1076	0.0865
REACTOME_ADAPTIVE_IMMUNE_SYSTEM	22	0.5190075	1.797710	0.0157	0.1076	0.0865
REACTOME_NEUTROPHIL_DEGRANULATION	14	0.5568813	1.712733	0.0303	0.1827	0.1469
REACTOME_CYCLIN_A_CDK2_ASSOCIATED_EVENTS_AT_S_PHASE_ENTRY	10	0.6073959	1.670791	0.0366	0.1827	0.1469

### 3.3 Targeted amino acid metabolomics shows that the amino acid content of LUAD cells was influenced by PTL

**Figure 4:**
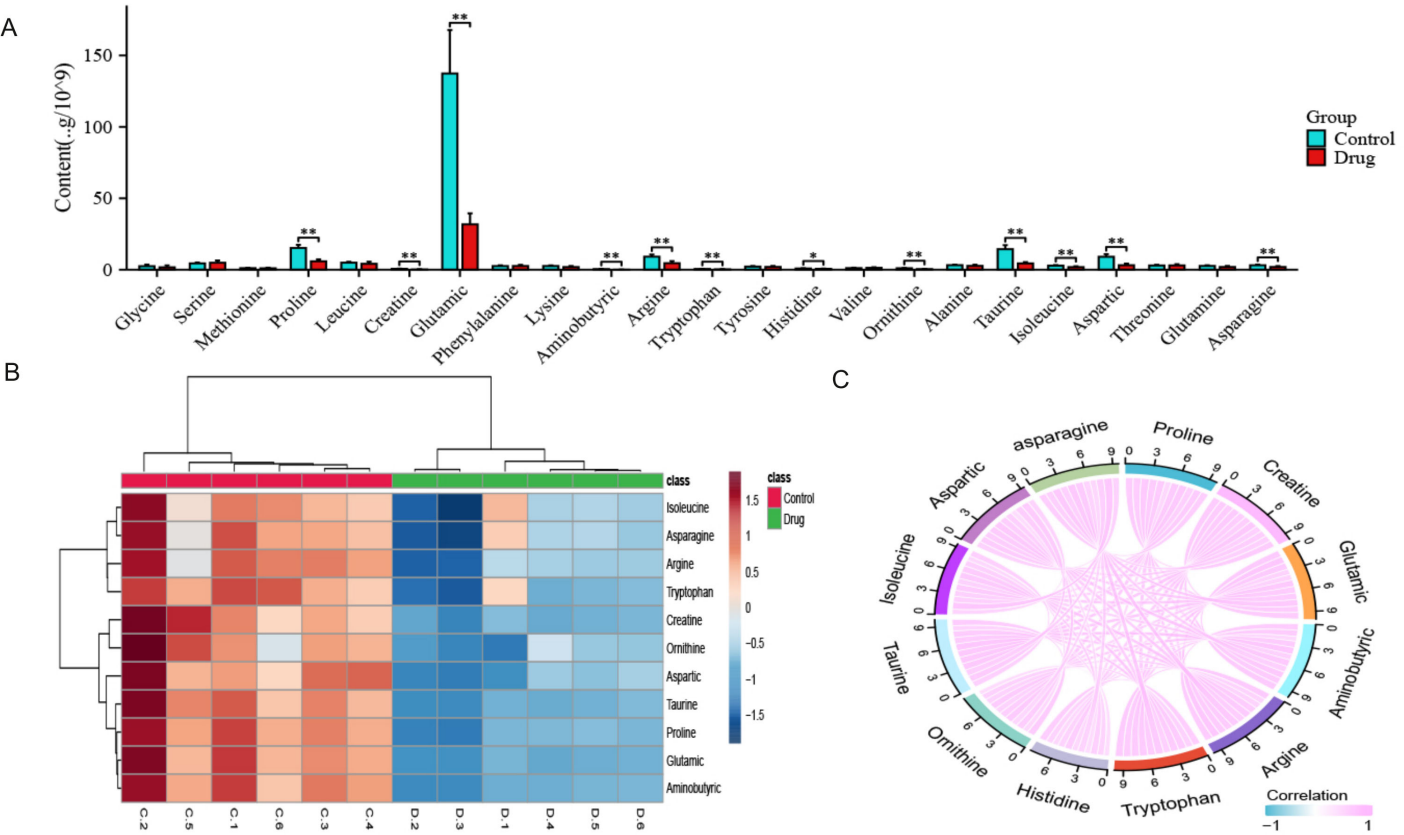
Quantitative results of 23 amino acids after PTL action on lung adenocarcinoma cells. **(A)** Significant differences were observed in 12 amino acids after PTL administration; **(B)** Cluster analysis shows that significantly altered amino acids have similar metabolic patterns; **(C)** Correlation analysis shows a positive correlation between 12 amino acids. Ns stands for p ≥ 0.05, ∗p < 0.05, ∗∗p < 0.01. The concentration of PTL was 10 μM. PTL, Parthenolide; Ns, non-significant. The experiment included six biological replicates.

As shown in **Figure 4A**, 23 amino acids were detected in this study; we found significant changes in the content of 12 amino acids after PTL administration (**Supplementary Tables S3**). Interestingly, all 12 amino acids significantly decreased after PTL administration. As shown in **Figure 4B**, these significantly altered amino acids formed a cluster, indicating that they had similar metabolic patterns and may be closely related to energy metabolism. Correlation analysis was performed to determine the degree of association between significant amino acids, with the aim of gaining a deeper understanding of the relationships between amino acid types in biological processes. Correlation analysis showed that each amino acid was significantly positively correlated with the other 11 amino acids (P<0.05,r>0.7) (**Figure 4C**). These results indicate that PTL reduces amino acid production in LUAD, thereby affecting energy metabolism.

### 3.4 The results of multivariate statistical analysis validate the significant differences in metabolites after administration

**Figure 5:**
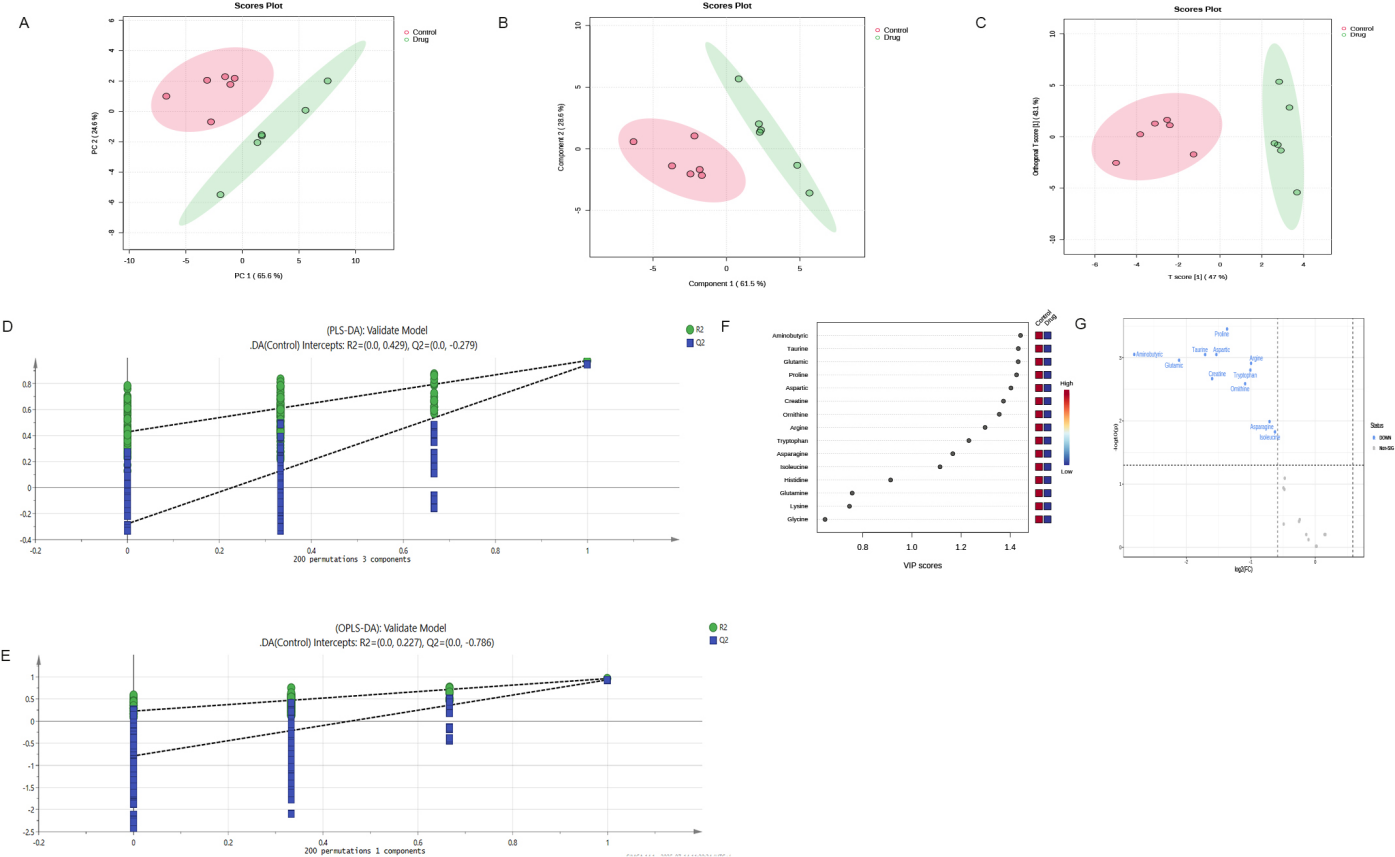
Multivariate statistical analysis confirm significant differences in metabolites after administration **(A)** PCA scoring chart; **(B)** PLS-DA scoring chart; **(C)** OPLS-DA scoring chart; **(D, E)** External permutation test; **(F)** The Variable Importance in Projection scores; **(G)** The volcano map indicates that these metabolites enhance the explanatory power of the model. OPLS-DA,orthogonal partial least squares discriminant analysis; PLS-DA,partial least squares discriminant analysis; PCA, principal component analysis.

From the PCA scoring chart, it was concluded that there was good repeatability within the groups, the sample data were very similar, and there was good discrimination between the groups (**Figure 5A**). Similarly, the PLS-DA and OPLS-DA models (**Figures 5B, C**) showed significant differences between the two groups. External permutation test was performed to avoid overfitting, with a R2 value of 0.429, a Q2 value of -0.279 and with a R2 value of 0.227, a Q2 value of -0.786, respectively (**Figures 5D, E**). The Variable Importance in Projection (VIP) scores showed that 11 significantly differentially expressed amino acid metabolites had scores greater than 1, indicating that these metabolites enhanced the explanatory power of the model (**Figure 5F**, **Table 5**). The volcano map consistently showed similar results (**Figure 5G**).

**Table 5:** Eleven amino acid components significantly altered by treatment of LUAD cells with parthenolide.

Component	RT(min)	Fold change	*P*-value	VIP
Aminobutyric Acid	2.26	0.14281	5.26E-05	1.442167
Glutamate	3.28	0.2313	5.26E-05	1.432625
Taurine	2.62	0.306	5.26E-05	1.433051
Proline	2.53	0.38733	5.26E-05	1.425923
Aspartic	3.69	0.34537	0.00023465	1.403259
Creatine	2.83	0.32981	0.00026181	1.372701
Tryptophan	1.96	0.49779	0.00086656	1.231825
Arginine	4.87	0.50144	0.00091973	1.297919
Ornithine	5.09	0.47054	0.0013757	1.355667
Asparagine	3.61	0.61136	0.0089733	1.166506
Isoleucine	2.07	0.64867	0.012337	1.114533

LUAD, lung adenocarcinoma; RT, retention time; VIP, variable importance in the projection.

### 3.5 Network pharmacology analysis to screen the targets of PTL on LUAD

**Figure 6:**
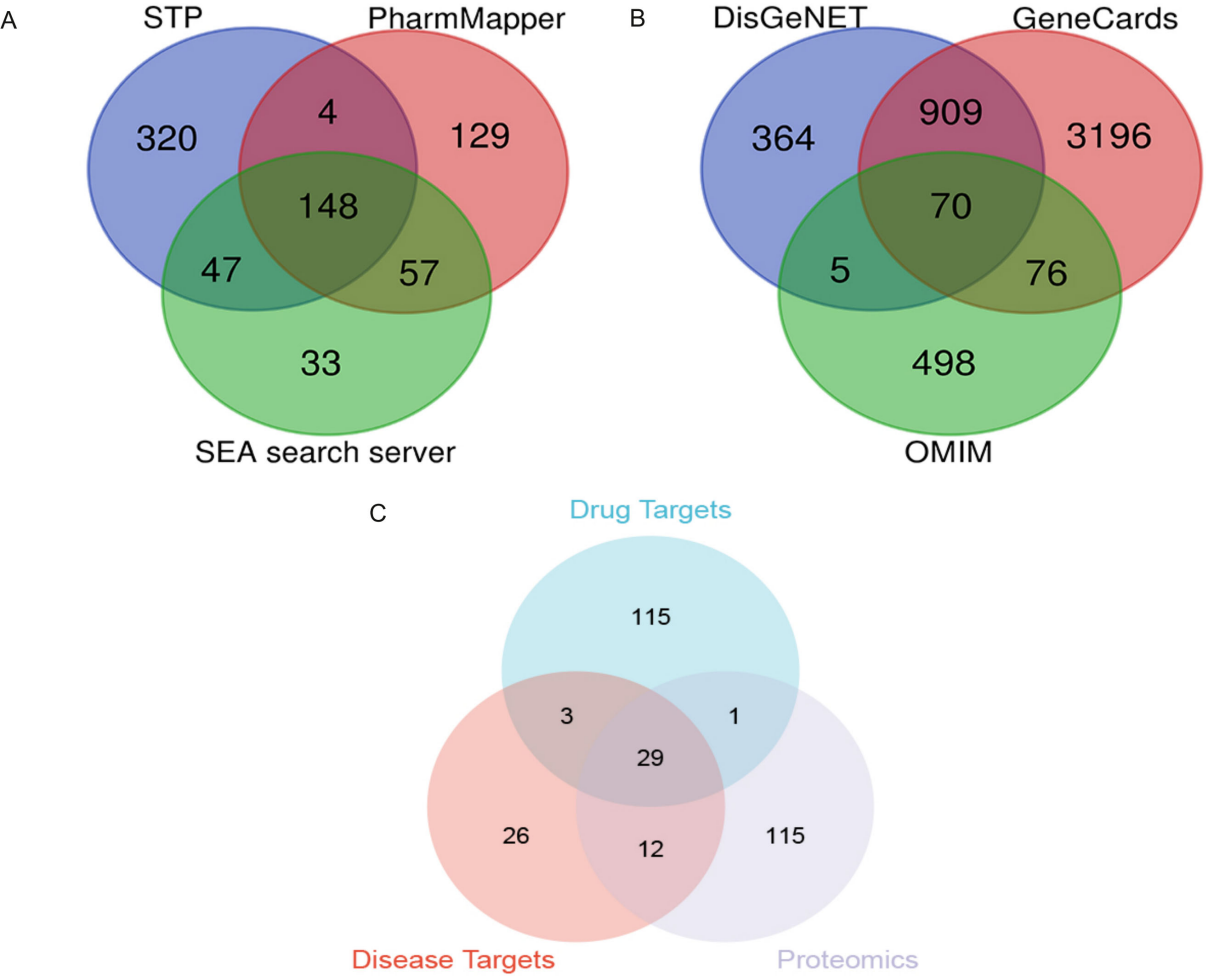
Screening of PTL targets through network pharmacology analysis **(A)** Screening PTL targets based on public databases; **(B)** Screening targets for lung adenocarcinoma based on public databases; **(C)** Venn diagram shows 29 potential targets of PTL acting on lung adenocarcinoma. PTL, Parthenolide.

Based on the SwissTarget Prediction (STP), SEA search server, and PharmMapper databases, 148 target genes were associated with PTL (**Figure 6A**). Based on DisGeNET, GeneCards Search, and OMIM databases, 70 target proteins were associated with LUAD (**Figure 6B**). When these selected genes were crossed with differentially expressed genes using quantitative proteomics, 29 potential targets of PTL against LUAD were identified (**Figure 6C**).

**Figure 7:**
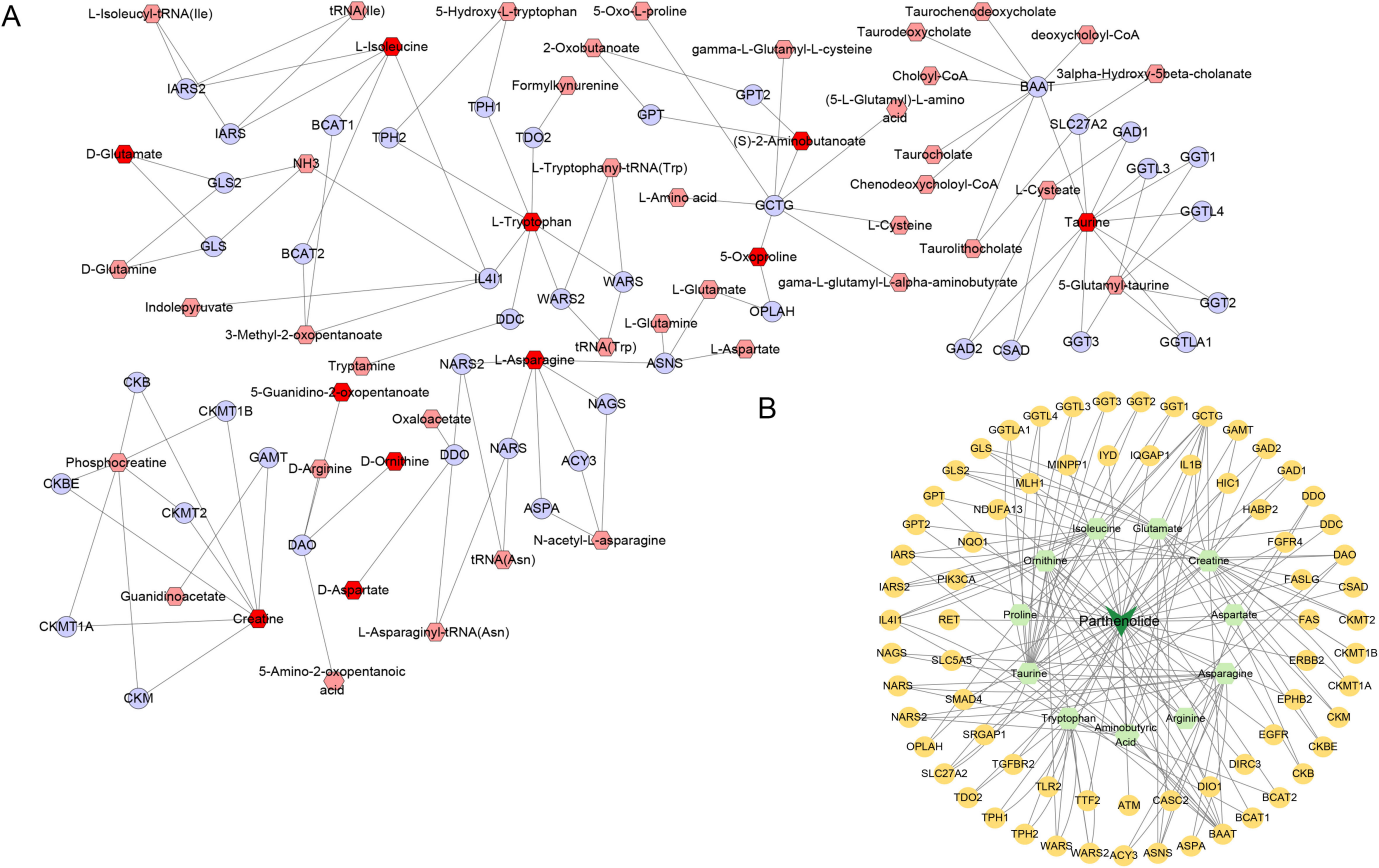
Screening of 11 amino acid metabolite targets regulated by PTL using Cytoscape 3.9.1 software. **(A)** Forty-one amino acid metabolite targets were obtained using MetScape software; **(B)** A compound-target-metabolites network diagram was constructed.

Subsequently, in Cytoscape 3.9.1, 11 different amino acid metabolites regulated by PTL were introduced into MetScape and 41 amino acid metabolite targets were obtained (**Figure 7A**). Finally, a compound-target-metabolite network consisting of 82 nodes (PTL, 70 related target genes, and 11 amino acid metabolites) and 153 edges was constructed (**Figure 7B**). Based on parameters such as degree, betweenness centrality, proximity, and stress in the network, GCTG (also known as GGCT), DAO, and BAAT were identified as key targets (**Table 6**).

**Table 6:** The top 8 nodes based on degree, betweenness, closeness, and stress.

Rank	Gene game	Degree	Gene game	Betweenness	Gene game	Closeness	Gene game	Stress
1	BAAT	9	BAAT	0.02	BAAT	0.378	GCTG	846
2	GCTG	8	GCTG	0.017	GCTG	0.367	BAAT	788
3	IL4I1	5	DAO	0.011	DAO	0.359	DAO	496
4	DAO	4	ASNS	0.007	TDO2	0.348	TDO2	266
5	ASNS	4	TDO2	0.006	GAMT	0.348	GAMT	266
6	SLC27A2	3	GAMT	0.006	SLC27A2	0.342	IL4I1	194
7	GLS	3	SLC27A2	0.0037	GAD1	0.342	SLC27A2	182
8	GLS2	3	GAD1	0.0037	GAD2	0.342	GAD1	182

### 3.6 Molecular docking analysis of PTL binding to potential targets

**Figure 8:**
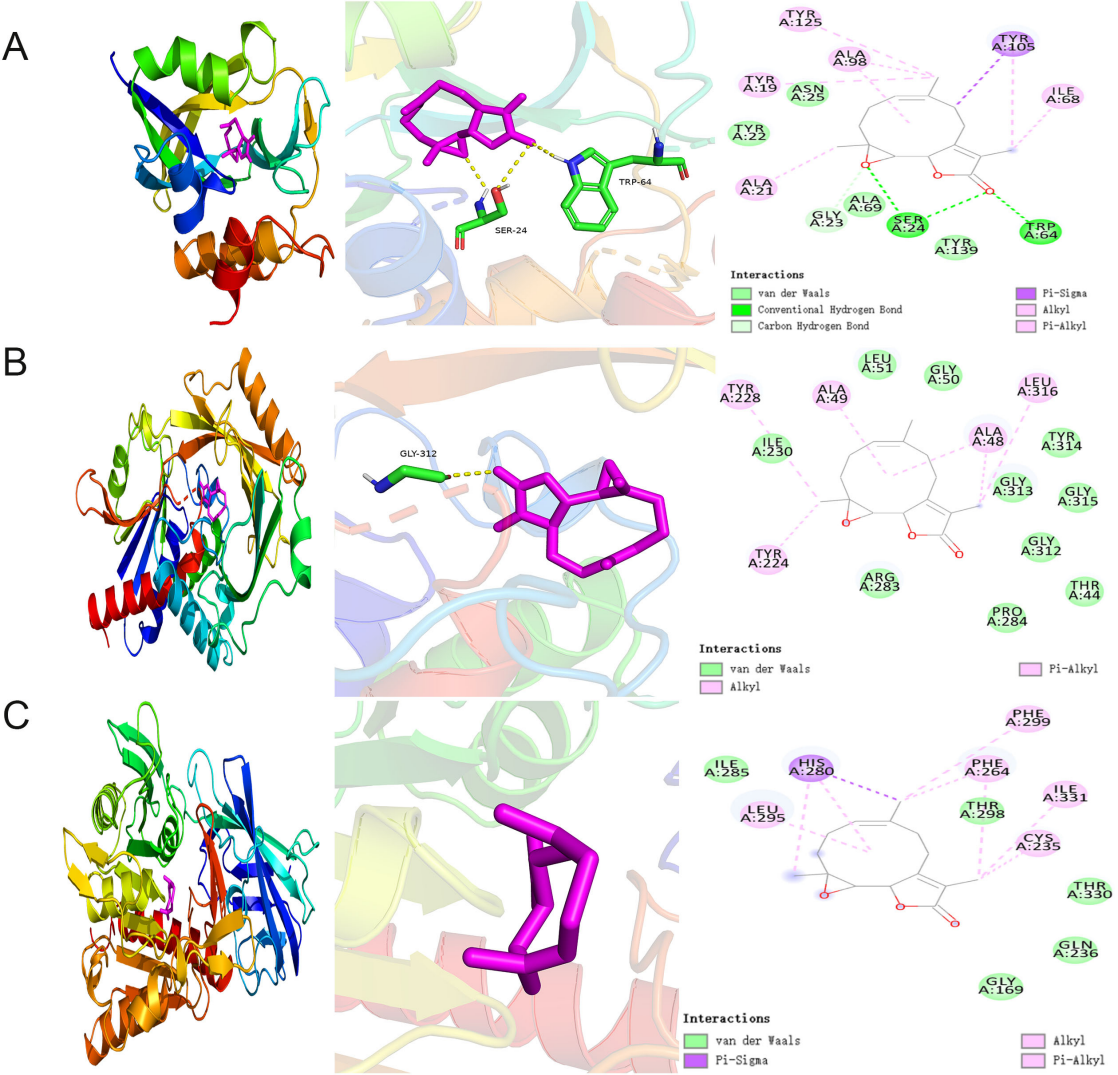
Molecular docking analysis of PTL binding to potential targets **(A–C)** Molecular docking analysis demonstrated the binding of GCTG, DAO, and BAAT to PTL, respectively. GCTG, Gamma-Glutamylcyclotransferase; DAO, D-Amino Acid Oxidase; BAAT, Bile Acid-CoA : Amino Acid N-Acyltransferase; PTL, Parthenolide.

We conducted molecular docking studies using AutoDock Vina to further elucidate the interactions between PTL and potential targets. Docking analysis of GCTG showed that PTL formed hydrogen bonds with the SER A: 24 and TRP A: 64 residues at the active site. The van der Waals interactions included TYR A: 22, ASN A:25, ALA A: 69, and TYR A:139 residues. The binding energy between GCTG and PTL was -10.5 (**Figure 8A**). However, in the molecular docking analysis of DAO and BAAT with PTL, there was no interaction with hydrogen bonds, and the binding energies of the two were -8.5 and -7, respectively (**Figures 8B, C**). In summary, GCTG had the lowest binding energy for molecular docking with PTL and hydrogen-bonding interactions. Therefore, GCTG was selected as a key target for subsequent experiments.

### 3.7 Molecular dynamics analysis shows that there is stable binding between PTL and GCTG

**Figure 9:**
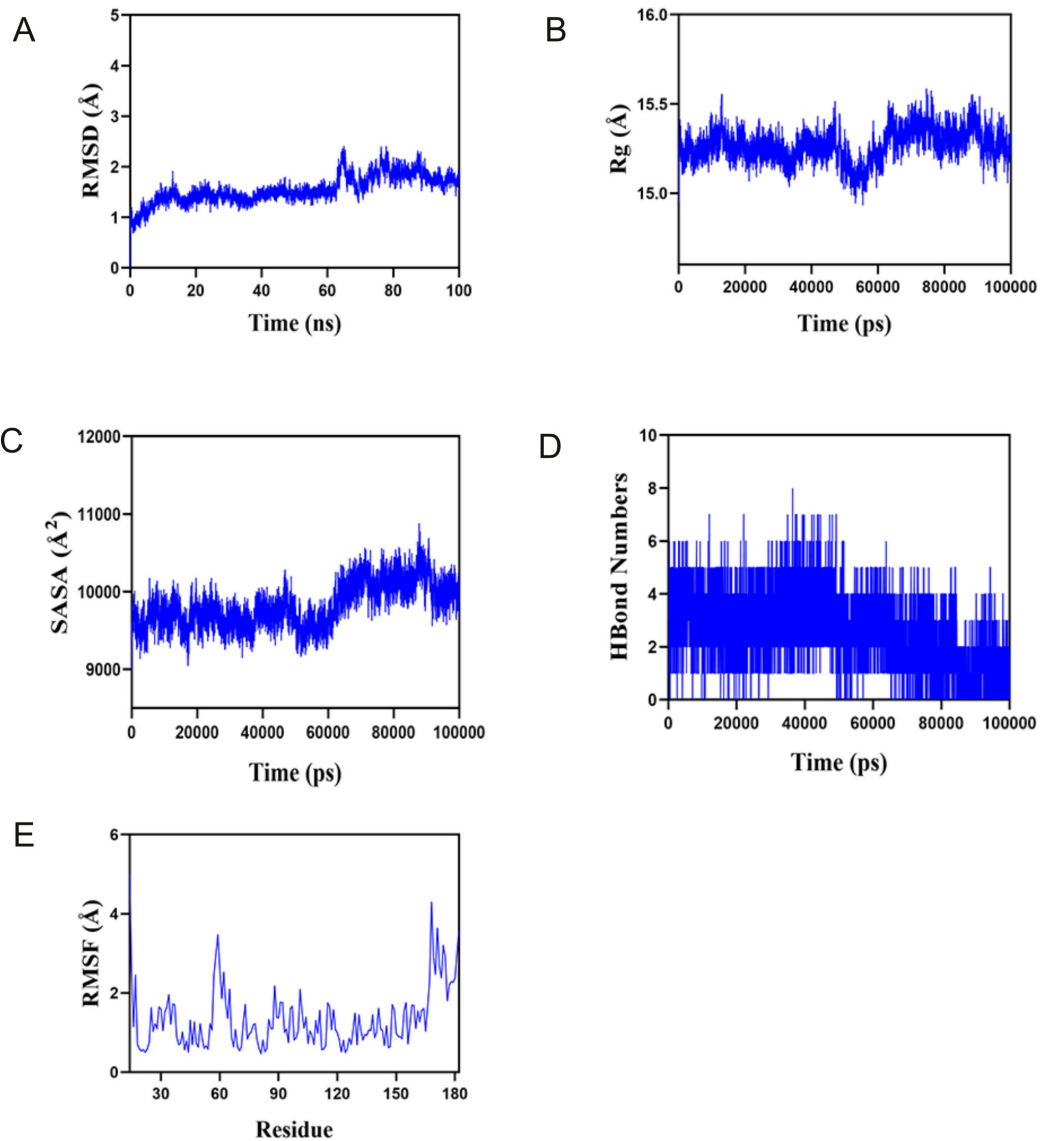
Molecular dynamics analysis confirms the stable binding between PTL and GCTG **(A)** Root mean square deviation (RMSD); **(B)** Radius of gyration (Rg); **(C)** Solvent accessible surface area; **(D)** Hydrogen bond numbers; **(E)** Root mean square fluctuation (RMSF). PTL, Parthenolide.

First, the balance of the simulation system was evaluated using root mean square deviation (RMSD). Generally speaking, an RMSD value less than 2 Å and reaching stability within 10–20 ns indicates high conformational stability of the system. As shown in **Figure 9A**, the composite system reached equilibrium at around 20ns and eventually fluctuated at approximately 1.8 Å. Therefore, PTLs exhibit high stability when bound to a target protein. Further analysis revealed that the radius of gyration (Rg) and solvent-accessible surface area (SASA) of the composite system fluctuated stably during motion, indicating that the composite system remained stable and compact throughout the simulation (**Figures 9B, C**). The number of hydrogen bonds between PTL and the target protein during the dynamic process is shown in **Figure 9D**, and the number of hydrogen bonds between the complex system ranged from 0 to 8, indicating that the complex system had good hydrogen bond interactions. As shown in **Figure 9E**, the root mean square fluctuation (RMSF) of the composite was relatively low (mostly below 2 Å), resulting in low flexibility and high stability. In summary, the binding of the complex was stable, and the complex exhibited good hydrogen bonding properties. Therefore, PTL bind well to its target proteins.

### 3.8 PTL targeting GCTG affects the proliferation, amino acid metabolism, and oxidative stress levels in LUAD

**Figure 10:**
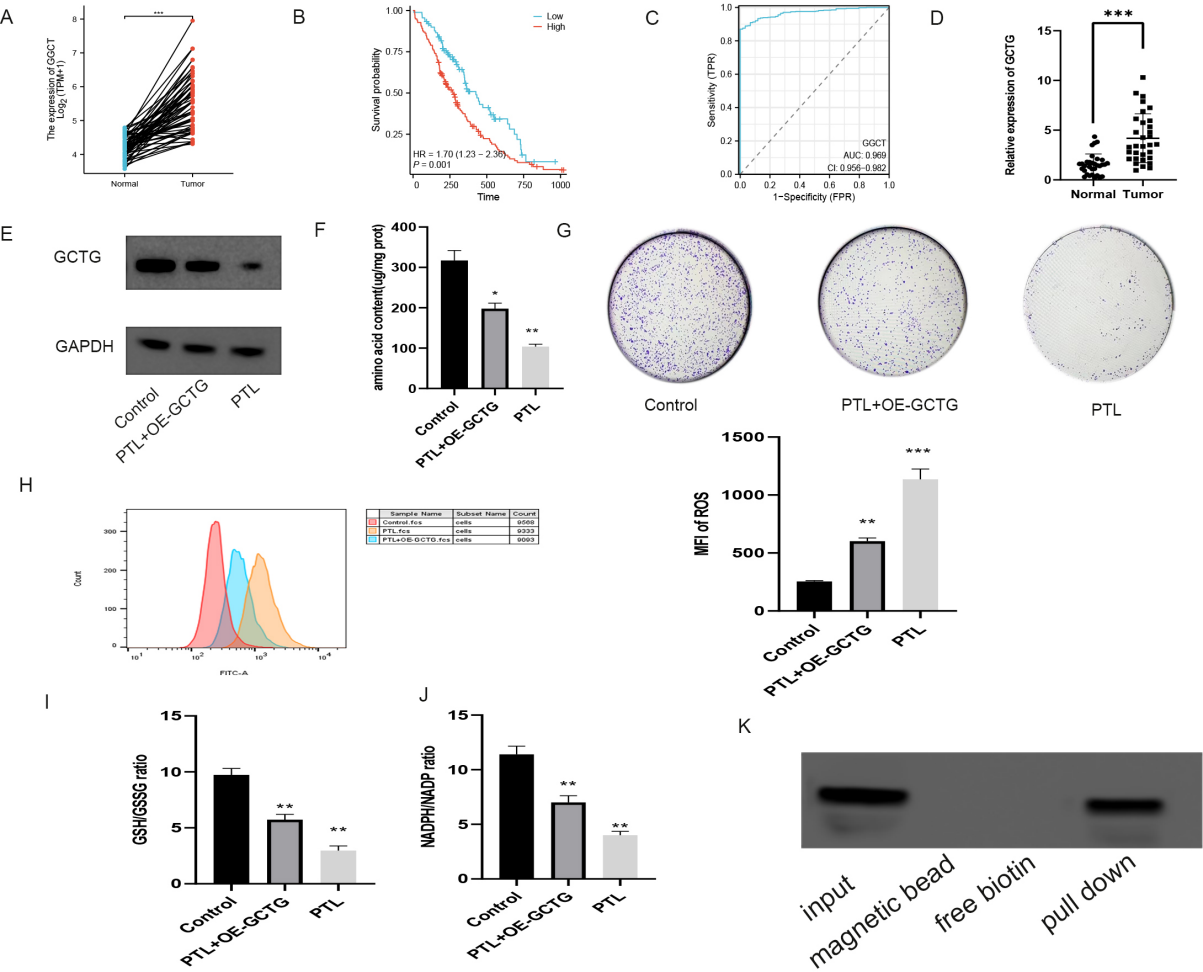
*In vitro* experiment on the effect of PTL targeting GCTG in lung adenocarcinoma. **(A)** High expression of GCTG in tumor tissues in TCGA database; **(B)** High expression of GCTG is significantly associated with poor prognosis; **(C)** ROC curve analysis for GCTG expression in LUAD and paracancerous tissues; **(D)** GCTG is highly expressed in tumor tissues of 32 pairs of samples; **(E)** PTL inhibits the expression of GCTG protein; **(F)** PTL targets GCTG to inhibit the total amino acid content of lung adenocarcinoma cells; **(G)** PTL targets GCTG to inhibit the proliferation of lung adenocarcinoma cells; **(H)** PTL targets GCTG to promote the increase of ROS in lung adenocarcinoma cells; **(I)** PTL targets GCTG to inhibit GSH/GSSG ratio; **(J)** PTL targets GCTG to inhibit NADPH/NADP ratio. **(K)** Pull down assay confirms that PTL directly acts on GCTG. Ns stands for p ≥ 0.05, ∗p < 0.05, ∗∗p < 0.01, ∗∗∗p < 0.001. The concentration of PTL was 10 μM. PTL, Parthenolide. All the above *in vitro* experiments were conducted with three biological replicates.

As shown in **Figures 10A–C**, bioinformatics analysis revealed that GCTG was significantly upregulated in tumor tissues and could serve as a potential biomarker for predicting prognosis. High expression was significantly associated with poor prognosis. RT-qPCR results of 32 clinical samples also showed that GCTG was significantly overexpressed in cancer tissues (**Figure 10D**). As shown in **Table 7**, GCTG expression significantly correlated with tumor size and staging. Therefore, both dry and wet experiments indicated that GCTG was significantly upregulated and had a pro-cancer effect in LUAD. As shown in (**Figure 10E**), compared to that in the control group, PTL significantly inhibited the expression of GCTG protein, whereas the rescue experiment partially reversed the expression of this protein. As shown in **Figures 10F, G**, PTL significantly inhibited the proliferation and amino acid metabolism of LUAD cells, whereas rescue experiments partially reversed these biological processes. Correspondingly, as shown in **Figures 10H–J**, PTL targeting GCTG enhanced ROS expression while inhibiting the GSH/GSSG and NADPH/NADP ratios. To further confirm whether PTL has a direct mechanism of action on GCTG, a pull-down assay was performed, and it was found that PTL directly regulates GCTG (**Figure 10K**)

**Table 7:** Relationship between GCTG and clinical pathological characteristics of lung adenocarcinoma patients.

Clinical pathological characteristics	RT-qPCR		P-value
	Low(n=16)	High(n=16)	
Sex, n			
male	12	13	0.685
female	4	3	
Age, years			
≤60	8	9	0.987
>60	8	7	
Smoke			
Yes	10	8	0.722
No	6	8	
Tumor size, cm			
≤5	13	2	0.001
>5	3	14	
Lymph node metastasis			
Yes	9	10	0.961
No	7	6	
TNM stage, n			
I	10	2	0.023
II-III	6	14	

**Figure 11:**
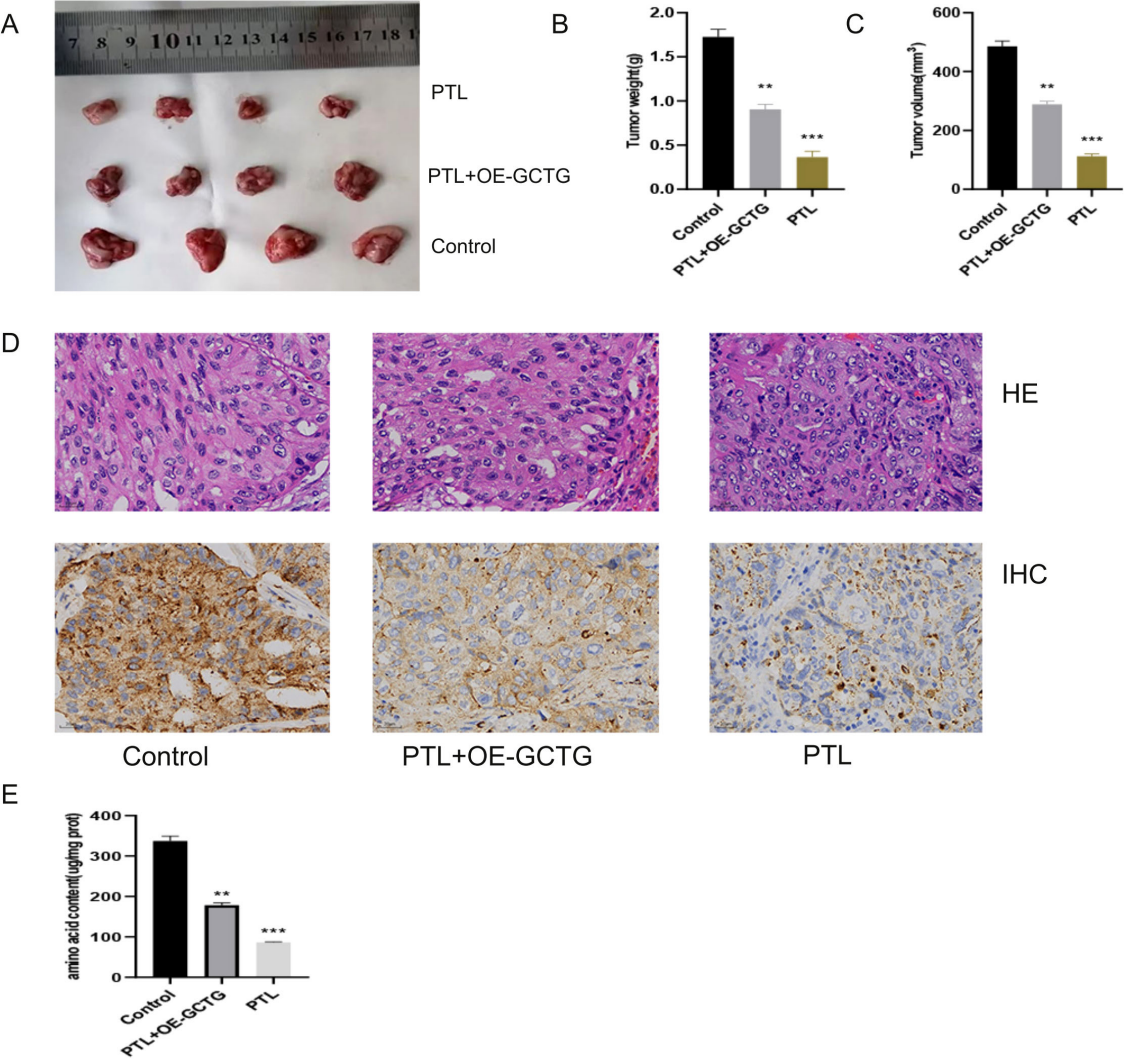
*In vivo* experiment of PTL targeting GCTG to regulate lung adenocarcinoma. **(A–C)** PTL targets GCTG to inhibit the proliferation of lung adenocarcinoma; **(D)** Immunohistochemical experiments show that PTL inhibits the expression of GCTG; **(E)** PTL targeting GCTG inhibits the content of total amino acids in lung adenocarcinoma tissue. Ns stands for p ≥ 0.05, ∗∗p < 0.01, ∗∗∗p < 0.001. The intraperitoneal injection dose of PTL was 20 mg/kg. PTL, Parthenolide. *In vivo* experiments included four biological replicates.

As shown in **Figure 11A**, nude mice were euthanized and their tumors were removed for photography. PTL significantly decreased tumor weight and volume (**Figures 11B, C**). Immunohistochemical analysis revealed that PTL inhibited GCTG expression (**Figure 11D**). The amino acid content determination experiment also showed that PTL inhibited amino acid metabolic reprogramming in LUAD tissues (**Figure 11E**). The aforementioned rescue experiments partially reversed this phenomenon.

## 4 Discussion

PTL is a natural compound, extracted from the small white chrysanthemum (*Chrysanthemus morifolium*) and has received widespread attention in recent years owing to its various biological activities (27, 28). It has been confirmed that pyrethrum lactone has many effects, such as antioxidant, anti-inflammatory, and immune regulation effects, and has significant therapeutic effects on pulmonary hypertension, diabetes, and certain types of infectious diseases (29–31). PTL also exhibits promising anti-tumor properties, particularly through its interactions with critical signaling pathways such as NF-kB and STAT3, which regulate cell survival and apoptosis (32–36). However, studies on the effects of PTL on LUAD have predominantly focused on these pathways, neglecting the potential role of metabolic reprogramming and oxidative stress in tumor progression. This study aimed to bridge this gap by investigating the metabolic alterations and oxidative stress imbalance induced by PTL in LUAD cells.

To the best our knowledge, this is the first study to investigate the differential protein expression in LUAD cells treated with PTL using PRM technology. Subsequently, through GO and KEGG analyses, differentially expressed proteins were successfully identified in biological processes related to amino acid metabolism. Based on the above proteomic results, we found that 12 of the 23 amino acids were significantly differentially expressed in PTL-treated cells. The combination of the multiple omics experimental methods mentioned above can not only shorten the research time but also accurately position our research direction towards amino acid metabolism reprogramming and oxidative stress.

To further investigate the molecular mechanisms underlying the effects of PTL on amino acid metabolism and oxidative stress in LUAD, we used network pharmacology and molecular docking to explore its downstream targets. Results identified GCTG as a potential site of action for the alteration of amino acid metabolism and oxidative stress in PTL-induced LUAD. γ-glutamyltransferase (GCTG, also known as GGCT) is an important enzyme in glutathione metabolism, catalyzing the reaction of gamma glutamyl peptide to produce 5-hydroxyproline and free amino acids (37, 38). GCTG is highly conserved in many species including bacteria, plants, and other higher organisms (39). Reportedly, GCTG is highly expressed in a variety of cancers, including breast cancer, lung cancer, bladder cancer, colon cancer, osteosarcoma and glioma, suggesting that it may be the oncogene in these tumors (40–43). Moreover, GCTG also promotes the malignant behavior of tumors (44–46). Because GCTG is a crucial catalytic enzyme in amino acid metabolism and its high expression in LUAD tissue was confirmed in clinical specimens, we speculate that PTL inhibits GCTG and thus affects the amino acid reprogramming of cancer cells. In addition, GSEA analysis suggested that PTL can cause changes in oxidative stress-related pathways in tumor cells; therefore, experiments detecting ROS expression levels were also included in the study.

To confirm this hypothesis, both *in vivo* and *in vitro* experiments were conducted. Our results indicate that PTL delays tumor growth and amino acid metabolism and disrupts the oxidative stress balance by inhibiting GCTG expression. Amino acid metabolism, an important component of energy metabolic reprogramming, has attracted much attention in the field of cancer. Physapubescin I, a natural compound extracted from edible herbaceous fruits, increases intracellular glutamine levels and correspondingly reduces glutamate and its downstream metabolites, thereby inhibiting cell proliferation and inducing apoptosis (47). Ophiopogon is a tetracyclic diterpenoid derived from plants belonging to the Lamiaceae and Camellia genera. Reportedly, oridonin reverses the resistance of pancreatic cancer cells, PANC-1/Gem, to gemcitabine by inhibiting glutathione S-transferase (GST pi) (48). In addition, oxidative stress is also one of the hotspots in the field of cancer. Baicalin induces ROS production in bladder cancer cells, thereby enhancing the expression of apoptosis-related proteins (49). Resveratrol has a two-way regulatory effect on tumors: low dose activates SIRT1 to maintain oxidative stability, whereas high dose induces mitochondrial ROS bursts. This results in the reversal of chemotherapeutic resistance in stem cells (50). Overall, there has been relatively little research on the effects of traditional Chinese medicine monomers on amino acid metabolism in tumors. However, there is currently no study on the impact of PTL on energy metabolism and oxidative stress in LUAD. Therefore, this study explored the effects of PTL on amino acid metabolism, oxidative stress, and downstream molecular mechanisms in LUAD using through multi omics/network, pharmacology/molecular simulation docking, and experimental validation methods. These findings provide valuable insights into the therapeutic potential of PTL, not only as a standalone treatment but also in combination with existing therapies, thereby enhancing the overall treatment efficacy and improving patient outcomes. The significance of this study lies in its potential to contribute to a more comprehensive understanding of LUAD treatment, paving the way for innovative approaches that address both the biological and metabolic aspects of tumor growth. Interestingly, PTL was previously considered an antioxidant in non-tumor studies; however, in this study, it was found to be an oxidant. This may be due to changes in the nature of PTL caused by different types of diseases.

This study has some limitations. First, the three basic forms of energy metabolism often occur simultaneously in tumors. Therefore, in addition to amino acid metabolism, future studies should investigate the effects of PTL on lipid and glucose metabolisms. Second, to make our results more specific, further research is required to determine which specific amino acids are affected by PTL targeting GCTG. Finally, the relationship between amino acid metabolism and oxidative stress requires further investigation.

## 5 Conclusion

This is the first study to report the impact of PTL on amino acid metabolic reprogramming and oxidative stress in LUAD. We also explored the effects of PTL on the downstream target, GCTG, using multiple omics, network pharmacology, and wet experiments. This study provides a potential new mechanism by which PTL delays the proliferation of LUAD; however, further research is warranted.

## Data Availability

The data presented in the study are deposited in the iProx (https://www.iprox.cn/page/home.html), accession numbers IPX0013067000 and PXD067441.
