# Supplementary material for: Effects of parthenolide on amino acid metabolism and oxidative stress in lung adenocarcinoma based on quantitative proteomic analysis, targeted amino acid metabolomics, network pharmacology, and experimental validation
Source: Front Oncol. 2025 Sep 1;15:1642866. doi: 10.3389/fonc.2025.1642866 (PMC12433850; doi:10.3389/fonc.2025.1642866)
Supplement: Supplementary file 1 [file Table1.docx]

differential proteins

| Gene.names | log2FC | pvalue |
| --- | --- | --- |
| COX2;COII;MT-CO2;cox2 | 0.290818497 | 0.02526822 |
| FLJ10292;MAGOHB | -0.016674395 | 0.890825009 |
| RBM8;RBM8A | 0.079384051 | 0.468775435 |
| TM9SF4 | 0.125543782 | 0.198113208 |
| TM9SF2 | 0.182571448 | 0.213799482 |
| SMBP;TM9SF3 | 0.1325265 | 0.200295967 |
| AP1S1 | -0.019817262 | 0.89045505 |
| EIF3C;EIF3CL | 0.057589529 | 0.588716241 |
| RBM3 | -0.256455759 | 0.052756197 |
| PRPSAP2 | -0.090150158 | 0.366703663 |
| SDHA | -0.115948102 | 0.458971513 |
| FLOT2;hCG_1998851 | -0.049620363 | 0.788716241 |
| ALDOC | 0.029915519 | 0.801368849 |
| RAB34 | -0.267644027 | 0.042434332 |
| EFHD2 | -0.064186796 | 0.634443211 |
| EPHA2 | -0.10462412 | 0.292156863 |
| CD2BP2 | -0.043265827 | 0.70836108 |
| RPL18 | 0.014361154 | 0.882500925 |
| SNRP70;SNRNP70 | 0.102599889 | 0.36918239 |
| NUP62;hCG_19665 | -0.024737775 | 0.803033666 |
| RRAS | 0.606907699 | 0.400887902 |
| CCDC6 | 0.028092171 | 0.799371069 |
| HK1 | 0.079669691 | 0.509211987 |
| HNRPH3;HNRNPH3 | -0.160710436 | 0.196596374 |
| SEC24C | 0.044319714 | 0.677580466 |
| hCG_2024613;DNAJC9 | 0.078261382 | 0.588605253 |
| VCL;HEL114 | 0.002203921 | 0.971513134 |
| CDC2;DKFZp686L20222;CDK1 | -0.309597709 | 0.024232334 |
| PSAP | 0.464203637 | 0.024787273 |
| hCG_2002731;PELO | -0.178228543 | 0.16263411 |
| DKFZp586G1518;AP3M1 | 0.109911989 | 0.26400296 |
| PPIF | 0.152297534 | 0.163965964 |
| VDAC2 | 0.145226282 | 0.158416574 |
| EXOC2 | 0.052920709 | 0.715982242 |
| ECI2;PECI;HCA64 | -0.52997523 | 0.015982242 |
| NUP153 | 0.155463825 | 0.268627451 |
| SERPINB6 | -0.064756097 | 0.727192009 |
| JTV1;AIMP2 | 0.08223638 | 0.431335553 |
| PRPF4B | -0.175304892 | 0.071476138 |
| HIST1H2BI;HIST1H2BK;HIST1H2BN;HIST1H2BD;HIST1H2BM;HIST1H2BH;HIST2H2BF;HIST1H2BC;HIST1H2BL;H2BFS | 0.087482689 | 0.584017758 |
| HIST1H2AC | -0.198186785 | 0.542101369 |
| OXCT;OXCT1 | 0.015308823 | 0.875841657 |
| GNB1 | 0.117475183 | 0.491157973 |
| HMGCS1 | -0.489707201 | 0.004550499 |
| SYBL1;VAMP7 | -0.341521058 | 0.028597854 |
| NUP155 | -0.330639963 | 0.013133555 |
| PAIP1 | -0.482442674 | 0.143470218 |
| AMPD2 | 0.159449803 | 0.22127266 |
| AHCYL1;FGFR2-AHCYL1;AHCYL2 | 0.335441382 | 0.0699963 |
| NNT | 0.142324608 | 0.392230855 |
| CSDE1;UNR | -0.086031455 | 0.481502035 |
| CAPZA1 | 0.150215801 | 0.155937847 |
| TRIM33 | -0.105772738 | 0.693414724 |
| PAF1 | -0.73529214 | 0.057010729 |
| WDR77 | -0.057802466 | 0.617758047 |
| HNRPUL1;HNRNPUL1 | -0.002972973 | 0.972623011 |
| PAFAH1B3 | -0.02935904 | 0.827487976 |
| TIMM50 | -0.007969911 | 0.907140215 |
| PPP1R13L | -0.097125838 | 0.396115427 |
| PPP5C | 0.020599691 | 0.893562708 |
| ARHGEF1 | -0.005832765 | 0.956196818 |
| SAE1 | 0.275523899 | 0.019570847 |
| SYMPK;SYMPK variant protein | -0.297697211 | 0.026008139 |
| NAPA | -0.12964779 | 0.45190529 |
| XRCC1 | 0.458758575 | 0.017351091 |
| VASP | 0.121471118 | 0.213984462 |
| PFKM | -0.135678061 | 0.403847577 |
| DDX23 | -0.0044769 | 0.976063633 |
| SMARCD1;SMARCD3 | 0.38485073 | 0.179023307 |
| PRKAG1;PRKAG2 | 0.115799437 | 0.333629301 |
| hCG_28765;AKAP2 | -0.140961138 | 0.335516093 |
| SMC2;SMC2L1 | 0.000612422 | 0.990972993 |
| HSDL2 | -0.195377158 | 0.159415464 |
| ROD1;PTBP3 | -0.390574029 | 0.123418424 |
| TEX10 | -0.172209846 | 0.093932667 |
| LTB4DH;PTGR1 | 0.922889739 | 0.010950795 |
| NCBP1 | -0.02085084 | 0.914280429 |
| UBE1;UBA1 | 0.216252669 | 0.039770625 |
| UBE2L3 | 0.045736268 | 0.632963374 |
| hCG_2011153;THOC5 | -0.051389526 | 0.625490196 |
| YWHAH | 0.053168435 | 0.587199408 |
| SF3A1 | -0.07917445 | 0.415538291 |
| MYH9 | 0.063577135 | 0.542027377 |
| XRCC6 | 0.127678353 | 0.2236404 |
| RAC2 | -0.451998453 | 0.007621162 |
| NAGA | -0.180721519 | 0.293118757 |
| RPL23 | 0.005311552 | 0.972142064 |
| SMARCE1 | 0.127974825 | 0.315279319 |
| LASP1 | 0.077212501 | 0.462301147 |
| CNP | -0.329003135 | 0.104513504 |
| ACLY;ACLY variant protein | -0.043260774 | 0.616833148 |
| RANGAP1 | -0.176214415 | 0.21809101 |
| PHF5A | 0.294048823 | 0.041657418 |
| RAB5C | -0.017914195 | 0.915279319 |
| RPL27 | -0.115157839 | 0.343692194 |
| FKBP10;DKFZp666D193 | -0.196423517 | 0.100517943 |
| VPS25 | -0.115321306 | 0.386200518 |
| hCG_16955;COA3 | -0.11902476 | 0.775064743 |
| VAT1 | 0.529289602 | 0.008805031 |
| PSMD13 | 0.100790054 | 0.315538291 |
| HEL-S-283;PSME3 | -0.095298362 | 0.373769885 |
| IFITM2;IFITM3;IFITM1 | 0.003116395 | 0.9745468 |
| PSAT1 | 0.729789008 | 0.00218276 |
| HNRPK;HNRNPK | -0.965895298 | 0.052793193 |
| TJP2 | 0.459823862 | 0.010358861 |
| RPL17;hCG_24487;RPL17-C18orf32 | -0.009120716 | 0.980836108 |
| CTSL1;CTSL | -0.064250946 | 0.348575657 |
| SPTLC1 | -0.272793733 | 0.272475028 |
| NEDD4L | 0.002115198 | 0.940251572 |
| KDSR;FVT1 | -0.322419705 | 0.012837588 |
| LMAN1 | 0.168290109 | 0.206252312 |
| SERPINB8 | 0.688126675 | 0.004217536 |
| VPS4B | -0.08872837 | 0.587865335 |
| ARL8B | 0.175982581 | 0.172290048 |
| ATG7 | -0.120773446 | 0.257713651 |
| THUMPD3 | 0.013809201 | 0.907103219 |
| FLJ00144;TMEM43 | 0.10260561 | 0.440251572 |
| RPL32 | -0.109247323 | 0.577284499 |
| TRNT1 | -0.178589539 | 0.4509064 |
| RAB5A | -0.299861596 | 0.094709582 |
| UBE2E1;UBE2E2;UBE2E3 | -0.609800852 | 0.103773585 |
| OXSR1 | 0.119244687 | 0.280503145 |
| RPSA;LOC388524 | -0.097935285 | 0.333703293 |
| EXOSC7 | -0.161050578 | 0.268146504 |
| RPL15 | -0.140823863 | 0.241805401 |
| MYL1;MYL3 | 0.250071767 | 0.250832408 |
| DHX30 | -0.133496164 | 0.218756937 |
| USP19 | 0.169317976 | 0.249759526 |
| APEH | -0.275748832 | 0.066666667 |
| PRKAR2A | 0.182657475 | 0.142064373 |
| GMPPB | -0.066274008 | 0.913244543 |
| GNL3 | -0.153637597 | 0.130965594 |
| CCDC72;TMA7;hCG_2014768 | 0.049390275 | 0.785164632 |
| RHOA;RHOC;ARHA | -0.334610888 | 0.029892712 |
| SUCLG2;DKFZp586M2023 | 0.266789953 | 0.025971143 |
| RPL29 | -0.189995907 | 0.242804292 |
| TMEM113;WDR82 | -0.061870533 | 0.605993341 |
| THEX1;ERI1 | 0.098974165 | 0.419977802 |
| CTSB | 0.019824449 | 0.910839808 |
| THUMPD1;44M2.1;DKFZp686C1054 | -0.079234886 | 0.457084721 |
| CHORDC1 | 0.017466859 | 0.946170921 |
| MRE11A | 0.009151198 | 0.952608213 |
| KDELC2 | -0.380265148 | 0.058675546 |
| DLAT | 0.169012667 | 0.18172401 |
| hCG_2032701;STT3A | 0.036707229 | 0.682759896 |
| ACBD3 | -0.172355763 | 0.155530892 |
| ARF1;ARF3 | -0.142453327 | 0.364446911 |
| LBR | -0.071243539 | 0.626378098 |
| PARP1 | 0.233667102 | 0.105697373 |
| DKFZp666D023;GUK1 | 0.433762469 | 0.150684425 |
| PPIL3 | -0.120841693 | 0.50099889 |
| DKFZp762L015;NIF3L1 | -0.844846716 | 0.214613393 |
| TSNAX;DISC1 | 0.078118362 | 0.615612283 |
| EEF1B2;LOC392793 | 0.20632972 | 0.106955235 |
| HSPD1 | -0.097923672 | 0.402145764 |
| SUMO1 | 0.221531901 | 0.142434332 |
| LANCL1 | 0.090320532 | 0.53936367 |
| BZW1;hCG_2022736 | 0.085055159 | 0.373288938 |
| CASP8;hCG_16983;Casp8 | -0.775907908 | 0.009507954 |
| AAMP | 0.008472927 | 0.973140954 |
| KIAA0971;FASTKD2 | -0.888067197 | 0.042619312 |
| DNPEP | -0.647761083 | 0.154421014 |
| AGFG1;HRB | -0.008456996 | 0.962042175 |
| MRPL44 | -0.046432192 | 0.810654828 |
| CUL3 | -0.292283371 | 0.046651868 |
| GMPPA | 0.196364444 | 0.193044765 |
| CAB39 | -0.355369627 | 0.785645579 |
| ACSL3 | -0.0319814 | 0.793932667 |
| NCL | 0.046132935 | 0.589900111 |
| TNRC15;GIGYF2 | 0.227386991 | 0.183462819 |
| NDUFA10 | 0.015746389 | 0.885238624 |
| POLR3C | -0.827981658 | 0.031372549 |
| TDP43;TARDBP | 0.066239215 | 0.488161302 |
| HDLBP | -0.18051535 | 0.152608213 |
| PEX11B | 0.003527913 | 0.922234554 |
| ENO1 | 0.033446326 | 0.720569737 |
| POLD1 | -0.858095806 | 0.002071772 |
| LRRC47 | -0.157136754 | 0.158564558 |
| PLOD1 | -0.39239359 | 0.070995191 |
| MDH2 | 0.16643742 | 0.105919349 |
| RPS9 | -0.033797593 | 0.699926008 |
| hCG_1640809;MYADM | -0.087858076 | 0.563522013 |
| RPS5 | -0.049880583 | 0.644321125 |
| CHMP2A | 0.24354368 | 0.033333333 |
| EPN1;EPN3 | -1.140481393 | 0.023529412 |
| UBE2M | 0.040416377 | 0.700776915 |
| TTLL12 | -0.097786811 | 0.395486496 |
| CYB5R3 | -0.154657552 | 0.372253052 |
| PACSIN2 | 0.09897262 | 0.368738439 |
| hCG_1989366;NUP50 | 0.049210063 | 0.636736959 |
| SSRP1 | 0.161165804 | 0.207436182 |
| DAK;TKFC | -0.058820031 | 0.647206807 |
| MTA2 | -0.137447204 | 0.212652608 |
| UBXN1;LOC51035 | -0.015605624 | 0.91690714 |
| TRMT112;HSPC152 | -1.043081546 | 0.162153163 |
| EHD1 | -0.110729256 | 0.427894932 |
| MRPL49 | -0.056222825 | 0.579060303 |
| CAPN1 | -0.162474697 | 0.181834998 |
| SF1 | -0.109524026 | 0.383536811 |
| RNASEH2C;AYP1 | -0.283924711 | 0.043174251 |
| RTN3 | 0.464866857 | 0.116611173 |
| PC | -0.12489386 | 0.279874214 |
| CPT1A | 0.727887677 | 0.002108768 |
| DHCR7 | -0.095092029 | 0.389493156 |
| BANF1 | -0.702536872 | 0.098187199 |
| RAB6A | 0.045903163 | 0.70972993 |
| CORO1B;DKFZp762I166 | 0.09701231 | 0.276988531 |
| NDUFS8 | -0.130151464 | 0.306511284 |
| SERPINH1 | -0.188681047 | 0.104846467 |
| PICALM | -0.020290498 | 0.908768036 |
| WDR71;PAAF1 | 0.166935267 | 0.513762486 |
| hCG_2003792;HYPK | 0.484202963 | 0.145837958 |
| ADSS | -0.49506002 | 0.007251202 |
| TFB2M | -0.543859511 | 0.018386977 |
| EIF3S1;EIF3J | 0.141121951 | 0.225527192 |
| NEDD4 | 0.382429424 | 0.008953015 |
| C15orf48;NMES1 | 0.046880301 | 0.624047355 |
| C15orf15;RSL24D1 | 0.060671737 | 0.590048095 |
| hCG_2001986;SQRDL | 0.006538598 | 0.858416574 |
| CLPX;DKFZp586J151 | 0.097185569 | 0.401627821 |
| HEL-S-270;ANXA2;ANXA2P2 | -0.007072532 | 0.9236404 |
| RPLP1 | -0.158876907 | 0.38290788 |
| PPP4C | -0.064792093 | 0.543877174 |
| AGPAT5 | 0.314714167 | 0.225823159 |
| TIMM9 | 0.023199617 | 0.924417314 |
| GNPNAT1 | 0.041083281 | 0.811061783 |
| MTHFD1 | -0.142368016 | 0.127561968 |
| KTN1 | 0.236640348 | 0.03100259 |
| NUMB | 0.241561759 | 0.518571957 |
| FERMT2;PLEKHC1 | -0.080275925 | 0.394894562 |
| LGALS3;hCG_22119 | 0.043959409 | 0.757528672 |
| ACTN1 | -0.377249797 | 0.019311876 |
| NPC2 | 0.458058721 | 0.01054384 |
| DLST | 0.257332678 | 0.069589345 |
| ERH | -0.203281606 | 0.244432112 |
| SFRS5;SRSF5 | 0.106648274 | 0.356603774 |
| ALDH6A1;MMSADHA | -0.044540497 | 0.799556049 |
| CPSF2 | -0.428059769 | 0.161783204 |
| TMED10 | 0.085992323 | 0.592008879 |
| SERPINA1 | -1.330308235 | 0.47317795 |
| CCNK | -0.903141961 | 0.010876804 |
| SETD3 | 0.063280967 | 0.60118387 |
| WARS | 0.145128841 | 0.165741768 |
| KIAA2010;SMEK1;SMEK2 | -0.17312347 | 0.327561968 |
| PAPOLA | 0.04012712 | 0.745467999 |
| CDC42BPB | -0.224743893 | 0.342841287 |
| MTA1 | -0.075379739 | 0.506659267 |
| EIF5 | -0.022147084 | 0.817388087 |
| SHCBP1 | -0.096930037 | 0.633777284 |
| DNAJA2 | 0.04775397 | 0.762375139 |
| FAM192A;NIP30 | 0.13857093 | 0.387384388 |
| GOT2 | -0.037887243 | 0.758860525 |
| NUDT21;DKFZp313O211 | 0.00984119 | 0.930410655 |
| CBFB | -0.33736361 | 0.264964854 |
| NUTF2 | -0.038416838 | 0.821124676 |
| DYNC1LI2;DKFZp686J08252 | 0.127293343 | 0.386533481 |
| VPS4A;DKFZp434E0418 | 0.153527713 | 0.280281169 |
| DLD | 0.152484408 | 0.166222716 |
| PBEF1;NAMPT | -0.206576865 | 0.483573807 |
| NDUFA5 | -0.241555435 | 0.098113208 |
| RBM28 | -0.017491139 | 0.857750647 |
| CAV1 | 0.06954827 | 0.632667407 |
| TNPO3 | 0.368237091 | 0.040880503 |
| AKR1B1 | 0.066702878 | 0.592415834 |
| UBL5 | 0.285984123 | 0.158823529 |
| CDC37 | 0.02923969 | 0.770551239 |
| MRPL4 | -0.239055493 | 0.108287088 |
| ILF3 | 0.022445343 | 0.825749168 |
| DHPS | -0.942994639 | 0.051165372 |
| TMEM205;UNQ501 | 0.185944634 | 0.182352941 |
| LDLR | -0.878180745 | 0.02445431 |
| BTBD14B;NACC1;BTBD14A;NACC2 | 0.084732845 | 0.515797262 |
| DNMT1 | -0.252476497 | 0.048390677 |
| PRKCSH | 0.272001487 | 0.032963374 |
| TNPO2;TNPO2 variant protein | 0.078155799 | 0.564298927 |
| RAB3D | -0.72257432 | 0.139881613 |
| RAD23A | -0.023356018 | 0.832112468 |
| RAB8A | -0.023287806 | 0.89700333 |
| TRMT1 | -0.228728004 | 0.07136515 |
| RAB3A;RAB3C | -5.966572392 | 7.40E-05 |
| PRKACA;KIN27;PRKACB | 0.029640952 | 0.927672956 |
| GATAD2A | -0.016527319 | 0.907288198 |
| AP1M1 | 0.342234728 | 0.031557529 |
| UPF1 | 0.26630397 | 0.028745838 |
| MVB12A;LOC93343 | -0.144767814 | 0.154716981 |
| TMED9 | 0.337481812 | 0.033037366 |
| LOC115098;CCDC124 | 0.185384734 | 0.174509804 |
| HNRPF;HNRNPF | -0.363397404 | 0.015020348 |
| MCM4 | -0.112126205 | 0.294413615 |
| ATP6V1H | 0.635285357 | 0.061006289 |
| RAB2;RAB2A | -0.246792011 | 0.057565668 |
| COPS5 | 0.055278463 | 0.646022937 |
| TCEB1 | -0.121918459 | 0.355049945 |
| SDCBP | 0.379337183 | 0.035294118 |
| LACTB2 | 0.152259817 | 0.298039216 |
| NUDT1 | 0.083027402 | 0.578320385 |
| EIF3B;EIF3S9 | 0.014908542 | 0.866111728 |
| POLE3 | -1.035273666 | 0.010062893 |
| TRIM32 | -2.011454508 | 0.000739919 |
| RAB14 | -0.22385865 | 0.044580096 |
| MRRF | -0.037778817 | 0.743100259 |
| FBXL18 | -0.151574771 | 0.168701443 |
| PPP6C | -0.34668961 | 0.018719941 |
| GAPVD1 | 0.084492999 | 0.560784314 |
| C9orf88;FAM129B | -0.181730794 | 0.130447651 |
| CDK9 | 0.154295161 | 0.2681835 |
| STOM | 0.276640007 | 0.05308916 |
| ATP6V1G1 | 0.400199968 | 0.013651498 |
| SET;SETSIP | -0.079153454 | 0.450795413 |
| ARPC5L | -0.622090566 | 0.224787273 |
| hCG_31253;FUBP3 | 0.070962913 | 0.477062523 |
| NUP214;DKFZp686J0330 | 0.011640622 | 0.88390677 |
| MRPS2 | -0.175176498 | 0.209803922 |
| hCG_30600;PTGES | -0.461941119 | 0.095671476 |
| C9orf32;NTMT1 | -0.265938065 | 0.06163522 |
| MRPS7 | -0.132603067 | 0.235072142 |
| WBP2 | 0.565549941 | 0.005438402 |
| PRPSAP1 | 0.065975216 | 0.630262671 |
| TK1 | 0.023349522 | 0.895190529 |
| RPL38 | -0.490587723 | 0.014206437 |
| NPLOC4 | -0.115356795 | 0.381132075 |
| SUMO2 | -0.302320322 | 0.067924528 |
| P4HB | 0.026257369 | 0.750758417 |
| SYNGR2 | 0.80867614 | 0.066111728 |
| SLC16A3 | -0.146966414 | 0.408435072 |
| SRSF2;SFRS2 | 0.000691761 | 0.988420274 |
| PYCR1 | 0.303345421 | 0.095375509 |
| 9-Sep | 0.279252326 | 0.06936737 |
| DDX48;EIF4A3 | -0.123602418 | 0.255900851 |
| POU2F1;POU2F2;POU2F3 | -0.087963539 | 0.740473548 |
| MGST3 | -0.26278134 | 0.44335923 |
| DARS2 | -0.3101559 | 0.0172771 |
| CACYBP | -0.016933646 | 0.866074732 |
| UCK2 | -0.256614401 | 0.072918979 |
| TOR1AIP2 | -0.706230104 | 0.060377358 |
| NEK7 | 0.087748921 | 0.403921569 |
| ATP2B4;DKFZp686M088 | 0.055023506 | 0.563300037 |
| C1orf24;FAM129A | 0.061413155 | 0.628671846 |
| TROVE2 | 0.03054628 | 0.797262301 |
| CPNE3 | -0.393735532 | 0.025564188 |
| DDX3Y | 0.16949612 | 0.179763226 |
| UBE2T | -0.971594855 | 0.001886792 |
| RBBP5 | -0.0300306 | 0.814539401 |
| COX6C | 0.358073536 | 0.008879023 |
| PPM2C;PDP1 | -0.023906657 | 0.821605623 |
| PABPC1 | -0.054674591 | 0.643063263 |
| MTDH | -0.045122062 | 0.739992601 |
| RPL30 | -0.12067602 | 0.287754347 |
| DECR1;DECR | 0.123234116 | 0.304143544 |
| ENY2 | 0.547406415 | 0.106030337 |
| FAM49B;DKFZp686B04128;BM009 | 0.090502802 | 0.529263781 |
| ATAD2 | -1.162977122 | 0.005919349 |
| ATP6V1C1 | 0.619854614 | 0.00418054 |
| RAD21 | -0.013538463 | 0.873399926 |
| EMC7;C15orf24 | 0.111514425 | 0.416611173 |
| AQR | -0.207149604 | 0.1236404 |
| CHP1;CHP | -0.116375185 | 0.481058084 |
| EHD4 | -0.210704127 | 0.107251202 |
| FAM82C;RMDN3 | 0.304381987 | 0.292933777 |
| RBPJ;RBPSUH | -1.227459081 | 0.009877913 |
| VPS18 | 0.337295744 | 0.05963744 |
| OCIAD1 | 0.379463105 | 0.022493526 |
| HUWE1 | 0.014209999 | 0.857935627 |
| LOC92689;FAM114A1 | -0.478557462 | 0.55227525 |
| SHINC3;TMEM33 | -0.032526965 | 0.77872734 |
| GNPDA2 | 0.071631753 | 0.652682205 |
| MAGED2;MAGED1 | -0.33292468 | 0.179837218 |
| NDUFA4 | -0.738409093 | 0.004254532 |
| BZW2 | -0.686914752 | 0.060044395 |
| ANLN | 0.275988271 | 0.14372919 |
| PSMA2 | 0.105458003 | 0.489604144 |
| DFNA5 | -0.055842405 | 0.749611543 |
| HIBADH | -0.10064497 | 0.622900481 |
| LUZP1 | 0.977061524 | 0.00399556 |
| RCC2 | 0.033083935 | 0.766259711 |
| DDOST | 0.047770578 | 0.695190529 |
| STEAP3 | -0.461555074 | 0.037624861 |
| C1orf33;MRTO4 | -0.127579102 | 0.190566038 |
| CDC42;hCG_39634 | 0.170647994 | 0.137106918 |
| DDX18 | 0.006627506 | 0.936145024 |
| ACTR3 | 0.066867505 | 0.515501295 |
| MRPS27 | -0.236126227 | 0.202219756 |
| HEXB | 0.341998084 | 0.061672216 |
| TNPO1 | -0.008803546 | 0.96063633 |
| GLRX | 0.697600887 | 0.021568627 |
| MAP1B;DKFZp686F1345 | 0.405064343 | 0.017943026 |
| DYR;DHFR;DHFRL1 | 0.046057238 | 0.676803552 |
| HIST1H2AH;H2AFJ;HIST1H2AK;HIST1H2AJ;HIST2H2AC;HIST2H2AA3;HIST1H2AG | -0.288767704 | 0.051942286 |
| hCG_27698;DDX47;E4-DBP | -0.403701081 | 0.006770255 |
| HEBP1 | 0.17732716 | 0.271661117 |
| RECQL | 0.121158259 | 0.299112098 |
| WBP11 | -0.065205802 | 0.637587865 |
| MGST1 | -0.140010981 | 0.277136515 |
| KRT18 | -1.087687369 | 0.006104329 |
| PLEKHA5 | 0.189084432 | 0.124898261 |
| HNRNPA1;HNRPA1;hCG_2020860;RP11-78J21.1;HNRNPA1L2 | -0.282533394 | 0.047354791 |
| ITGA5 | -0.433692022 | 0.024972253 |
| PPFIBP1 | -0.041605562 | 0.768664447 |
| RPS26;RPS26P11 | -0.095204393 | 0.527857936 |
| FAM62A;ESYT1 | -0.159712992 | 0.243026267 |
| SMARCC2 | -0.081163396 | 0.484461709 |
| PTGES3 | -0.116929669 | 0.490418054 |
| NACA;hCG_2016482 | -0.333882401 | 0.009618942 |
| FAM112B;GTSF1 | -0.262204762 | 0.192711802 |
| METTL1 | -0.192956036 | 0.334591195 |
| COPZ1 | -0.143013358 | 0.265778764 |
| CS | 0.140605483 | 0.2127266 |
| CDK2 | -0.169372497 | 0.277802442 |
| PA2G4 | -0.003773976 | 0.978283389 |
| RAP1B;RAP1A | 0.19449402 | 0.083684795 |
| SHMT2;HEL-S-51e;DKFZp686P09201 | -0.295278914 | 0.020347762 |
| RAB21 | -0.017005301 | 0.916389197 |
| CDK4 | -0.4430184 | 0.061524232 |
| NAP1L1 | 0.129872897 | 0.216019238 |
| OSBPL8;DKFZp686C0249 | 0.010038819 | 0.92963374 |
| DKFZp686E12166;GNS | 0.097340888 | 0.479467259 |
| NEDD1 | -0.143245381 | 0.467924528 |
| TMPO | 0.356048812 | 0.060007399 |
| SLC25A3 | -0.068491381 | 0.589382168 |
| CKAP4 | -0.105185405 | 0.341176471 |
| PWP1 | 0.873174465 | 0.240769515 |
| CORO1C | 0.206614714 | 0.078616352 |
| GLTP | 0.207950064 | 0.159896411 |
| ANAPC7 | -0.803384695 | 0.028116907 |
| RPL6 | -0.026419479 | 0.827376989 |
| TXNRD1 | 1.48074449 | 0.00081391 |
| CLIP1;RSN | 0.371067333 | 0.012615612 |
| DENR | 1.148008213 | 0.126045135 |
| RPLP0;RPLP0P6 | -0.036677109 | 0.715945246 |
| DIABLO | 0.195382197 | 0.139733629 |
| GCTG | -0.498352567 | 0.009123196 |
| PIR | 1.249612056 | 0.001923788 |
| PDHA1;PDHA1/LOC79064 | -0.009620089 | 0.906474288 |
| DKFZp779I1858;HCCS | -0.657580567 | 0.059489456 |
| CTPS2 | -0.170406182 | 0.087976323 |
| MAGEA4 | 0.064487747 | 0.491971883 |
| MAGEA2B;MAGEA6;hCG_1739882;MAGEA2 | -0.560790712 | 0.126526082 |
| P15RS;RPRD1A | -0.225883172 | 0.147539771 |
| ANPEP | -0.773953766 | 0.002848687 |
| hCG_1991735;IQGAP1 | 0.027512101 | 0.781761006 |
| RNH1 | -0.014657776 | 0.885793563 |
| ALDH1A3;DKFZp686G1675 | -0.607437508 | 0.006807251 |
| RPLP2 | -0.17417359 | 0.212097669 |
| MRPL23 | 0.356660211 | 0.207843137 |
| CD151 | -0.007324015 | 0.963152053 |
| NAP1L4 | -0.110407304 | 0.30418054 |
| ARMCX3 | 0.477915232 | 0.008287088 |
| CARS | 0.020808808 | 0.834443211 |
| HIST1H2BJ;HIST2H2BE;HIST1H2BB;HIST1H2BO;HIST3H2BB;HIST2H2BD;HIST2H2BC | -0.166727795 | 0.422382538 |
| hCG_2005638;DDX39B | -0.026112175 | 0.801257862 |
| VARS | -0.051730704 | 0.623381428 |
| GNL1;HSR1 | 0.093240092 | 0.519755827 |
| BRD2 | -0.193936494 | 0.329596744 |
| BAT3;BAG6 | 0.037732443 | 0.70418054 |
| DAXX | -0.711061936 | 0.028264891 |
| KIFC1 | 0.199032055 | 0.241398446 |
| TAPBP | -0.140468772 | 0.392304846 |
| PIG60;MTCH1 | -0.322315173 | 0.125194229 |
| PPIL1 | 0.221075346 | 0.185164632 |
| LEMD2 | 0.047704251 | 0.692859785 |
| PPP2R5D | 0.028314756 | 0.790862005 |
| RPL7L1 | 0.071172852 | 0.580429153 |
| HSP90AB1 | -0.045203439 | 0.757084721 |
| MUT | -0.187970346 | 0.24672586 |
| PAICS | -0.263600035 | 0.014391417 |
| GRSF1 | -0.292270682 | 0.031446541 |
| EXOC1 | -6.69E-05 | 0.971143174 |
| UBE1L2;UBA6 | -0.169952927 | 0.160081391 |
| G3BP2 | 0.23651518 | 0.095301517 |
| SEC31L1;SEC31A | 0.099172356 | 0.334110248 |
| PDLIM5 | 0.135799353 | 0.237550869 |
| HNRPD;HNRNPD | -0.168923005 | 0.1127266 |
| VDP;USO1 | 0.270104146 | 0.050721421 |
| SCARB2 | 0.622118504 | 0.003773585 |
| UBE2D3;UBE2D2 | 0.207341607 | 0.072216056 |
| RPL34 | 0.210263296 | 0.161154273 |
| DC2;OSTC;OSTCL | -0.039917837 | 0.680059193 |
| CCT6A | -0.054780277 | 0.610691824 |
| PSPH | 0.64208369 | 0.004994451 |
| ASL | 0.173746307 | 0.46618572 |
| PSPC1 | -0.062101793 | 0.422826489 |
| HSPH1 | 0.089688234 | 0.449981502 |
| RFC3 | -0.472911695 | 0.018238994 |
| SLC7A1 | -0.135264597 | 0.24872364 |
| HMGB1;hCG_1991922;HMGB1P1 | 0.248649567 | 0.2190899 |
| HEL-S-37;LCP1 | -0.285482027 | 0.023825379 |
| GTF2F2 | 0.061338293 | 0.703255642 |
| RB1 | 0.060954918 | 0.635553089 |
| TSC22D1;TSC22D2 | -1.306840263 | 0.013540511 |
| SPG20 | -0.313480361 | 0.035331114 |
| RANBP5;IPO5 | -0.218292519 | 0.041805401 |
| LAMP1 | 0.643843631 | 0.003958565 |
| MED4 | 0.019267674 | 0.927229005 |
| ARHGEF7;KIAA0142 | 0.610158007 | 0.003810581 |
| RP11-98F14.6;PCID2 | -0.050522792 | 0.734480207 |
| GBA | 0.800495636 | 0.004032556 |
| FHOD1 | 0.056632449 | 0.689863115 |
| SRGAP2 | 0.372348692 | 0.033703293 |
| GPX4 | -0.299710726 | 0.0127266 |
| DKFZp586J0119;EIF2B4 | 0.093106448 | 0.480059193 |
| NDUFS7 | -0.0646319 | 0.645467999 |
| KHSRP | 0.150516428 | 0.117758047 |
| UHRF1 | -0.621536337 | 0.009803922 |
| MAGT1 | 1.012407392 | 0.027746948 |
| POLA1 | -0.688079703 | 0.014428413 |
| CTNNBL1 | -0.00708945 | 0.888753237 |
| SPCS2 | 0.075586095 | 0.472216056 |
| FGF2 | -0.197654457 | 0.491379948 |
| SLIRP | -0.101610827 | 0.485645579 |
| GPX1 | -0.573279661 | 0.005660377 |
| RPS24 | -0.250993763 | 0.245394007 |
| MYEF2 | 0.116159826 | 0.384202738 |
| EIF5B | 0.409134759 | 0.027968923 |
| MYCBP | -0.176950852 | 0.117425083 |
| SMAP2 | 0.151754097 | 0.474990751 |
| SELT | -0.285494074 | 0.032704403 |
| TAF9;AK6 | 0.339745938 | 0.022604514 |
| COG1 | -0.59643367 | 0.013318535 |
| MTHFD1L | -0.07007182 | 0.528560858 |
| CLTC | 0.196815575 | 0.073621902 |
| POLR2E | -0.28616538 | 0.055974843 |
| SH3GLB1 | 0.18049027 | 0.080762116 |
| TRAPPC3 | 1.894023426 | 0.004143544 |
| GMPR2 | 0.082410693 | 0.567036626 |
| RBM15 | 0.437302839 | 0.045615982 |
| THOC1 | -0.017251288 | 0.903625601 |
| SCAMP1 | 0.104125683 | 0.345874954 |
| ASNA1 | 0.13878244 | 0.285164632 |
| ESYT2 | -0.178120529 | 0.29463559 |
| VTA1 | -0.045251364 | 0.782574917 |
| AP2M1 | 0.073112333 | 0.445504994 |
| FAM21A;FAM21C | 0.22412331 | 0.251720311 |
| DHX29 | -0.123330382 | 0.641287458 |
| SRP19 | -0.130573876 | 0.268590455 |
| TPD52L2 | 0.213546408 | 0.044839068 |
| UGP2 | -0.002209255 | 0.989752127 |
| RAVER1 | -0.287891246 | 0.079134295 |
| IDH3B | 0.094217322 | 0.47473178 |
| BRCC3 | 0.275752665 | 0.058712542 |
| METTL7B | -0.055402529 | 0.874953755 |
| SBDS | 0.828675691 | 0.003551609 |
| HYOU1 | 0.122738405 | 0.258120607 |
| RBM26 | 0.006985171 | 0.970033296 |
| TJP1;DKFZp686A1195 | 0.399830571 | 0.025009249 |
| YTHDF3;DKFZp451J085;DKFZp451A052 | 0.472997892 | 0.011505734 |
| ACOT1;ACOT2 | 0.160304019 | 0.180503145 |
| DHRS7;DKFZp564H1664 | 0.370385374 | 0.027487976 |
| 15-Sep | -0.372536518 | 0.038586755 |
| ACAP2 | 0.180651045 | 0.122752497 |
| LYPLA1 | -0.171626489 | 0.196670366 |
| RPP40 | -0.326558973 | 0.058194599 |
| PSME2 | -0.048027712 | 0.733148354 |
| KIAA1524 | -0.949809974 | 0.001590825 |
| KIAA1033 | -0.02164358 | 0.849537551 |
| BSG;hEMMPRIN | -0.189708341 | 0.096781354 |
| MRPL45 | 0.658883106 | 0.065667777 |
| HECTD1 | -0.155959174 | 0.35645579 |
| PSMC6;HEL-S-73 | -0.009927439 | 0.91490936 |
| SCRN1 | 0.011439397 | 0.956492786 |
| CUL1 | 0.108049045 | 0.432852386 |
| GGCT | -0.373384772 | 0.004768775 |
| CDK5 | -0.289448118 | 0.226563078 |
| ABCF2 | -0.252204702 | 0.06899741 |
| GARS | 0.532036221 | 0.004698483 |
| ERP70;PDIA4 | -0.043654179 | 0.675952645 |
| RHEB | 0.139625641 | 0.30199778 |
| RNF114 | 0.270059821 | 0.982241953 |
| C21orf33 | -0.109090441 | 0.536773955 |
| PYCRL | 0.12050149 | 0.409433962 |
| WDR18 | -0.256276484 | 0.074250832 |
| SCYL1 | -0.157179292 | 0.092378838 |
| MBNL1;MBLL;MBNL2 | 0.022378617 | 0.815649279 |
| GSTM4;GSTM1 | -0.682341436 | 0.046133925 |
| APP | -0.325735689 | 0.541028487 |
| SNX6 | -0.201561847 | 0.252793193 |
| PCMT1 | 0.194870249 | 0.172918979 |
| MYO6 | 0.19476229 | 0.832260451 |
| NOLC1 | -0.023899986 | 0.879319275 |
| PRPF38B | -0.071453321 | 0.898039216 |
| CCBL2;hCG_23341 | 0.234449654 | 0.25009249 |
| SNRPC | -0.13916555 | 0.181982982 |
| ABI1 | 0.375376967 | 0.013614502 |
| SFXN3 | -0.326855936 | 0.064779874 |
| EIF4G3 | -0.297253116 | 0.109618942 |
| EXOC7;DKFZp686P1551 | -0.030429073 | 0.80754717 |
| HADH | -0.094736136 | 0.451646319 |
| FHL2;AAG11 | 0.738071084 | 0.003107658 |
| CHD1L | 0.020045765 | 0.84890862 |
| PHC2;HPH2 | -0.03589669 | 0.693118757 |
| TOR1AIP1 | -0.145750418 | 0.224158343 |
| PITPNB | -0.148213105 | 0.246059933 |
| SMARCA4 | 0.260006109 | 0.036884943 |
| IVD | 0.21378773 | 0.104328524 |
| WDR36 | -0.07852148 | 0.515242323 |
| HEL-S-28;ILK | -0.337920591 | 0.068664447 |
| BTAF1 | -0.125257325 | 0.467443581 |
| NCEH1 | -0.385088212 | 0.017832038 |
| CUL2 | 0.016649675 | 0.929115797 |
| C14orf2;MP68 | -0.152244666 | 0.359785424 |
| OGFOD1 | 0.093817248 | 0.446836848 |
| RAB3B | -1.649191178 | 0.000887902 |
| TMED7-TICAM2;TMED7 | 0.053568336 | 0.616167222 |
| PPAN-P2RY11;PPAN | 0.446822047 | 0.019533851 |
| CORO7-PAM16;CORO7;PAM16;hCG_1787779 | 0.019764568 | 0.845467999 |
| ERAP1;ARTS-1 | -0.334640619 | 0.031520533 |
| NPC1 | 0.602913076 | 0.005845357 |
| HELLS | -1.07242147 | 0.001368849 |
| NAA15;NARG1 | 0.125492972 | 0.156122826 |
| TUBB3 | -0.050765031 | 0.642878283 |
| RPS15 | -0.266986683 | 0.180133185 |
| PWP2 | -0.223876653 | 0.096929338 |
| CYP51A1 | -1.449041034 | 0.000517943 |
| SYNE1 | 0.109018322 | 0.810173881 |
| CAST | 0.271314419 | 0.025120237 |
| CHD4 | -0.1360466 | 0.201812801 |
| CAPNS1 | -0.22871463 | 0.074916759 |
| NDUFA6 | 0.561614834 | 0.124195339 |
| MAP4K4;HEL-S-31;TNIK;MINK1 | -0.114262195 | 0.941990381 |
| DDX3X | -0.094483882 | 0.414280429 |
| STXBP1 | 1.2966548 | 0.007214206 |
| TATDN1 | 0.367689269 | 0.016611173 |
| PHF6 | 0.070084662 | 0.721124676 |
| FXR1 | 0.159132653 | 0.216537181 |
| LRRFIP1 | 0.442973493 | 0.022308546 |
| DIAPH1 | 0.002842398 | 0.96145024 |
| HEL-S-103;HSPA1B;HSPA1A | 1.235334778 | 0.000924898 |
| SCRIB | -0.526734234 | 0.003699593 |
| RBFOX2;RBFOX1;DKFZp547L059;A2BP1 | -0.669004615 | 0.044395117 |
| CTDP1 | -0.199973184 | 0.184720681 |
| DNAJB6 | -0.268202687 | 0.066851646 |
| ACO2 | 0.478046831 | 0.004920459 |
| TPM1 | -0.036593986 | 0.790566038 |
| PSMA7;hCG_41772 | 0.243911772 | 0.051461339 |
| PGLS | 0.02330384 | 0.813836478 |
| PSME1 | -0.340863957 | 0.058786533 |
| TPT1 | -0.069217307 | 0.506696263 |
| MATR3;DKFZp686K23100 | -0.101091527 | 0.293155753 |
|  | 0.43298225 | 0.122789493 |
| JMJD6 | 0.269079804 | 0.174139845 |
| ARID1A;ARID1A variant protein | 0.351712816 | 0.013096559 |
| ANXA6 | -0.311231231 | 0.02145764 |
| ACADM;DKFZp686M24262 | 0.029945976 | 0.851424343 |
| ACTN4 | -0.170667047 | 0.093192749 |
| CLPTM1 | -0.017330849 | 0.922012579 |
| RCC1 | 0.037991638 | 0.753385128 |
| ETFA | 0.238185052 | 0.101775805 |
| ATP2A2 | -0.107980651 | 0.266333703 |
| ACADSB | -0.344366904 | 0.143914169 |
| CRK | 0.127765314 | 0.293747688 |
| LAMB3 | 0.565804756 | 0.15427303 |
| HEXA | 0.374807786 | 0.167591565 |
| GDI1 | 0.264140662 | 0.070440252 |
| LGALS3BP | 0.936355957 | 0.027339993 |
| HSD17B10 | -0.15377433 | 0.211616722 |
| PCK2 | 0.647941579 | 0.004402516 |
| PSMD12 | -0.080873194 | 0.577654458 |
| NPM1 | -0.009638459 | 0.968960414 |
| RBMS1 | 0.713837787 | 0.077358491 |
| MCM7 | -0.129161775 | 0.162745098 |
| PPP3CA;PPP3CB;PPP3CC | 0.045993331 | 0.816648169 |
| TPM3;DKFZp686J1372 | 0.045234927 | 0.682685905 |
| HSD17B4 | -0.0181569 | 0.85127636 |
| PMM2 | 0.132314843 | 0.265223825 |
| CNBP | 0.112019741 | 0.539585646 |
| HGS | 0.143042904 | 0.181613023 |
| YARS | 0.149399748 | 0.214354421 |
| MCM3;HCC5 | 0.00064316 | 0.998039216 |
| SRRM1 | -0.088945337 | 0.500221976 |
| DKFZp686E2459;RBM10 | 0.229288825 | 0.111135775 |
| MPDU1;HBEBP2BPA | -0.282484838 | 0.963928968 |
| GFPT2 | -0.592581536 | 0.003440622 |
| CLINT1 | -0.013476313 | 0.872844987 |
| DPM1 | 0.588643908 | 0.06381798 |
| RBM14 | 0.121398282 | 0.324972253 |
| RNF40 | 0.285630156 | 0.204587495 |
| NONO | 0.014237817 | 0.929485757 |
| SRSF10 | -0.390337434 | 0.019052904 |
| SURF1 | -0.220372774 | 0.245209027 |
| SLC39A14 | -0.142398944 | 0.289234184 |
| PQBP1 | -0.105432158 | 0.290825009 |
| MRPS28 | -0.203602905 | 0.130225675 |
| DAZAP1;DAZAP1/MEF2D fusion | -0.254040958 | 0.034554199 |
| ETHE1 | -0.465622333 | 0.0236404 |
| RDH11 | -0.171848128 | 0.208583056 |
| LSM1 | -0.036366863 | 0.879060303 |
| FOSL1 | 0.686103239 | 0.03836478 |
| TAP1;TAP1*0102N | -0.21349775 | 0.062190159 |
| DCTN4 | 0.135672654 | 0.252645209 |
| PAK1IP1 | 0.319748655 | 0.027007029 |
| TBL2 | 0.499101029 | 0.011690714 |
| VPS28 | 0.816609928 | 0.175952645 |
| COPS4 | -0.106469267 | 0.316870144 |
| AP3B1 | -0.154007407 | 0.242656308 |
| HEL162;STRBP | 0.721668582 | 0.095190529 |
| ABCF3 | -0.021860897 | 0.804698483 |
| SEC63 | 0.065626445 | 0.596781354 |
| ELAC2 | -0.685589645 | 0.007103219 |
| MCMBP | -0.401732615 | 0.007732149 |
| SUMF2;DKFZP566I1024 | 0.085296377 | 0.670551239 |
| HNRNPLL;HNRPLL | -0.227804553 | 0.125157233 |
| PYCR2;P5CR2 | -0.080653653 | 0.401368849 |
| HM13 | 0.163457391 | 0.14354421 |
| DNAJC19 | 0.305813306 | 0.057380688 |
| VKORC1 | 0.387415228 | 0.191009989 |
| PROCR | -0.189985901 | 0.337402886 |
| SCYL2 | 0.204663335 | 0.091009989 |
| GATAD2B | 0.092914486 | 0.485349612 |
| PSMA5 | 0.184204066 | 0.106844247 |
| PSMB8;PSM8 | -0.369308965 | 0.042323344 |
| FLOT1 | -0.212644355 | 0.057491676 |
| LYPLA2 | 0.093055561 | 0.433666297 |
| LRWD1 | -0.053344931 | 0.68290788 |
| ADK | -0.140279782 | 0.361339253 |
| METAP2 | 0.001805786 | 0.841990381 |
| AP1G1 | 0.226578505 | 0.080207177 |
| AP2B1;DKFZp781K0743 | 0.116267843 | 0.239992601 |
| BUB3 | 0.001863083 | 0.986903441 |
| BLVRA | 0.075998053 | 0.564261931 |
| COMT | -0.039655161 | 0.674398816 |
| DUSP23 | 0.316769993 | 0.11790603 |
| ESD | 0.127414105 | 0.221420644 |
| GLRX3 | 0.117730929 | 0.267147614 |
| GPD2 | -0.138221512 | 0.245320015 |
| GMPS | -0.14686954 | 0.145246023 |
| HIBCH | -0.9756093 | 0.140769515 |
| HPRT1 | -0.04103444 | 0.67236404 |
| ITGA3 | -0.143043723 | 0.291416944 |
| LRRC40 | -0.557410998 | 0.023048465 |
| PLIN3 | -0.090383524 | 0.540732519 |
| MAT2B | -0.02822942 | 0.831964484 |
| MAT2A | -0.055239253 | 0.559230485 |
| OAT | -0.74488523 | 0.002441731 |
| OSBPL11 | -0.158177435 | 0.34236034 |
| PXN | -0.14086351 | 0.301405845 |
| PEPD | 0.542900104 | 0.005512394 |
| PSMC5 | 0.033236636 | 0.8236404 |
| PSMB2 | 0.264677366 | 0.041694414 |
| PPP2R2A | 0.096224988 | 0.39563448 |
| PSMF1 | -1.148539135 | 0.013836478 |
| DARS | 0.027233962 | 0.740436552 |
| ACAT1 | 0.01407544 | 0.922752497 |
| MPST | -0.206996713 | 0.341250462 |
| TXNDC17 | 0.026280165 | 0.815871254 |
| USP5 | -0.083573399 | 0.576137625 |
| UCHL3 | 0.057088106 | 0.641065483 |
| CPT2 | -0.154931887 | 0.283203848 |
| LTA4H | 0.029991955 | 0.737809841 |
| MCCC2 | -0.502222747 | 0.119348872 |
| PPME1 | -0.097527336 | 0.572660007 |
| PSMA3 | 0.18206655 | 0.219163892 |
| PSMA6 | 0.123930794 | 0.297706252 |
| PSMB1 | 0.110244947 | 0.311653718 |
| PSMB4 | -0.030567492 | 0.762301147 |
| SND1 | -0.028054906 | 0.728338883 |
| SRRM2;KIAA0324 | 0.071448294 | 0.514317425 |
| STAMBP | 0.34915969 | 0.031668516 |
| TALDO1 | 0.053568862 | 0.559378468 |
| ATP6V1B2 | 0.289835977 | 0.010654828 |
| GOT1;GIG18 | 0.751438454 | 0.00199778 |
| PSMC2 | 0.089330833 | 0.367702553 |
| HSPA13 | 0.67571534 | 0.003144654 |
| AK2 | -0.316349608 | 0.022789493 |
| RANBP1 | -0.14393894 | 0.203477617 |
| CALD1 | -0.779690894 | 0.002330744 |
| ST13;ST13P5;ST13P4 | 0.050569402 | 0.580392157 |
| UAP1 | 0.030994009 | 0.747058824 |
| TSTA3 | 0.060795163 | 0.634628191 |
| HSPA4L | -0.022005309 | 0.851239364 |
| DYNC1I2 | 0.190430917 | 0.247058824 |
| KIF2C | -0.564766137 | 0.043248243 |
| PSMD14 | 0.120393677 | 0.327118017 |
| PTMS | 0.489344301 | 0.010099889 |
| RAB7A | 0.095880364 | 0.28845727 |
| FTO | -0.25572679 | 0.077247503 |
| ADSL | -0.135874989 | 0.193155753 |
| HEL-S-5;ACY1;ABHD14A-ACY1 | 0.40589909 | 0.016278209 |
| HEL-S-130P;CTSD | 0.307076447 | 0.052386238 |
| UBE3A | -0.145747235 | 0.27754347 |
| UROD | -0.094543275 | 0.518128006 |
| DPYSL2 | -0.204684 | 0.087384388 |
| VDAC1 | 0.207363202 | 0.070514243 |
| GALNT2 | -0.046436246 | 0.741768405 |
| MLLT4 | 0.073137506 | 0.611949686 |
| NFKB2 | 0.266664061 | 0.035516093 |
| TLN1 | 0.085437445 | 0.328523862 |
| HBA2;HBA1 | -8.029708741 | 0.161339253 |
| ABCF1 | 0.055231503 | 0.640584536 |
| DBP2;DHX16 | -0.110633396 | 0.386459489 |
| MRPS18B | -0.058345036 | 0.830928598 |
| NELF-E;NELFE;RDBP | 0.018342888 | 0.81590825 |
| C6orf11;WDR46 | 0.232952213 | 0.589678135 |
| PRRC2A | -0.0333538 | 0.757158713 |
|  | 0.258285829 | 0.052830189 |
| ME2 | 0.050136524 | 0.668923418 |
| POLR2B | -0.251967801 | 0.035257122 |
| TCEA1 | 0.362749893 | 0.008435072 |
| BCLAF1 | -0.077783929 | 0.575804661 |
| DDX17 | 0.237280566 | 0.053681095 |
| GCSH | -0.247570606 | 0.063485017 |
| C11orf54 | 0.301981114 | 0.022197558 |
| SMN2;SMN1 | -0.538571035 | 0.005327414 |
| SLC29A1 | -0.381167197 | 0.053533111 |
| HCFC1 | -0.143376503 | 0.258157603 |
| RPS27A;HEL112 | -0.155314561 | 0.320051794 |
| PHGDH;HEL-S-113;PGDH3 | 0.20689233 | 0.08409175 |
| PPT1 | -0.265131402 | 0.152682205 |
| RFT1 | -0.329906956 | 0.039992601 |
| HLA-B;HLA;B-1510;HLA-Bw62.1;HLA-Bw62.3;HLA-B*1513;HLA-B*3512;B-3501;HLA-Bw62.4;HLA-Bw62.5;HLA-B35;HLA-B15;HLA-B*1522;HLA-B*15MD;HLA-C;DKFZp686N10220;HLA-B1501V2;HLA-B*15UL;HLA-B*15IL;HLA-B*5603 | -0.1560625 | 0.246429893 |
|  | -0.137550485 | 0.252460229 |
|  | 0.036644432 | 0.687384388 |
|  | 0.568314041 | 0.37136515 |
| ROCK2 | -0.296456576 | 0.111357751 |
| GOLGA2 | 0.141111076 | 0.431520533 |
| HARS2 | -0.267217196 | 0.167850536 |
| OPA1 | -0.226309597 | 0.288605253 |
| HEL-S-64p;GSS | 0.327885923 | 0.019607843 |
| CCDC9 | -0.061343077 | 0.743951165 |
| CTCF;CTCFL | -0.070160997 | 0.862116167 |
| POLR1C | -0.091578029 | 0.375064743 |
| RDX | -0.001209643 | 0.990381058 |
| ERCC3 | -0.412875931 | 0.391490936 |
| SUCLA2;DKFZp686D0880 | -0.028172933 | 0.812097669 |
| PRPS1 | -0.097629624 | 0.347058824 |
| RPS14 | -0.150381653 | 0.272660007 |
| CSNK2A1;CSNK2A3 | -0.009381183 | 0.99145394 |
| CCNH | -0.008019761 | 0.952904181 |
| MICAL2 | -1.052031 | 0.024380318 |
| SEC23B | 0.04099333 | 0.723788383 |
| TPP1 | -0.029946389 | 0.8300037 |
| FAM208B | -0.216319257 | 0.249426563 |
| CD59 | 0.325853001 | 0.054716981 |
| ARHGAP4;C1 | 0.003338761 | 0.962745098 |
| ATOX1 | -0.281028432 | 0.991860895 |
| DBN1 | -0.032517788 | 0.783018868 |
| SEC62;TLOC1 | 0.104640442 | 0.296263411 |
| CD3EAP | -0.289288578 | 0.035738069 |
| ESF1 | -0.105520437 | 0.420532741 |
| UBE2V2;UBE2V1 | 0.221857093 | 0.030262671 |
| SMU1 | -0.270573511 | 0.03499815 |
| WDR57;SNRNP40 | -0.001229592 | 0.987125416 |
| KIAA1598 | 0.241740254 | 0.540140585 |
| RSL1D1 | 0.176232665 | 0.135442101 |
| MRPL1 | 0.138810677 | 0.661931188 |
| CROCC | -0.008901144 | 0.873584906 |
| ILVBL | 0.189252064 | 0.253533111 |
| QRICH1 | -0.536954241 | 0.124676286 |
| PRNP | 0.042395689 | 0.658490566 |
| RBM34 | 0.100320263 | 0.598261191 |
| RPS6 | -0.055491612 | 0.726193119 |
| RPL5 | -0.187168435 | 0.121568627 |
| ATP1B1 | -0.007498314 | 0.972992971 |
| DDX1 | 0.07843365 | 0.368109508 |
| TES | 0.657103134 | 0.004661487 |
| CAPZA2 | 0.081716749 | 0.634813171 |
| ARF5 | 1.703922409 | 0.005808361 |
| RPA3 | -0.189390238 | 0.216056234 |
| CBX3 | -0.349539014 | 0.023418424 |
| CHCHD3 | 0.266440252 | 0.058490566 |
| SSBP1 | -0.002323456 | 0.968664447 |
| AGK;FLJ10842 | 0.119183086 | 0.381982982 |
| ARPC1B | -0.031171556 | 0.750684425 |
| YKT6 | -0.030907532 | 0.794006659 |
| RAC1;hCG_20693 | 0.119115992 | 0.326896041 |
| RPS4Y1 | -0.273331732 | 0.025305216 |
| AGO1;EIF2C2;EIF2C3;AGO3;AGO2;AGO4 | 0.32829514 | 0.459415464 |
| SEPT7;Nbla02942;DKFZp686F17268 | 0.085474179 | 0.569441361 |
| MAP2K1 | 0.177121687 | 0.279652238 |
| EXOSC9 | -0.110655112 | 0.370847207 |
| CNOT1 | 0.053605204 | 0.593377728 |
| CEP170 | 0.102419853 | 0.364890862 |
| MRPS16 | -1.278304175 | 0.011764706 |
| PGP | -0.027719022 | 0.847206807 |
| NUDT5 | -0.633900052 | 0.003736589 |
| PRAMEF25;PRAMEF9;LOC101929983;PRAMEF26;PRAMEF20;PRAMEF6;PRAMEF5;PRAMEF11;PRAMEF4 | 0.489477708 | 0.144025157 |
| HN1L | -0.191965916 | 0.309433962 |
| SMCHD1 | 0.051644664 | 0.663596004 |
| MFN2 | -0.493416471 | 0.130336663 |
| TP53BP1 | 0.345073448 | 0.059193489 |
| PPP2R4 | 0.210829574 | 0.089641139 |
| RRBP1 | -0.086019529 | 0.450832408 |
| BID | -0.325167074 | 0.059378468 |
| GABPA | -0.112404196 | 0.328449871 |
| KNTC2;NDC80 | -0.474984648 | 0.021975583 |
| VKORC1L1 | -0.117924812 | 0.484017758 |
| TAOK3;TAOK1 | 0.387603242 | 0.092341842 |
| DIEXF | 0.186204349 | 0.305771365 |
| FLJ00075;DKFZp434K1323;WASH;DKFZp686C24272;WASH6P;WASH3P;WASH2P;WASH1;WASH4P | -0.109276743 | 0.57573067 |
| C18orf55;TIMM21 | -1.047342865 | 0.011283759 |
| VRK1 | -0.111058936 | 0.468479467 |
| PSMC4 | 0.048104265 | 0.667295597 |
| KDM1A | -0.134231292 | 0.366444691 |
| EGFR | -0.570223795 | 0.002626711 |
| HK2;DKFZp686M1669 | 0.11214858 | 0.297891232 |
| SAFB | -0.003005885 | 0.926341102 |
| WDR75 | -0.171918744 | 0.215168331 |
| GGH | -0.383974745 | 0.036367 |
| POR;DKFZp686G04235 | 0.193102051 | 0.133296337 |
| CSK | -0.173000853 | 0.147502775 |
| CROP;LUC7L3 | -0.137054526 | 0.389863115 |
| PPP2R1A | -0.024342713 | 0.800665927 |
| PTPN1 | -0.188369881 | 0.315501295 |
| BOP1 | 0.097280839 | 0.424343322 |
| RFC5 | -0.252448061 | 0.026711062 |
| GNPDA1 | 0.738971217 | 0.004735479 |
| MEMO1 | 0.012777852 | 0.984313725 |
| NUP35 | 0.094313273 | 0.553015168 |
| HEL-S-54e;PHB | 0.022457104 | 0.798113208 |
| HEL-S-69p;PPIA | -0.236491835 | 0.089419164 |
| MARS | 0.013451613 | 0.87118017 |
| NUSAP1 | -0.485723215 | 0.01154273 |
| RPL13 | 0.117663018 | 0.357084721 |
| FUS | 0.183716872 | 0.194783574 |
| PPAT | -0.973657895 | 0.002145764 |
| NIPSNAP1;GBAS | 0.440032053 | 0.165149834 |
| EMC2 | 0.643482589 | 0.142952275 |
| CERS2 | 0.008847797 | 0.918017018 |
| URB2 | 0.503289236 | 0.098076212 |
| PSMD5 | -0.319037298 | 0.014317425 |
| NCAPH | -0.240503715 | 0.060155383 |
| RALY | 0.339379191 | 0.028486866 |
| NDUFA9 | -0.218513785 | 0.334258232 |
| TPRKB | 0.424925342 | 0.128190899 |
| ATP5F1;hCG_39985 | 0.1353854 | 0.347317795 |
| SUCLG1 | 0.730283331 | 0.065482797 |
| UBE2I | -0.099554951 | 0.398335183 |
| RPS23 | 0.042662669 | 0.692748798 |
| PELP1 | -0.242942039 | 0.177469478 |
| SRSF6;HEL-S-91 | 0.15876129 | 0.112023677 |
| PPM1B;PPM1A | -0.376275389 | 0.177210507 |
| KIAA1609;TLDC1 | -0.156776088 | 0.2745468 |
| MRPL19 | -0.075908259 | 0.652608213 |
| YME1L1;FTSH | -0.287530295 | 0.03518313 |
| UMPS | -0.211048898 | 0.12027377 |
| MALT1 | -0.756712565 | 0.005105438 |
| SUGT1 | -0.143940298 | 0.269293378 |
| IARS2 | -0.145600748 | 0.444284129 |
| XPO5 | 0.053272019 | 0.717166112 |
| XPO7 | 0.150087085 | 0.169922309 |
| STAU1 | -0.2489976 | 0.020421754 |
| SFRS4;SRSF4 | 0.394692956 | 0.109840917 |
| C1QBP | -0.213237232 | 0.150721421 |
| NOSIP | -0.537262424 | 0.009766926 |
| HEL-S-94n;STIP1 | 0.184856584 | 0.092156863 |
| EMG1 | 0.184106843 | 0.188383278 |
| DHX38 | -0.056776753 | 0.726267111 |
| DHX8 | -0.273380693 | 0.037957825 |
| NT5C2 | 0.162382607 | 0.263300037 |
| MAP3K7IP1;TAB1 | -0.400816226 | 0.219940807 |
| TFRC | -1.370318276 | 0.000554939 |
| CTR9 | -0.21568451 | 0.064261931 |
| NDUFV2 | -0.181619916 | 0.712578616 |
| NDUFB10 | -0.034122545 | 0.733740289 |
| PRIM2 | -1.204429076 | 0.001516833 |
| DDX49 | -0.432558457 | 0.012467629 |
| RUFY1 | 0.27063603 | 0.036625971 |
| KPNA2 | -0.474865568 | 0.037328894 |
| EIF4A1 | -0.250202909 | 0.054605993 |
| 10-Sep | -0.047122537 | 0.831890492 |
| RPL14 | 0.082671303 | 0.448279689 |
| NUCB1 | 0.498089371 | 0.020384758 |
| LMAN2 | 0.253552431 | 0.034295228 |
| SF3A3 | 0.055657151 | 0.558564558 |
| RPF2 | -0.018337634 | 0.940140585 |
| PREB | -0.141048587 | 0.746947836 |
| NAA35 | -0.064156603 | 0.489345172 |
| MANF | 0.294514914 | 0.036588975 |
| PPP2R1B | -0.205839703 | 0.088346282 |
| SRPK1 | -0.438470219 | 0.098594155 |
| G6PD | 0.063232031 | 0.461302257 |
| GLOD4 | 0.125268153 | 0.211246763 |
| RTCA | 0.120456379 | 0.654568997 |
| PFAS | -0.34091234 | 0.011801702 |
| CAND1 | -0.030079529 | 0.767887532 |
| NAA25 | 0.146354021 | 0.208620052 |
| NOC3L | -0.208294765 | 0.06400296 |
| PPP4R1 | 0.101186589 | 0.456714761 |
| MAP1S | 0.540615497 | 0.095745468 |
| RNMT | 0.213450229 | 0.148131706 |
| UBTF | 0.406191315 | 0.0190899 |
| PNN | -0.078528358 | 0.52445431 |
| TYMS | -1.182328743 | 0.005623381 |
| HBS1L;DKFZp434G247 | -1.387478674 | 0.007177211 |
| NOP56 | -0.020990327 | 0.915501295 |
| DIMT1 | -0.280436003 | 0.062116167 |
| ACSL1 | -0.380339601 | 0.139770625 |
| HERC4 | -0.921551729 | 0.324602294 |
| ZC3HAV1 | -0.147804513 | 0.296115427 |
| GTF2I | -0.142139934 | 0.357824639 |
| FAF1 | 0.104031991 | 0.322604514 |
| XPOT | -0.017815493 | 0.947872734 |
| DNAJC3 | -0.165617365 | 0.524010359 |
| WDHD1 | 0.160845455 | 0.558897521 |
| UGGT1 | -0.129647748 | 0.302293748 |
| U5-116KD;EFTUD2 | -0.241903779 | 0.033962264 |
| AUP1;DKFZp686P12272 | -0.259015173 | 0.891379948 |
| C4orf27 | 0.039201178 | 0.777099519 |
| SNRPG;SNRPGP15 | -0.481074521 | 0.029929708 |
| SEC13 | -0.014260287 | 0.913281539 |
| DRG2 | -0.302481108 | 0.07118017 |
| ARHGAP18 | 0.02052961 | 0.909655938 |
| SMC3 | 0.002434664 | 0.957602664 |
| NUDCD3;KIAA1068 | -0.660272271 | 0.077691454 |
| MYO1B | -0.657378776 | 0.006659267 |
| GRB2 | -0.186271923 | 0.153755087 |
| EIF3L | 0.141902941 | 0.174583796 |
| EWSR1 | -0.027174082 | 0.783943766 |
| CPNE1 | -0.079700862 | 0.569256382 |
| ARCN1;DKFZp686M09245 | 0.21416614 | 0.05664077 |
| RPS19 | 0.029488423 | 0.76936737 |
| MCM5 | -0.021998161 | 0.818497965 |
| PSMD10 | 1.382342192 | 0.013392527 |
| NRD1 | -0.404083337 | 0.00754717 |
| DDAH1 | 0.591934917 | 0.008176101 |
| UQCC1;UQCC | 0.198474054 | 0.354125046 |
| MRPL43 | 0.095558718 | 0.485793563 |
| EXOSC1 | -0.007447746 | 0.974509804 |
| PFDN2 | -0.121297042 | 0.300591935 |
| EPS15 | 0.468202983 | 0.056048835 |
| RPS4X | -0.07967838 | 0.486755457 |
| UQCRB | 0.252954017 | 0.015686275 |
| RPL31 | 0.033461992 | 0.790307066 |
| ARPC3 | -0.140038839 | 0.178431373 |
| HIST1H4H;HIST1H4A | -0.022176317 | 0.907695154 |
| RPS28 | 0.031669852 | 0.785571587 |
| UBE2C | -0.43326726 | 0.046429893 |
| RPS15A;hCG_1994130 | 0.140653164 | 0.147280799 |
| RNF7 | -0.167525693 | 0.361894192 |
| COPS6 | 0.106460794 | 0.495227525 |
| LSM3 | 0.01827583 | 0.833111358 |
| RAB32 | -0.020462512 | 0.91172771 |
| HEL-S-26;IDH1;HEL-216 | -0.085646127 | 0.467221606 |
| DKFZp779L0468;PRKAR1A | 0.145369153 | 0.247539771 |
| CEBPZ | -0.112089417 | 0.348464669 |
| HNRNPC;hCG_1641229 | 0.121794853 | 0.208324084 |
| CSA2;IK | -0.148120778 | 0.29681835 |
| PPM1G | -0.032605982 | 0.796115427 |
| PDHX | 0.441571913 | 0.016796152 |
| SAR1A;SARA1 | -0.052490284 | 0.662930078 |
| LSS | -0.373755173 | 0.039289678 |
| SLC9A3R1 | -0.094162193 | 0.328227895 |
| REEP5 | 0.053820953 | 0.737069922 |
| PDCD4 | -0.18242119 | 0.177062523 |
| SFRS3;SRSF3 | -0.027523451 | 0.804920459 |
|  | -0.28396486 | 0.078098409 |
| SSR1 | -0.045132859 | 0.690714021 |
| CMPK;CMPK1 | 0.139912389 | 0.186866445 |
| TRMT1L | -0.392998586 | 0.068405475 |
| PRPF3 | -0.12301529 | 0.292970773 |
| ZW10 | 0.016449871 | 0.856085831 |
| RPA2 | 0.208984418 | 0.189382168 |
| TMX2 | 0.109773073 | 0.533777284 |
| ANXA7 | -0.023242741 | 0.849278579 |
| PAP1;METTL9 | -0.394857785 | 0.018867925 |
| CAPZB | -0.004787291 | 0.96463189 |
| HMOX1 | 8.317211336 | 3.70E-05 |
| PRPF4 | -0.278967383 | 0.06082131 |
| MTHFD2 | -0.116772215 | 0.548205697 |
| BCAS2 | -0.261009393 | 0.038993711 |
| SNRPA | 0.020695579 | 0.83518313 |
| PNO1 | -0.224158572 | 0.108990011 |
| DDX6 | 0.119557296 | 0.22645209 |
| MRPL38 | -0.380010695 | 0.193414724 |
| FKBP8 | 0.080891488 | 0.577395486 |
| TRIM28 | -0.179922878 | 0.099223085 |
| DKFZp686P16143;DKFZp686C21148;DKFZp686I05169;STAG2;DKFZp686P168 | -0.124454486 | 0.392896781 |
| SYNCRIP | -0.169325732 | 0.149833518 |
| MAGEE1;MAGEC2 | -0.468567349 | 0.139955605 |
| SLC25A15 | -0.127674928 | 0.442064373 |
| HEL-S-114;TXNL1 | 0.039268532 | 0.669478357 |
| HEL-S-21;GSTO1 | -0.092706694 | 0.388161302 |
| NDUFB5 | -0.379547669 | 0.198853126 |
| HEL-S-66;CAPG | 0.196628368 | 0.10736219 |
| TMEM109 | 0.057630106 | 0.666555679 |
| FKBP4 | 0.009146941 | 0.914391417 |
| NCLN | -0.109087451 | 0.327339993 |
| YES1;FYN;SRC | 0.36045272 | 0.195227525 |
| PPP1R12A | 0.213620163 | 0.111616722 |
| PREP | -0.100092525 | 0.565667777 |
| KIF11 | -0.060880478 | 0.756011839 |
| QTRT1 | -0.198238866 | 0.340399556 |
| SNX9 | 0.707118646 | 0.00299667 |
| UTP18 | -0.298494452 | 0.031853496 |
| ACAA2 | 0.154071888 | 0.281428043 |
| PIGS;DKFZp686K20216 | -0.107600734 | 0.409803922 |
| NT5E | -0.123717636 | 0.178320385 |
| SYAP1 | -0.065527588 | 0.534443211 |
| TYMP;hCG_1988078 | 0.31527185 | 0.035035146 |
| UBE1C;UBA3 | 0.027013935 | 0.792859785 |
| KPNB1 | -0.204157161 | 0.060673326 |
| PRIM1 | -0.116210404 | 0.780207177 |
| IRF2BP1 | 0.05293312 | 0.72663707 |
| WDR11;DKFZp434L1715 | -0.046517418 | 0.793081761 |
| LARS | 0.100555949 | 0.294376619 |
| TP53RK | 0.424320827 | 0.256899741 |
| ERO1LB | -1.358239329 | 0.013873474 |
| NSUN5 | -0.236105469 | 0.10299667 |
| PSMD7 | 0.03331668 | 0.793044765 |
| CMBL | -0.897261937 | 0.111394747 |
| PSMG1 | -0.614739024 | 0.031927488 |
| USP14 | 0.232053149 | 0.040436552 |
| PRMT5 | 0.179842989 | 0.088383278 |
| PES1 | -0.130203479 | 0.152756197 |
| FAR1 | 0.515658282 | 0.02345542 |
| CIAO1 | -0.045193767 | 0.708842027 |
| NUBP2;NUBP1 | -0.648496569 | 0.006918239 |
| GRWD1;DKFZp564C172 | -0.241161219 | 0.060710322 |
| TRA2B;DKFZp686F18120 | 0.087594452 | 0.520976693 |
| SNX8 | 0.192096119 | 0.203847577 |
| CALM3;CALM2;CALM1 | -0.680012959 | 0.011838698 |
| RPN2 | 0.066668717 | 0.521531632 |
| RQCD1 | -0.062660033 | 0.580984092 |
| ZFR | 0.03929992 | 0.703477617 |
| EIF4G1;EIF4G1 variant protein | -0.043842415 | 0.654014058 |
| CNN2 | -0.300599533 | 0.00935997 |
| HEATR1 | -0.018952668 | 0.833888272 |
| SPTBN1 | -0.064622697 | 0.470292268 |
| DDX27 | 0.179319854 | 0.119237884 |
| PMPCB;DKFZp586I1223 | 0.220954559 | 0.060895302 |
| BAG2 | 0.148609204 | 0.267776545 |
| SEC23IP | -0.100207605 | 0.568590455 |
| NIP7 | -0.226844294 | 0.296707362 |
| CNOT7 | -0.131572271 | 0.522197558 |
| MTX2 | -0.260576418 | 0.299556049 |
| DNAJA3 | -0.35455007 | 0.021124676 |
| SEC24D | 0.089436602 | 0.50418054 |
| TECR | 0.059261516 | 0.657898631 |
| XRN2 | -0.265659559 | 0.019385868 |
| CRTAP | -0.002292133 | 0.995338513 |
| TMEM263 | -0.004252291 | 0.957417684 |
| SNX2 | -0.05913604 | 0.576100629 |
| RRP12 | 0.152055879 | 0.164668886 |
| NCAPD2 | -0.3684791 | 0.007029227 |
| TBRG4 | -1.022147559 | 0.023899371 |
| SLC25A12 | -0.196140568 | 0.164927858 |
| PGM3 | 0.018546698 | 0.872290048 |
| HEXIM1 | 0.457963993 | 0.148538661 |
| RNF14 | 0.246153728 | 0.275101739 |
| NOC2L | -0.103516585 | 0.292674806 |
| SEC61A1;SEC61A2 | 0.013553799 | 0.928153903 |
| UBQLN1 | 0.072530118 | 0.546503885 |
| ZMPSTE24 | 0.39219996 | 0.014872364 |
| feat;METTL13 | -0.39922324 | 0.041842397 |
| PSMD3 | 0.059835236 | 0.550351461 |
| SURF6 | 0.178654343 | 0.580947096 |
| SRA1 | -0.099014497 | 0.507214206 |
| RAP1GDS1 | -0.032270869 | 0.793858676 |
| TOR1A | 0.06006182 | 0.6618202 |
| EFTUD1 | -0.313609537 | 0.041213467 |
| TUBA1B;TUBA4A | -0.342121172 | 0.011875694 |
| UTP6 | -0.006611286 | 0.995967444 |
| GOLPH3 | 0.084923328 | 0.45664077 |
| CALU | -0.014468994 | 0.884202738 |
| HSD17B12 | 0.005743517 | 0.96799852 |
| HEL-S-269;PDIA3 | -0.048389286 | 0.578949316 |
| PUS7 | -0.176472936 | 0.154790973 |
| DKFZp667P103;SREK1 | 0.542105506 | 0.094228635 |
| DDX41 | -0.070638441 | 0.544432112 |
| TUBGCP2 | -0.657993585 | 0.003884573 |
| PTRF | 0.020133365 | 0.86718461 |
| EIF3S3;EIF3H | 0.175362189 | 0.08608953 |
| PAXBP1 | 0.083490451 | 0.741509434 |
| EIF3F | 0.17205947 | 0.23699593 |
| BCAT2 | 0.31557408 | 0.19881613 |
| NMD3 | -0.007283332 | 0.927894932 |
| C16orf62 | 0.101398783 | 0.715464299 |
| FSCN1 | -0.282824688 | 0.013207547 |
| DNAJC11 | 0.046861323 | 0.697891232 |
| CAMSAP2 | -0.13172021 | 0.339955605 |
| CCAR2 | 0.014757706 | 0.859119497 |
| NUP43 | -0.12640793 | 0.267628561 |
| ERRFI1 | 0.326719136 | 0.177358491 |
| LONP1 | 0.109206436 | 0.304735479 |
| TRG14;NDRG1 | -0.576808195 | 0.037143914 |
| SWAP70 | 0.391479107 | 0.00954495 |
| CD97 | -0.325347112 | 0.023862375 |
| SF3B4 | 0.013270484 | 0.912023677 |
| PPP2CA | -0.084709074 | 0.47872734 |
| SLC2A1 | -0.071397835 | 0.571439142 |
| FNTA | -0.248753233 | 0.091342952 |
| SLC35B2 | 0.626372778 | 0.00318165 |
| NUPL1 | -0.125292598 | 0.344839068 |
| YIPF5 | -0.290095905 | 0.423603404 |
| CCT3 | -0.068738855 | 0.463559009 |
| SMC4 | 0.033503286 | 0.771994081 |
| MCM2 | -0.133454515 | 0.238290788 |
| CDC5L | -0.043710341 | 0.630595634 |
| TMEM214 | 0.389630298 | 0.006511284 |
| CSTF1 | -0.007736383 | 0.947502775 |
| SRP54 | 0.282484456 | 0.016426193 |
| KHDRBS1 | 0.200313059 | 0.120310766 |
| RAD23B | 0.114701258 | 0.205734369 |
| DDX50;mcdrh | -0.162457151 | 0.156788753 |
| MRPL10 | -0.196372643 | 0.330188679 |
| GORASP2 | -0.01911732 | 0.957380688 |
| EIF4B | 0.070336935 | 0.589752127 |
| HEL-S-2a;PRDX2 | -0.262647825 | 0.083536811 |
| NOL6 | -0.096687769 | 0.427302997 |
| PPA2 | -0.12814057 | 0.505327414 |
| DYNLRB1;DYNLRB2 | -0.145755184 | 0.307251202 |
| KNS2;KLC1 | 0.096207839 | 0.374583796 |
| CPSF7 | 0.207713889 | 0.085793563 |
| IWS1 | -0.862262991 | 0.132556419 |
| OGDH | -0.082448972 | 0.536625971 |
| EXOC5;DKFZp666H126 | 0.053376386 | 0.634258232 |
| SQLE;ERG1;DKFZp686B0215 | -0.234735545 | 0.051609323 |
| HEL-S-45;TGM2 | -0.387518098 | 0.028782834 |
| UBE2K;HIP2 | 0.004216969 | 0.953570107 |
| HEL-S-164nA;GANAB | 0.017656563 | 0.855345912 |
| GLMN | -0.285072925 | 0.07573067 |
| IDH3A | 0.18337663 | 0.197484277 |
| GRN | 0.209534449 | 0.216759156 |
| CTSC | -0.433493657 | 0.027302997 |
| RETSAT | -0.29157386 | 0.029744728 |
| SHC1 | -0.062674421 | 0.65009249 |
| ABCD3 | -0.22906043 | 0.155900851 |
| ATP5C1 | -0.007971158 | 0.935590085 |
| CHURC1-FNTB;FNTB | -1.435302157 | 0.007436182 |
| UBE2Z | 0.422514754 | 0.005031447 |
| CDC27 | -0.058295033 | 0.667813541 |
| ZC3H11A | 0.337151599 | 0.046281909 |
| MRPL12 | 0.040708821 | 0.699297077 |
| NQO1 | 0.873668562 | 0.001220866 |
| GDI2 | -0.04472877 | 0.636884943 |
| DDX24 | -0.110993927 | 0.549944506 |
| UBQLN2 | -0.193600607 | 0.357898631 |
| MAPRE1 | 0.291353429 | 0.077580466 |
| LIG1 | 0.417602252 | 0.293562708 |
| RPL18A | -0.184477653 | 0.133481317 |
| KIF2A | 0.31657999 | 0.03063263 |
| GLUD1;GLUD2 | 0.190703127 | 0.082389937 |
| LIMA1 | 0.282997961 | 0.044802072 |
| CD44 | -0.387269767 | 0.019792823 |
| ITCH;WWP1;WWP2 | 0.014407773 | 0.873473918 |
| GLTSCR2 | 0.211534312 | 0.12426933 |
| PFN2 | -0.024597645 | 0.791083981 |
| LEO1 | -0.102097416 | 0.89126896 |
| ABHD14B;HEL-S-299 | 0.524675895 | 0.008509064 |
| TOE1 | -0.750102798 | 0.024158343 |
| RNP24;TMED2 | -0.206508733 | 0.237735849 |
| APOA1BP | 0.133404538 | 0.287421384 |
| TBP;TBPL2 | -0.281058677 | 0.078468368 |
| GHITM | 0.380893549 | 0.024195339 |
| DIAPH3 | -0.061402544 | 0.732889382 |
| PLS3 | -0.101049983 | 0.386422494 |
| STK24;HEL-S-95 | -0.317317826 | 0.019755827 |
| NCSTN | 0.026451699 | 0.771217166 |
| RBM39;DKFZp781C0423;DKFZp686A11192;RNPC2;DKFZp781I1140;DKFZp686C17209 | -0.111478569 | 0.276211617 |
| DDX19A;DDX19B;DKFZp762C1313;DKFZp686C21137;hCG_1998531 | 0.166909438 | 0.154975953 |
|  | -0.003985846 | 0.963596004 |
| HAGH | 0.834103885 | 0.056381798 |
| SDAD1 | 0.183152919 | 0.120865705 |
| ZC3H18;NHN1 | 0.644242121 | 0.305808361 |
| ANKRD17 | 0.166955211 | 0.145394007 |
| ALDH1L2 | 0.427999017 | 0.005401406 |
| CSTF2 | -0.124222811 | 0.333222346 |
| GBE1 | -0.268265163 | 0.045800962 |
| LSM12 | 0.006683805 | 0.98808731 |
| HNRNPK | -0.068028047 | 0.618867925 |
| KRI1;FLJ12949 | 0.323382376 | 0.030595634 |
| CNDP2 | 0.064428973 | 0.539289678 |
|  | -0.13676724 | 0.165482797 |
| SNRPB;SNRPN | -0.213672466 | 0.109655938 |
| ZPR1 | 0.123131959 | 0.168886422 |
| NRBP1 | 0.262789807 | 0.169034406 |
| PRMT3 | 0.235118938 | 0.171439142 |
| VBP1 | 0.094866296 | 0.405512394 |
| GRPEL1 | -0.547645459 | 0.58708842 |
| CSRP1;DKFZp686M148 | 0.025618767 | 0.829226785 |
| ASNS | 0.644656893 | 0.00517943 |
| FOXK1;KIAA0415 | 0.098944588 | 0.46281909 |
| SMARCA5 | -0.06581166 | 0.495893452 |
| EIF4G2;AAG1 | 0.132982387 | 0.122049575 |
| NDC1 | -0.47061141 | 0.020643729 |
| SUPT5H | 0.273441399 | 0.044025157 |
| HEL107;TKT | -0.085136971 | 0.392563818 |
| NLE1 | 0.030274606 | 0.813429523 |
| POLDIP3;DKFZp434G0310 | -0.32760236 | 0.063706992 |
| ERGIC1 | -0.324955511 | 0.124861265 |
| SMPD4 | -0.037243398 | 0.795708472 |
| B2M | -0.45124811 | 0.029004809 |
| FUBP1 | -0.120831615 | 0.309507954 |
| STK26;MST4 | 0.348345007 | 0.077173511 |
| APBB1IP | 0.338546536 | 0.054162042 |
| TBK1 | -0.407616161 | 0.059896411 |
| SEC24A | 0.005490689 | 0.998742138 |
| NUP133 | -0.325013416 | 0.028449871 |
| ARFIP1 | -0.231171127 | 0.949019608 |
| SLC25A24 | 0.054892073 | 0.657602664 |
| LAMP2 | 0.497073074 | 0.027672956 |
| TAF15 | 0.195075963 | 0.313947466 |
| IKBKAP;IKBKAP variant protein;DKFZp781H1425 | -0.131541243 | 0.216796152 |
| STAT3 | -0.153315098 | 0.2254532 |
| EIF2S2 | 0.0057176 | 0.986533481 |
| U2AF1;U2AF1L4;DKFZp313J1712 | 0.163195079 | 0.122900481 |
| U2AF2 | -0.18304572 | 0.259526452 |
| HDAC1 | -0.160796322 | 0.200369959 |
| YTHDF2 | 0.025350405 | 0.796485387 |
| RUVBL1 | -0.032089886 | 0.689752127 |
| SSB | -0.055727158 | 0.566925638 |
| DUSP3 | 0.278119882 | 0.177580466 |
| EML4 | -0.613826138 | 0.074028857 |
| RAN | -0.25180158 | 0.08072512 |
| AIP | -0.208208602 | 0.063411025 |
| CWC22 | -0.027773743 | 0.750203478 |
| POLR1A | -0.236625492 | 0.406585276 |
| GPHN | 0.305461007 | 0.039622642 |
| POLR2C | -0.230748705 | 0.056603774 |
| MYO1C | 0.545016544 | 0.003662597 |
| PCYOX1 | 0.52551724 | 0.249130596 |
| NIT1 | 0.654203463 | 0.006326304 |
|  | 0.259706202 | 0.029115797 |
| HEL-S-124m;HSPA9 | 0.183076095 | 0.06681465 |
| TMCO1 | 0.224648767 | 0.137994821 |
| ZC3H15 | -0.138668837 | 0.455197928 |
| TPM1 | 0.093500481 | 0.468886422 |
| GLB1 | -0.295577509 | 0.264705882 |
| NSF | 0.002875223 | 0.995671476 |
| AP2A2 | 0.215335486 | 0.069034406 |
| F11R | 0.517135504 | 0.006992231 |
| CHMP4A | 0.458699786 | 0.019163892 |
| FHL1 | 0.511413158 | 0.007695154 |
| NDUFS2 | -0.003141058 | 0.992933777 |
| TXNDC9 | 0.558055356 | 0.123825379 |
| RANBP3 | 0.256138675 | 0.19345172 |
| KIAA1715;LNP | -0.278997891 | 0.227487976 |
| NDUFB9;DKFZp566O173 | -0.112758299 | 0.456825749 |
| NPEPPS | -0.016448613 | 0.982204957 |
| FKBP5 | 0.737712996 | 0.261894192 |
| SLC2A3;SLC2A14 | 0.971256933 | 0.001960784 |
| Nbla03646;DHCR24 | -0.199353415 | 0.226489086 |
| EIF2AK2 | -0.409358004 | 0.006252312 |
| USP48 | -1.981890322 | 0.000628931 |
| GEMIN5 | 0.144321339 | 0.348612653 |
| SP3;DKFZp686O1631 | 0.027932786 | 0.797077321 |
| LPP | 0.262557124 | 0.032667407 |
| FANCI | -1.265818868 | 0.003070662 |
| PTMA;PTMAP7 | -0.152839745 | 0.345504994 |
| IMP4 | 0.21353974 | 0.328079911 |
| USP39 | 0.088268528 | 0.504032556 |
| IMMT | 0.187521843 | 0.047539771 |
| RAB3GAP1 | -0.044702311 | 0.598890122 |
| RBM12B | 0.266955617 | 0.064668886 |
| HEL-S-61;KIF5B-RET(NM_020630)_K24;R11;KIF5B-ALK;KIF5B;KIF5B-RET(NM_020630)_K23;R12;KIF5B-RET(NM_020975)_K23;R12;KIF5B-RET(NM_020630)_K22;R12;KIF5B-RET(NM_020975)_K22;R12;KIF5B-ALK_K17;A20;KIF5B-RET(NM_020630)_K16;R12;KIF5B-RET(NM_020975)_K16;R12;KIF5B-RET(NM_020630)_K15;R12;KIF5B-RET(NM_020975)_K15;R12 | -0.082399769 | 0.466888642 |
| PPP4R2 | -0.177484269 | 0.290122087 |
| CUTA | 0.246635296 | 0.286829449 |
| PCYT1A;hCG_2002711 | 0.820134947 | 0.002478727 |
| SRI | 0.176211662 | 0.294561598 |
| PPP1R7 | -0.132307431 | 0.329300777 |
| DCUN1D1 | -0.357787833 | 0.204772475 |
| DRAP1 | 0.024461632 | 0.968553459 |
| GPS1 | -0.066675373 | 0.594228635 |
| CYCS | -0.073436322 | 0.563485017 |
| MRPL39 | -0.282597446 | 0.057972623 |
| SCRN3 | -0.177422321 | 0.359082501 |
| MRPS34 | -0.147260195 | 0.284683685 |
| POLR2H | -0.41682571 | 0.025527192 |
| FAM3C | 0.30948696 | 0.052016278 |
| RPL24;HEL-S-310 | -0.015411195 | 0.904735479 |
| CCDC58 | 0.325574355 | 0.722382538 |
| PPIH | 0.020502186 | 0.83836478 |
| RPL22L1 | -0.748305723 | 0.004439512 |
| DKFZp781O2021;CTNND1 | -0.341867622 | 0.039215686 |
| YWHAZ | 0.073907644 | 0.451165372 |
| GTPBP4 | 0.018499655 | 0.888346282 |
| CDV3 | 0.209894965 | 0.123492416 |
| SPCS3 | -0.0489096 | 0.760562338 |
| SH3BGRL3;HEL-S-297 | 0.004550467 | 0.994413615 |
| RPRC1;MAP7D1 | -0.416114022 | 0.02327044 |
| MRPS15 | -0.776987352 | 0.043211247 |
| CAP1 | 0.236968858 | 0.039437662 |
| POU4F3;RBM27 | 0.263028125 | 0.181058084 |
| SPFH1;ERLIN1 | 0.120336775 | 0.167443581 |
| RNPS1 | -0.191476952 | 0.260747318 |
| AFG3L2 | 0.182923286 | 0.202441731 |
| IFI16 | -0.026951614 | 0.854975953 |
| ARHGEF2 | 0.411980087 | 0.014280429 |
| LETM1 | 0.176738688 | 0.254864965 |
| IREB2 | 4.24125836 | 0.000147984 |
| USP9X | 0.146098335 | 0.293414724 |
| CPSF1 | -0.081277411 | 0.479097299 |
| ZNF330 | 0.818905589 | 0.092082871 |
| MSMO1 | -0.643075331 | 0.052719201 |
| COX7C | 0.278746872 | 0.070551239 |
| ANAPC4 | -0.361420305 | 0.051202368 |
| EIF4E | 0.155799209 | 0.138660747 |
| HSD17B11 | 0.383403967 | 0.017536071 |
| SEPT11;SEPT6 | 0.139815829 | 0.377691454 |
| ISOC1 | -0.692352712 | 0.038845727 |
| COX7A2 | -0.044249823 | 0.732482427 |
| SETD7 | 0.247449422 | 0.644506104 |
| hCG_23833;RRM2 | -0.757618965 | 0.008842027 |
| SLC25A1 | -0.105724723 | 0.50299667 |
| HEL-S-34;PEBP1;HEL-210 | -0.178157048 | 0.093747688 |
| HBB | -11.17329205 | 0.074398816 |
| PRIC295;GCN1L1 | 0.093034469 | 0.302478727 |
| PUS1 | -0.071759098 | 0.546799852 |
| PPID | -0.077275822 | 0.542804292 |
| GFM1 | -0.222813375 | 0.119311876 |
| NDUFV1 | -0.234405784 | 0.056307806 |
| LRPPRC | -0.177195273 | 0.082204957 |
| NDUFS1 | -0.206637076 | 0.042064373 |
| TSFM | -0.164086098 | 0.24890862 |
| TFAM | 0.161956546 | 0.217203108 |
| NUDCD2 | 0.300217097 | 0.045689974 |
| ERLIN2 | 0.2640626 | 0.056418794 |
| TBCA | 0.010394712 | 0.995005549 |
| SKP1 | -0.189803448 | 0.075064743 |
| FABP5 | 0.012768004 | 0.932260451 |
| PPP1R2;PPP1R2P3 | -0.616146583 | 0.042471328 |
| UBR5 | -0.262371005 | 0.044173141 |
| PRRC2C | 0.54902724 | 0.003847577 |
| UBA5 | 0.033003652 | 0.788050314 |
| NAA50 | 0.050160634 | 0.697410285 |
| EZR;HEL-S-105;EZR-ROS1 | 0.345438512 | 0.010802812 |
| HMGB3;DKFZp779G118 | -0.037371053 | 0.7254532 |
| FAM136A | -0.28558069 | 0.045394007 |
| MRPL22 | -0.028921769 | 0.851461339 |
| U2SURP | -0.066515117 | 0.641250462 |
| TCOF1 | 0.227326299 | 0.096596374 |
| MAP4 | 0.393456198 | 0.015168331 |
| PRPF31 | -0.091728725 | 0.335812061 |
| DCTN1;DKFZp686I0746;DKFZp686E191;DKFZp686E0752 | 0.244738207 | 0.152978172 |
| PSMB7 | 0.407004692 | 0.007140215 |
| GNB2L1 | -0.199143294 | 0.138919719 |
| HADHA | 0.221177591 | 0.061376249 |
| RBBP7 | -0.062944672 | 0.646836848 |
| CCNB1;CCNB1V | -0.614380082 | 0.074213836 |
| TOP2B | 0.014766147 | 0.869959304 |
| MRPS17;hCG_1984214 | -0.682628689 | 0.156899741 |
| GSTK1;LOC51064 | -0.100762023 | 0.420754717 |
| TSN | 0.103947159 | 0.430743618 |
| FAM98A | -0.280517034 | 0.157676656 |
| EPB41L2 | -0.159714479 | 0.133962264 |
| EEF1D | -0.095824306 | 0.354790973 |
| PRMT1;HRMT1L2 | -0.272133024 | 0.040991491 |
| CLP1 | 0.07330407 | 0.551424343 |
| L27a;RPL27A | -0.09782014 | 0.321716611 |
| C11orf58;SMAP | 0.15701044 | 0.633037366 |
| CLNS1A | -1.719195041 | 0.009581946 |
| ATP5L | -0.029299581 | 0.824343322 |
| MAPK3;DKFZp686O0215 | 0.205234216 | 0.080429153 |
| FAU | -0.095804583 | 0.58808731 |
| WDR74 | -0.43580025 | 0.045357011 |
| SEC16A | -0.499233155 | 0.104254532 |
| HLA-A | -0.673163109 | 0.012171661 |
| TRRAP | -0.328655628 | 0.182500925 |
| HMGN1;hCG_17955;hCG_1979072 | 0.227139428 | 0.10099889 |
| TRIP6i1;TRIP6;DKFZp686J22257 | 0.047284694 | 0.673843877 |
| ILF2 | 0.039412019 | 0.696892342 |
| HEL-S-36;PITPNA | -0.020585433 | 0.956677765 |
| CCDC53 | 0.272245064 | 0.255419904 |
| DAD1 | 0.320210216 | 0.059785424 |
| COPS7A | 0.000260377 | 0.992415834 |
| SLC3A2 | 0.514281378 | 0.004587495 |
| MLEC | -0.186212286 | 0.228375879 |
| HMGA2 | 0.400146932 | 0.034517203 |
| HMBS;PBGD | -0.145512005 | 0.407140215 |
| TSG101 | -0.083606752 | 0.48808731 |
| TUBA1C | -0.604295852 | 0.005216426 |
| HLA-A;HLA-DQB1;HLA-B;HLA;HLA-A*31 | -0.564189466 | 0.013355531 |
| PDE12 | 0.222117335 | 0.132334443 |
| CAD | -0.302509247 | 0.029966704 |
| YLPM1 | -0.000688966 | 0.998483167 |
| DKFZp667O202;VPS29;vps29 | 0.159876856 | 0.225305216 |
| CNPY2 | -0.256868165 | 0.259008509 |
| MYL6 | -0.097213398 | 0.370329264 |
| BRE | -0.470275412 | 0.123122457 |
| FAM162A | 0.054858846 | 0.850203478 |
| KIF1A;KIF1B;KIF1Bbeta;KIF1C | -0.115554345 | 0.473621902 |
| RTN4;NOGOC;Nbla00271 | 0.315461818 | 0.019718831 |
| IGF2BP2 | 0.226857569 | 0.095523492 |
| CLASP1 | -0.416513207 | 0.16718461 |
| ARPC4-TTLL3;ARPC4 | 0.061337187 | 0.636958935 |
| CPSF6 | 0.209579994 | 0.090603034 |
| PDS5A | -0.052366666 | 0.68109508 |
| PSME4 | 0.004527116 | 0.919015908 |
| DERA | -0.12666277 | 0.281539031 |
| DKFZp686O1117;DPP3 | 0.342653161 | 0.037698853 |
| NUP160 | -0.221706708 | 0.118349982 |
| GSDMD;GSDMDC1 | -0.25239655 | 0.05945246 |
| LIN7C | 0.129898926 | 0.31509434 |
| FCF1;DKFZp686O2396 | -0.138047841 | 0.439696633 |
| ZC3H14;FLJ11806 | -0.028093283 | 0.612097669 |
| SNW1 | 0.235667903 | 0.08290788 |
| HEL-S-298;PTGR2 | -0.051199158 | 0.674509804 |
| MAX | 1.639408499 | 0.002552719 |
| NEMF | -0.427898502 | 0.155308916 |
| USP11 | 0.307518914 | 0.030965594 |
| MRPS22 | -0.091108967 | 0.400036996 |
| CELF1 | 0.011580707 | 0.906326304 |
| HNRNPH1 | -0.244995738 | 0.14136145 |
| ERC1 | 0.143891213 | 0.341472438 |
| DNM1L | -0.084721636 | 0.424824269 |
| NELFCD;TH1L | -0.163019361 | 0.304291528 |
| ZYX | 0.111684548 | 0.367110618 |
| SLC30A7 | -0.122016591 | 0.405734369 |
| EIF2B3 | -0.16666256 | 0.096448391 |
| RPL36A;RPL36A-HNRNPH2 | 0.334803137 | 0.092896781 |
| DNPH1 | 0.107598129 | 0.523011469 |
| SSSCA1 | -0.112093011 | 0.371661117 |
| PUM1 | -0.406445011 | 0.114021458 |
| PPP2R5C | 0.304673919 | 0.232371439 |
| ANP32A | 0.226966472 | 0.051979282 |
| DUT | -0.35846714 | 0.007510174 |
| AKR7A2;HEL-S-166mP | -0.092150053 | 0.464113947 |
| COMMD4 | -0.286690318 | 0.271587125 |
| RPS15A | -0.03063108 | 0.982981872 |
| RPS17 | -0.033248038 | 0.813244543 |
| COX5A | 0.062187158 | 0.553200148 |
| MACF1 | 0.141342348 | 0.337957825 |
| PML;PML-RAR;promyelocytic leukemia protein | -0.066648371 | 0.567443581 |
| EARS2;A-735G6.3 | 0.158201282 | 0.245948946 |
| EBNA1BP2 | -0.059292168 | 0.622789493 |
| RALA | 0.01128356 | 0.949315575 |
| TXN | 0.252529435 | 0.158934517 |
|  | 0.020254516 | 0.844950055 |
| HMOX2 | 0.084610977 | 0.489123196 |
| SLC25A11 | -0.002345347 | 0.9572697 |
| EIF5A;EIF5AL1;EIF5A2 | -0.004540727 | 0.983573807 |
| KDM2A | 0.111168483 | 0.369293378 |
| MIF | -0.314894917 | 0.105401406 |
| RTF1 | -0.419105817 | 0.068220496 |
| NUP88 | 0.036718496 | 0.83100259 |
| KIAA0368;ECM29 | -0.042294545 | 0.678320385 |
| PSMD9 | -0.089611683 | 0.475989641 |
| NOMO3;NOMO2;NOMO1 | 0.004625209 | 0.962930078 |
| OXA1L | -0.000250153 | 0.936403996 |
| DNAJB12 | 0.314655988 | 0.504439512 |
| OLA1;PTD004 | -0.004900552 | 0.957121717 |
| PTRH2 | 0.305697178 | 0.048057714 |
| DKFZp564E242;OTUB1 | 0.018808688 | 0.861413245 |
| TBCD | -0.072663666 | 0.497780244 |
| ICT1 | 0.488754735 | 0.139696633 |
| NT5C;HEL74 | -0.373068499 | 0.400073992 |
| HN1 | -0.470136521 | 0.06263411 |
| RPL19 | -0.096436379 | 0.573473918 |
| ARHGDIA;HEL-S-47e | -0.197923555 | 0.233407325 |
| SRSF1 | -0.1704065 | 0.219052904 |
| CHERP | -0.260474658 | 0.065889752 |
| SNRPD1 | -0.134127829 | 0.404920459 |
| MRPS23 | -0.174198939 | 0.17854236 |
| YTHDC1 | 0.189307523 | 0.223566408 |
| DKFZp686E23276;ALDH3A2 | 0.039080108 | 0.73381428 |
| MYL12A;MYL12B | -0.114182815 | 0.336588975 |
| ZNF207;DKFZp761N202 | -0.058082877 | 0.596485387 |
| EIF3A;eIF3a | 0.134188055 | 0.157343692 |
| SPC24 | -0.836214308 | 0.001553829 |
| TBCB | 0.199683445 | 0.198557159 |
| SUI1;EIF1 | 0.043743682 | 0.701479837 |
| ATP6V0A1;DKFZp686N0561 | 0.301615628 | 0.091749908 |
| RBM42 | 0.042438138 | 0.882981872 |
| TMEM161A | 0.081822866 | 0.627044025 |
| BLMH | -0.217493805 | 0.195005549 |
| EL52;HSP90AA1 | 0.203239593 | 0.107140215 |
| COPE | -0.200498799 | 0.104476508 |
| ZC3H4 | 0.022471503 | 0.829041805 |
|  | 0.833553288 | 0.002663707 |
| AP2S1 | 0.563230362 | 0.122604514 |
|  | -0.023646806 | 0.844136145 |
| EXOSC5 | -0.277181763 | 0.054236034 |
| CLPP | 0.170313711 | 0.175915649 |
| APOBEC3C | -0.503221152 | 0.181539031 |
| CSNK2B;CSNK2B-LY6G5B-1181;CSNK2B-LY6G5B--991 | 0.402533012 | 0.010395856 |
| AGPS | -0.267214238 | 0.031890492 |
| DDX39A;DDX39 | 0.253264592 | 0.038919719 |
| ACOT7 | -0.209982155 | 0.201109878 |
| STXBP3 | 0.207603853 | 0.213614502 |
| RAB27B | -1.140128139 | 0.005290418 |
| PSMD11 | -0.065519544 | 0.509027007 |
| PGRMC1 | 0.125887456 | 0.247872734 |
| DFFA | -0.165013135 | 0.220939697 |
| CLIC1 | -0.084496403 | 0.310987791 |
| SLC33A1 | 0.150041757 | 0.212800592 |
| SAP18 | 0.206471144 | 0.0763596 |
| IGF2BP3 | 0.233097564 | 0.101035886 |
| GOLIM4 | -0.166827426 | 0.329485757 |
| KPNA4 | 0.012712605 | 0.939141694 |
| HEL-S-1a;PDXK | -0.020631188 | 0.861191269 |
| TMEM194A | 0.643701103 | 0.230965594 |
| COX7A2L | 0.348134527 | 0.282241953 |
| NDUFAB1 | 0.267787422 | 0.08109508 |
| AP3D1 | -0.046581434 | 0.831372549 |
| CHD1;CHD2 | 0.04670759 | 0.655308916 |
| STX16;STX16-NPEPL1 | 0.162395986 | 0.521161672 |
| PDCD5 | -0.365205651 | 0.009433962 |
| TCERG1 | 0.201910416 | 0.125564188 |
| SCAMP3 | 0.110636051 | 0.27473178 |
| TAX1BP3 | -0.822035481 | 0.027044025 |
| TIMM23;TIMM23B | -0.247506585 | 0.3127266 |
| HAT1 | -0.033769414 | 0.742212357 |
| UQCRQ | 0.330660651 | 0.023677395 |
| HNRNPDL | -0.476110592 | 0.005771365 |
| XPO1 | -0.225909812 | 0.070329264 |
| KIAA0391 | -0.761731621 | 0.079985202 |
| ARPC2 | 0.065102758 | 0.512467629 |
| PGRMC2 | 0.256566985 | 0.044432112 |
| PFDN6;HKE2 | 0.096670066 | 0.402737699 |
| SURF4 | 0.04152339 | 0.713281539 |
| OGT | -0.467968102 | 0.009174991 |
| EIF3D | 0.081266899 | 0.483351831 |
| IPO8 | -0.200737008 | 0.166666667 |
| STX7 | -0.167204199 | 0.429596744 |
| ARPC5 | 0.145921625 | 0.245246023 |
| DHX15 | -0.052709022 | 0.575101739 |
| TTI1;KIAA0406 | -0.625228024 | 0.036440991 |
| RRP8 | -0.410254142 | 0.060192379 |
| PAPSS1 | -0.27146485 | 0.146614872 |
| SART1 | -0.063432035 | 0.562190159 |
| EEF1E1;EEF1E1-BLOC1S5 | 0.067735393 | 0.715871254 |
| HNRNPR;HNRPR | 0.081348863 | 0.465704772 |
| TGOLN2 | 0.47812172 | 0.024602294 |
| hTIM44;TIMM44 | 0.135657161 | 0.510395856 |
| PLRG1 | 0.39016413 | 0.060081391 |
| RGS10 | -0.211500652 | 0.076877543 |
| SGTA | 0.135938723 | 0.30736219 |
| ENSA | -0.326025078 | 0.265704772 |
| NARS | 0.073961944 | 0.385460599 |
| STRN | -0.139589926 | 0.418978912 |
| RRP9 | 0.326192333 | 0.049685535 |
| SCO2 | 0.065723122 | 0.620162782 |
| AKAP8 | 0.121627827 | 0.322567518 |
| NDUFS5 | -0.042817561 | 0.946207917 |
| TIMM8A | 0.146489146 | 0.227339993 |
| SPAG9 | 0.740118914 | 0.003588605 |
| FADS1 | -0.985245133 | 0.009951905 |
| ACSL4;FACL4 | -0.281080778 | 0.030151683 |
| SNX3 | 0.174418043 | 0.130262671 |
| MGEA5 | 0.080558252 | 0.438919719 |
| CDC40 | -0.60209091 | 0.082167962 |
| PLOD3;DKFZp564O1822 | 0.101788506 | 0.503107658 |
| UGDH | 0.041782526 | 0.693044765 |
| MAGEC1 | -0.144736094 | 0.272992971 |
| DKC1 | 0.029658977 | 0.799519053 |
| EDF1 | 0.097090657 | 0.461894192 |
| PFDN1 | 0.152715035 | 0.138031817 |
| HEL-S-52;WDR1 | 0.107350338 | 0.39763226 |
| CLUH | -0.004708894 | 0.942175361 |
| DNAJC13 | 0.000837086 | 0.778246393 |
| PDCD6 | -0.088469725 | 0.596744358 |
| H2AFY | 0.132580099 | 0.375471698 |
| FLNB;DKFZp686A1668 | 0.027091873 | 0.764076952 |
| NDUFS6 | -0.010340787 | 0.90536441 |
| SEC22B | -0.158193546 | 0.284942656 |
| PRPF40A | -0.100080892 | 0.444469108 |
| VPS26A | 0.13969099 | 0.315242323 |
| PSIP1 | 0.024284522 | 0.782796892 |
| NDUFS3;DKFZp586K0821 | -0.052066817 | 0.601109878 |
| HSBP1 | -0.131305724 | 0.296337403 |
| SF3B1 | 0.038882212 | 0.65209027 |
| PRKRA | -0.501708501 | 0.130595634 |
| SNRNP200 | -0.276107335 | 0.016500185 |
| TIPRL | 0.027250679 | 0.840880503 |
| UTP20 | -0.117364908 | 0.310025897 |
| CDC123 | -0.611118027 | 0.015464299 |
| EIF3G | 0.158191547 | 0.284868664 |
| HEL-S-25;CBR3 | -0.029808549 | 0.772068073 |
| STAM2 | -0.001216179 | 0.784202738 |
| DCTN3 | 0.379386648 | 0.010321865 |
| DNAJC8 | 0.287555333 | 0.04554199 |
| SMNDC1 | -0.485186446 | 0.012874584 |
| ATP5H | 0.02049411 | 0.826859046 |
| CPD | -0.189840522 | 0.206659267 |
| HEL103;SRP72 | -0.065814167 | 0.584831669 |
| LTN1 | -0.234451669 | 0.163928968 |
| TOMM70A | 0.106725685 | 0.411986681 |
| UFL1 | 0.016655223 | 0.866407695 |
| UBXN7 | -0.474640395 | 0.069626341 |
| PRPF6 | -0.199894927 | 0.108472068 |
| GLS | 0.029442917 | 0.754162042 |
| NDUFB4 | 0.021862463 | 0.829337773 |
| NDUFB8 | 1.94753049 | 0.000702923 |
| ZRANB2 | 0.221506715 | 0.066777654 |
| KIF20A | -0.112740181 | 0.332297447 |
| KIF4A | 0.406438456 | 0.236662967 |
| KAT7 | -0.67324579 | 0.016648169 |
| VAPB | -0.007872254 | 0.952312246 |
| SNAPIN | -0.175744202 | 0.206881243 |
| IPO7 | -0.282931929 | 0.021309656 |
| AHSA1 | -0.004567433 | 0.950721421 |
| PARN | -0.068849016 | 0.613355531 |
| CDK105;NSA2 | 0.519022255 | 0.005475398 |
| H6PD | -0.333461073 | 0.076174621 |
| SNAP29 | -0.019114545 | 0.895042545 |
| AP2A1 | -0.007079175 | 0.998964114 |
| TTC4 | -0.244419227 | 0.070773215 |
| BAG3 | 0.547645247 | 0.004106548 |
| AIFM1 | 0.004096117 | 0.971698113 |
| EML2 | -0.077725375 | 0.662893082 |
| HEL20;BPNT1 | 0.309982295 | 0.019015908 |
| TXNDC12 | 0.403574713 | 0.020754717 |
| MBD3 | -0.187722318 | 0.165778764 |
| TOMM40 | -0.028017634 | 0.746984832 |
| BAF53A;ACTL6A | -0.032212754 | 0.779060303 |
| HEL-S-133P;LDHA | -0.221684019 | 0.059859415 |
| HEL-75;GSR | 0.985607305 | 0.001331853 |
| HEL-S-44;SOD1 | 0.188718508 | 0.235627081 |
| HEL-S-156an;PNP | -0.155136481 | 0.267110618 |
| HEL-S-68p;PGK1 | -0.211267663 | 0.039030707 |
| AK1 | 0.050960662 | 0.794783574 |
| NRAS;HRAS | -0.114930763 | 0.505401406 |
| IL1A | 0.020317442 | 0.839030707 |
| IL1B | -1.228751643 | 0.002885683 |
| LMNA | 0.495758701 | 0.01490936 |
| MT2A;MT1X;MT1G | 0.128934786 | 0.459193489 |
| HEL-S-87p;ALDOA | 0.179818207 | 0.076470588 |
| CSTB | 0.140603606 | 0.205327414 |
| SOD2 | -0.481052765 | 0.155863855 |
| HEL-S-162eP;GAPDH | -0.117241541 | 0.237217906 |
| HEL-S-102;HSPB1 | 0.117768346 | 0.306733259 |
| RPN1 | 0.201551015 | 0.078357381 |
| GNAI2;WUGSC:H_LUCA16.1;GNAI1 | -0.219309184 | 0.047169811 |
| ATP1A1 | 0.280007295 | 0.023159452 |
| ALDH2 | -0.398234285 | 0.02426933 |
| SERPINB2 | -0.152100118 | 0.274028857 |
| SLC25A5 | 0.041379115 | 0.683425823 |
| EIF2S1 | 0.159976388 | 0.13381428 |
| HMGN2 | -0.026920554 | 0.950499445 |
| JUN | 0.1560873 | 0.349463559 |
| ITGB1 | -0.009089949 | 0.927413984 |
| HEL-S-271;ATP5B | 0.028844396 | 0.765334813 |
| S100A6 | -0.240627049 | 0.367813541 |
| PYGL | -0.091653104 | 0.29981502 |
| DBI | -0.267700836 | 0.104587495 |
| LDHB | -0.013881938 | 0.88945616 |
| H1F0 | 0.669884775 | 0.006030337 |
| TUBB;XTP3TPATP1 | -0.461030874 | 0.005586385 |
| PFN1 | -0.170378106 | 0.108842027 |
| APRT | 0.288768629 | 0.027524972 |
| EPRS | 0.311942697 | 0.021161672 |
| UQCRH | 0.065242676 | 0.649241583 |
| TPM2 | -0.263329969 | 0.278764336 |
| FH | 0.100786102 | 0.336773955 |
| SRPR | 0.065239772 | 0.545320015 |
| CYC1 | -0.108816753 | 0.352867185 |
| SNRPB2 | 0.203308721 | 0.064964854 |
| HEL113;VIM | -0.319803305 | 0.02663707 |
| GNAI3 | -0.154038583 | 0.327229005 |
| HEL-S-7;ANXA5 | 0.139513708 | 0.244654088 |
| ENO2 | -0.056154394 | 0.671883093 |
| HEL-S-22;GSTP1 | 0.05967365 | 0.587532371 |
| LGALS1 | -0.295375365 | 0.030521643 |
| GSTM1 | -0.048031533 | 0.756122826 |
| CLTA | 0.335562885 | 0.012578616 |
| ANXA4;HEL-S-274 | 0.047867745 | 0.688013319 |
| SNRPA1 | 0.080887356 | 0.529707732 |
| H2AFV;H2AFZ | -0.089492967 | 0.690085091 |
| UQCRFS1;UQCRFS1P1 | 0.109219201 | 0.306141324 |
| CT45A2;CT45A9;CT45A8;CT45A10 | -0.251548297 | 0.030891602 |
| COX5B | 0.228590393 | 0.04591195 |
| HEL-S-89n;HSPA5 | 0.039783431 | 0.651165372 |
| HEL-S-72p;HSPA8 | -0.143957499 | 0.34554199 |
| PDHB | -0.177914139 | 0.235294118 |
| DBT | 0.025126305 | 0.731890492 |
| PYGB | 0.316508924 | 0.022863485 |
| TOP1 | 0.274465801 | 0.08827229 |
| TOP2A | -0.098072123 | 0.673880873 |
| UBL4A | 0.413897757 | 0.010136885 |
| IGF2R | -0.117262602 | 0.388013319 |
| ADH5 | -0.038145173 | 0.686977432 |
| PCNA | -0.066300783 | 0.497151313 |
| HARS;HRS | -0.01532571 | 0.89663337 |
| SLC25A6 | -0.206441185 | 0.052534221 |
| IMPDH2 | -0.192032465 | 0.108768036 |
| TPR | -0.198675933 | 0.04472808 |
| ANXA3 | -0.477397256 | 0.007584166 |
| XRCC5 | 0.107894392 | 0.23100259 |
| COX4I1 | -0.021070944 | 0.830521643 |
| EEF2 | 0.0274667 | 0.781206067 |
| FDPS | -0.474946225 | 0.007806141 |
| HEL-S-165mP;AKR1A1 | -0.177401952 | 0.246836848 |
| HEL-S-30;PKM;PKM2 | 0.235966631 | 0.037661857 |
| TRA1;HEL-S-125m;HSP90B1 | -0.043993173 | 0.690048095 |
| IDE | -0.07516822 | 0.558083611 |
| COX6B1 | -0.121372727 | 0.445467999 |
| HNRNPL | 0.055072437 | 0.676840548 |
| GSPT1 | 0.219154742 | 0.053829079 |
| NME1 | 0.211704729 | 0.235109138 |
| RPS2;rps2 | -0.010776695 | 0.944321125 |
| H2AFX | 0.169288294 | 0.239770625 |
| CREB1 | 0.182190774 | 0.171217166 |
| HIST1H1B | -0.019985127 | 0.957787643 |
| HIST1H1C | -0.093481634 | 0.618460969 |
| STMN1 | -0.153944349 | 0.201590825 |
| HMGA1 | 0.270757716 | 0.18490566 |
| JUNB | 1.305349786 | 0.026341102 |
| ITGA2 | 0.336863595 | 0.052127266 |
| JUND | -0.045690987 | 0.673103959 |
| NDUFB7 | -0.184978118 | 0.144469108 |
| CEBPB | 0.336680398 | 0.047502775 |
| CTPS1 | -0.17179413 | 0.20935997 |
| DDX5;DKFZp686J01190 | -0.178741402 | 0.070292268 |
| PFKL | -0.187401017 | 0.061265261 |
| TCP1 | -0.133739045 | 0.200924898 |
| RPL35A | -0.013359316 | 0.921235664 |
| ARF4 | -0.272237335 | 0.093007769 |
| RPL7 | 0.070393363 | 0.528190899 |
| SON | 0.056585691 | 0.654162042 |
| PGAM1;hCG_2015269 | -0.146860395 | 0.258971513 |
| ATP5J | 0.133252771 | 0.331853496 |
| SRM | -0.206777296 | 0.093784684 |
| CSNK2A2 | 0.131107503 | 0.2827229 |
| BTF3 | -0.284418507 | 0.125342212 |
| M6PR | 0.42612676 | 0.018793933 |
| LMNB1 | 0.231210513 | 0.16799852 |
| GSTM3 | 0.126179439 | 0.406215316 |
| IRP1;HEL60;ACO1 | -1.120786371 | 0.011616722 |
| SDHB | -0.371545818 | 0.168072512 |
| OSBP | 0.41494677 | 0.024676286 |
| FBL | -0.214125335 | 0.172660007 |
| GART | 0.111483146 | 0.221642619 |
| NME1-NME2;NME2;NME1 | -0.149935771 | 0.162301147 |
| HNRNPA2B1;HNRPA2B1 | 0.01259752 | 0.893673696 |
| UQCRC2 | 0.131271607 | 0.189974103 |
| ITGA6 | -0.96223737 | 0.000665927 |
| SFPQ | 0.070490019 | 0.509988901 |
| TUBG1;TUBG2 | -0.341498538 | 0.009248983 |
| HEL-S-39;PPIB | 0.154025386 | 0.110210877 |
| RPS3 | 0.002544425 | 0.957343692 |
| SP100 | -0.198425679 | 0.3 |
| AHCY | 0.101491086 | 0.342101369 |
| HEL-S-15;CFL1 | -0.130773608 | 0.185423603 |
| DTYMK | -0.093191755 | 0.326045135 |
| RRM1 | -0.753703402 | 0.002404735 |
| MYL9 | -0.169897102 | 0.262116167 |
| POLR2A | -0.166160628 | 0.211172771 |
| RPS12 | -0.104401753 | 0.429004809 |
| YY1;ZFP42;YY2 | 0.279132129 | 0.046170921 |
| DNAJB1 | 0.542439278 | 0.005734369 |
| HEL-S-123m;ATP5A1 | 0.073690928 | 0.442619312 |
| PSMA1;HEL-S-275 | 0.274957386 | 0.026970033 |
| PSMA4 | 0.152001414 | 0.170847207 |
| HEL70;MSN | -0.152260301 | 0.108953015 |
| HMGB2 | 0.209881147 | 0.285867555 |
| PTBP1 | 0.174993273 | 0.081871994 |
| TARS | 0.030275945 | 0.760266371 |
| EEF1G | 0.223487312 | 0.057528672 |
| YWHAQ | -0.01018704 | 0.945209027 |
| RPL10 | -0.136118537 | 0.229300777 |
| RPA1 | 0.016639079 | 0.85527192 |
| APEX1 | 0.086870092 | 0.503884573 |
| HEL-S-99n;CALR | -0.082704518 | 0.51572327 |
| CANX | 0.073082204 | 0.517573067 |
| PSMB6 | 0.44119024 | 0.00836108 |
| PSMB5 | 0.405492764 | 0.019644839 |
| HEL-S-106;LAP3 | -0.061833461 | 0.564705882 |
| TPP2 | 0.007733839 | 0.922789493 |
| MARCKS | -0.197666974 | 0.179652238 |
| HEL-S-107;ERP29 | 0.168161344 | 0.136773955 |
| HEL-S-128m;PRDX6 | -0.011124215 | 0.926156123 |
| HEL-S-10;BLVRB | 1.015670048 | 0.001109878 |
| HEL-S-55;PRDX5 | -0.142755911 | 0.331520533 |
| DDT;DDTL | -0.067708575 | 0.619681835 |
| PRDX3 | -0.039501738 | 0.765741768 |
| ATP5D | 0.027844568 | 0.824787273 |
| RPL12 | 0.121150109 | 0.281871994 |
| ECHS1 | -0.177623198 | 0.166296707 |
| NMT1 | 0.032583327 | 0.763411025 |
| HEL57;SERPINB1 | -0.595365386 | 0.011394747 |
| ALDH1B1 | -0.230286675 | 0.099667037 |
| DNAJA1;HDJ2 | -0.090247585 | 0.524232334 |
| UQCRC1 | 0.172480822 | 0.12526822 |
| HEL-S-70p;ATIC | -0.101704131 | 0.275027747 |
| YWHAB | 0.087664636 | 0.493155753 |
| SFN | 0.762263521 | 0.001294858 |
| HEL-S-43;S100A11 | 0.402116632 | 0.013281539 |
| GK;GK3P | 0.398853845 | 0.037291898 |
| CDA | -0.90877424 | 0.004957455 |
| DCTD | 0.318654536 | 0.084535701 |
| RPL9 | 0.016755577 | 0.892045875 |
| HSPA4;HEL-S-5a;HS24/p52 | 0.163759794 | 0.100110988 |
| CTNNA1 | 0.32584281 | 0.029226785 |
| RFC4 | -0.288739778 | 0.026489086 |
| RFC2 | -0.253793824 | 0.039807621 |
| RFC1 | -0.163641878 | 0.235886053 |
| RPL22 | 0.190506218 | 0.190677026 |
| GTF2F1 | -0.138678 | 0.5 |
| SPR | 0.325574364 | 0.047835738 |
| MYH10 | -0.011813924 | 0.893932667 |
| COPB2 | -0.176542663 | 0.070588235 |
| SOAT1 | -0.276484696 | 0.264039956 |
| DEK | 0.097993087 | 0.404698483 |
| hCG_23373;ARL2;ARL2-SNX15 | -0.717049114 | 0.006622272 |
| MAP2K2 | -0.267503154 | 0.04572697 |
| ATP6V1E1 | 0.410345337 | 0.015353311 |
| CPOX | -0.268056212 | 0.11172771 |
| RPL4 | -0.219164241 | 0.082981872 |
| PGM1 | -0.293299578 | 0.057306696 |
| POLR2I | -1.93639639 | 0.001442841 |
| SRP14 | -0.013708024 | 0.865556789 |
| HPCAL1;DKFZp781K1922;HPCA | -0.178425092 | 0.308768036 |
| TAGLN2 | -0.629123979 | 0.010913799 |
| ETFB | 0.494844942 | 0.004476508 |
| RBMX | -0.044968198 | 0.747317795 |
| ATP6V1A | 0.142103811 | 0.213577506 |
| RPL3;rpl3 | 0.046507263 | 0.690233074 |
| FEN1 | -0.061551228 | 0.560377358 |
| TXLNA | 0.255562818 | 0.072586016 |
| RPL13A;RPL13a | 0.048411289 | 0.570662227 |
| HEL-S-32;MDH1 | 0.146624384 | 0.114465409 |
| EIF2S3;EIF2S3L | 0.055664504 | 0.616241213 |
| EIF2D | 0.034751093 | 0.763078061 |
| BUD31 | 0.271577687 | 0.082019978 |
| NAA10 | 0.174742129 | 0.187347392 |
| IARS;DKFZp686L0869;DKFZp686L17145 | 0.071522857 | 0.384979652 |
| ECI1;DCI | 0.173066872 | 0.142767296 |
| TMPO | -0.219210109 | 0.239918609 |
| STAT1 | -0.17665629 | 0.073140954 |
| SKIV2L2 | -0.267508785 | 0.014761376 |
| CASP3;DKFZp686N1815 | 0.069767406 | 0.56799852 |
| RPS27;RPS27L;LOC392748 | 0.103929423 | 0.620347762 |
| NCAPD3 | -0.484267035 | 0.052571217 |
| RPL35 | -0.033620236 | 0.961894192 |
| PAFAH1B1 | 0.088298854 | 0.470773215 |
| MSH2 | -0.125558328 | 0.170292268 |
| MAGEA1 | -0.034125724 | 0.767221606 |
| MAGEA9 | 0.164163703 | 0.258897521 |
| MKI67 | -0.610932859 | 0.00336663 |
| NOP2 | 0.094005865 | 0.402441731 |
| CRKL | -0.219394348 | 0.084424713 |
| RPL21 | -0.038734356 | 0.800776915 |
| RPL28 | 0.064975249 | 0.591749908 |
| RPS10 | -0.10885193 | 0.421124676 |
| EIF1AX;EIF1AY | -0.062643435 | 0.734369219 |
| QARS | 0.129687932 | 0.261006289 |
| ATP5O | 0.019937413 | 0.862005179 |
| LIMS1 | 0.042538518 | 0.608176101 |
| ME1 | 0.194739401 | 0.224380318 |
| GCLM | 0.826058867 | 0.003921569 |
| PSMD8;HEL-S-91n | 0.209404623 | 0.050943396 |
| HEL-S-69;CCT5 | -0.071291971 | 0.529041805 |
| HEL-S-77p;CSNK1A1;CSNK1A1L | 0.077517436 | 0.58190899 |
| IDH2 | -0.234725563 | 0.049611543 |
| MARCKSL1 | 0.059588675 | 0.628893822 |
| ALDH9A1 | 0.021547764 | 0.835701073 |
| RPIA | -0.408913504 | 0.023936367 |
| NASP | 0.053082733 | 0.623344432 |
| FASN | -0.316741008 | 0.020606733 |
| TUFM | 0.19419041 | 0.060303367 |
| ALDH7A1 | -0.388189356 | 0.032593415 |
| CENPF | -0.211066307 | 0.669737329 |
| SRP9;DKFZp564M2223 | -0.109912885 | 0.350462449 |
| AARS | 0.344459367 | 0.011431743 |
| SARS | 0.358712525 | 0.024565298 |
| PSMB3 | 0.131699764 | 0.290381058 |
| ACADVL | 0.247627353 | 0.04036256 |
| RBM25 | 0.1163906 | 0.368849427 |
| EIF2B2 | -0.025128311 | 0.983314835 |
| HINT1 | 0.152696025 | 0.238327784 |
| RANBP2 | -0.007187947 | 0.947132815 |
| SEPHS1 | -0.207869718 | 0.064520903 |
| LIG3 | -0.466045538 | 0.055752867 |
| EMD | -0.031171327 | 0.82782094 |
| DNM2 | 0.208007421 | 0.08390677 |
| NUDT2 | -0.115184725 | 0.51509434 |
| KNTC1 | -0.033096673 | 0.797447281 |
| LRBA | 0.148247179 | 0.290603034 |
| CCT8 | -0.014974385 | 0.86045135 |
| CCT4 | 0.00615848 | 0.935146134 |
| FXR2 | 0.131304717 | 0.254347022 |
| RAB13;hCG_1996054;hCG_24991 | -0.377624262 | 0.011320755 |
| DAP3 | -0.092995124 | 0.433444321 |
| HEL-S-19;GALK1 | -0.02316092 | 0.855419904 |
| SSR4 | -0.239390684 | 0.041916389 |
| BCAP31 | 0.161429828 | 0.174472808 |
| RPS6KA3 | -0.11318037 | 0.329448761 |
| HDGF | -0.061305746 | 0.553533111 |
| NDUFA8 | -0.356501881 | 0.05627081 |
| HNRNPA3 | -0.259504741 | 0.040769515 |
| PGD | 0.413266848 | 0.008694044 |
| HNRNPM;ORF | -0.045386056 | 0.67118017 |
| KPNA1 | -0.240876254 | 0.046799852 |
| MSH6;GTBP | -0.140645976 | 0.161894192 |
| SMS | 0.000834747 | 0.997780244 |
| THOP1 | -0.144767109 | 0.132482427 |
| NUP98 | -0.097046438 | 0.262338143 |
| METAP1;DKFZp781C0419 | 0.113430333 | 0.391379948 |
| COPB1 | -0.08205259 | 0.422197558 |
| COPA | 0.09644852 | 0.272955975 |
| SMTN | -0.061490846 | 0.759378468 |
| PC4;SUB1 | -0.030574652 | 0.795005549 |
| RARS | -0.165614154 | 0.091490936 |
| ATP1B3 | 0.121393769 | 0.390751017 |
| ALDH18A1 | -0.155920629 | 0.200221976 |
| PSMD4 | 0.118516489 | 0.359267481 |
| CSE1L | -0.171630316 | 0.057713651 |
| HEL-S-70;VCP;DKFZp434K0126 | 0.170768049 | 0.06045135 |
| HADHB | 0.36804784 | 0.009988901 |
| NHP2L1 | 0.111515896 | 0.392674806 |
| HNRNPH2 | -0.009153086 | 0.944654088 |
| ATP5J2;ATP5J2-PTCD1 | -0.202813408 | 0.275804661 |
| RRP1 | 0.281593752 | 0.037735849 |
| ATP5I | -0.086730604 | 0.42045875 |
| EIF6 | -0.009591862 | 0.906437292 |
| CTBP2 | -0.016067065 | 0.885941546 |
| NUP107 | -0.334134039 | 0.01509434 |
| DKFZp761E1322;MTPN | 0.039154347 | 0.790714021 |
| HEL-S-49;TPI1 | 0.025127126 | 0.873510914 |
| EIF3E | 0.184416523 | 0.091379948 |
| SEC61B | 0.162537075 | 0.220162782 |
| RPS20 | 0.071336547 | 0.599926008 |
| S100A10 | -0.202650443 | 0.439992601 |
| HEL32;DSTN | -0.236272184 | 0.062301147 |
| GMFB | 0.3529074 | 0.019126896 |
| CKS1B | -0.380004111 | 0.037402886 |
| RAB10 | 0.382429354 | 0.02863485 |
| HEL-S-71;UBE2N;UBE2NL | -0.013833161 | 0.89482057 |
| ACTR2 | -0.026097336 | 0.788383278 |
| ACTR1A | 0.130415534 | 0.176988531 |
| COPS2 | -0.177831207 | 0.141694414 |
| ABCE1 | -0.214700984 | 0.082685905 |
| DKFZp547A0616;RAP2B;RAP2A | 0.117600141 | 0.348131706 |
| RPS3A | 0.038466419 | 0.719348872 |
| RPL26;hCG_26523 | 0.006529349 | 0.968738439 |
| RPL37A | -0.010555201 | 0.930780614 |
| HSPE1;EPFP1 | -0.101470192 | 0.34491306 |
| RPL37 | -1.965801535 | 0.008583056 |
| UFM1 | 0.324615221 | 0.018534961 |
| WDR5 | 0.022859917 | 0.787495376 |
| YWHAG | 0.104453709 | 0.318312986 |
| RPS7 | -0.02622316 | 0.855567888 |
| PPP1CA | -0.126433749 | 0.292082871 |
| HEL-S-80p;PPP1CB | 0.141840333 | 0.268442471 |
| PSMC1 | 0.007788563 | 0.923307436 |
| RPS8 | -0.065187159 | 0.517499075 |
| RPS16 | -0.195287159 | 0.041398446 |
| HEL2;YWHAE;YWHAE/FAM22B fusion;YWHAE/FAM22A fusion | -0.001413331 | 0.999667037 |
| RPS18 | 0.011967722 | 0.894043655 |
| RPS29 | -0.175198568 | 0.164372919 |
| RPS13 | -0.124284634 | 0.211431743 |
| RPS11 | -0.073655644 | 0.485719571 |
| SNRPF | -0.042653783 | 0.709027007 |
| LSM6 | 0.041853081 | 0.835220126 |
| SNRPD2 | -0.22031645 | 0.142804292 |
| SNRPD3 | -0.168247387 | 0.2300037 |
| ARF6 | 0.165756502 | 0.464298927 |
| RPL7A;RP-L7a | -0.041739305 | 0.708065113 |
| POLR2G | -0.100929046 | 0.449463559 |
| ETF1 | 0.131412092 | 0.158231595 |
| RPL23A | -0.029270785 | 0.848834628 |
| RAB1A | 0.198545358 | 0.124417314 |
| RPS25 | 0.015734615 | 0.861265261 |
| RBX1 | 0.268154989 | 0.1 |
| GNB2 | 0.060451305 | 0.576026637 |
| RPL39P5;RPL39 | 0.43547398 | 0.161006289 |
| RPL10A | -0.07682024 | 0.562560118 |
| RPL11 | -0.097823512 | 0.368257492 |
| RPL8 | 0.017255098 | 0.906104329 |
| FKBP1A;FKBP12-Exin;FKBP12-Exip3;FKBP12-Exip2 | -0.113025671 | 0.513281539 |
| UBA52;UBB;RPS27A;UBC;DKFZp434K0435;UbC | 0.46675966 | 0.238734739 |
| DNCL1;DYNLL1 | -0.361454415 | 0.051313356 |
| DYNLT1 | -0.121224901 | 0.508435072 |
| RPS21 | 0.116582877 | 0.444506104 |
| ACTG1 | -0.087380242 | 0.381428043 |
| SUPT4H1 | -0.302851675 | 0.554495006 |
| TMSB10 | 0.300147523 | 0.146503885 |
| YBX1 | 0.034392318 | 0.889160192 |
| HEL-S-108;TPM4 | -0.168404768 | 0.124306326 |
| EEF1A1;EEF1A1P5;EEF1A1L14 | 0.189761498 | 0.201960784 |
| TUBB2C;TUBB4B | -0.293469087 | 0.065408805 |
| HEL-S-303;PAFAH1B2 | -0.204296497 | 0.227229005 |
| HIST2H3A;HIST3H3;HIST1H3A;H3F3B;H3F3A | -0.085532781 | 0.720347762 |
| RPP30 | -0.023222538 | 0.891601924 |
| HEL-S-100n;CCT2 | -0.096573933 | 0.415612283 |
| RAE1 | -0.063838455 | 0.620791713 |
| PRKDC | -0.02920206 | 0.807769145 |
| IFI35 | -0.191975467 | 0.405105438 |
| BASP1 | -0.070956536 | 0.701035886 |
| MRPS25 | -0.045437267 | 0.897410285 |
| MRPS5 | 0.085871379 | 0.523492416 |
| MRPS21 | -0.184349387 | 0.13499815 |
| MRPS9 | -0.16377784 | 0.177617462 |
| SARNP;CIP29 | 0.141454801 | 0.259193489 |
| LACTB | -0.003434235 | 0.994043655 |
| ARHG;RHOG | 0.075141829 | 0.491490936 |
| GTF2B | -0.073592452 | 0.891934887 |
| CDK6 | 0.829069049 | 0.30081391 |
| PURA | 0.590346552 | 0.112393637 |
| FKBP3 | 0.049241236 | 0.662523122 |
| HEL-S-95n;SORD | -0.268651701 | 0.025157233 |
| HNRNPU;HNRPU | 0.027610225 | 0.78109508 |
| TIAL1 | 0.026080209 | 0.80199778 |
| SLC7A5 | 0.522666341 | 0.005956345 |
| DKFZp666G145;DR1 | -0.103559792 | 0.466629671 |
| EXOSC10 | 0.297658627 | 0.014132445 |
| OTUD4 | -0.332820382 | 0.287902331 |
| PFKP | -0.086999442 | 0.384757677 |
| PLCB3 | 0.019016282 | 0.869256382 |
| DHODH | -0.043640003 | 0.724158343 |
| AKAP12 | -0.134387582 | 0.32745098 |
| LMNB2 | 0.238787065 | 0.162930078 |
| GLO1;HEL-S-74 | -0.047073895 | 0.707695154 |
| PTPN12 | -0.654729643 | 0.004883463 |
| SFRS11;SRSF11 | 0.065860809 | 0.643914169 |
| TP53BP2 | -0.539458928 | 0.099519053 |
| SF3A2 | 0.177615924 | 0.199926008 |
| PTPN11 | 0.036063141 | 0.734702183 |
| GFPT1 | 0.1236256 | 0.280984092 |
| PRDX1 | 0.332747793 | 0.028190899 |
| BAX;Bax | -0.197457668 | 0.095560488 |
| MCL1 | -0.257397733 | 0.19600444 |
| ARHGAP1 | -0.072424443 | 0.524935257 |
| DHX9 | -0.092494313 | 0.379356271 |
| CRYZ | 0.02781231 | 0.809285979 |
| GOLGA3 | 0.026184312 | 0.858934517 |
| VAC14 | 0.45803404 | 0.136736959 |
| NSUN2 | -0.061638546 | 0.635590085 |
| RBBP4 | -0.032850875 | 0.785867555 |
| AHNAK | 0.261703597 | 0.054199038 |
| HSPA14 | 0.171404781 | 0.126563078 |
| AP1B1 | 0.027787295 | 0.74173141 |
| PMPCA | 0.247350693 | 0.042027377 |
| KIAA0196 | -0.18952577 | 0.144099149 |
| TBL3 | -0.232708685 | 0.069737329 |
| TWF1 | -0.020642413 | 0.830558639 |
| ASPH | 0.61702465 | 0.010987791 |
| STX4;STX4A | 0.292786545 | 0.022715501 |
| AIMP1 | 0.043332545 | 0.701220866 |
| PTP4A2;PTP4A1 | 0.36281704 | 0.19245283 |
| CSTF3 | -0.163945334 | 0.113022568 |
| ECH1 | 0.568957812 | 0.003292638 |
| FLII | 0.161323368 | 0.184350721 |
| COASY | -0.41792541 | 0.024861265 |
| PRKAA1 | 0.359482876 | 0.013429523 |
| EIF2B5 | -0.047504584 | 0.632186459 |
| HNRNPA0 | -0.032779179 | 0.774805771 |
| HEL-S-97n;PRDX4 | 0.078360085 | 0.567961524 |
| PAK2 | 0.046964382 | 0.603514613 |
| STK3 | -0.463436052 | 0.040214576 |
| PSMD2;DKFZp564A2282 | -0.143090698 | 0.126119127 |
| SRSF9 | -0.261780397 | 0.091194969 |
| MAD2L1 | -0.550105099 | 0.009211987 |
| G3BP;DKFZp686L1159;G3BP1 | -0.350302171 | 0.014613393 |
| NMI | 0.498308608 | 0.097151313 |
| PABPC4 | 0.064062953 | 0.532926378 |
| EIF3I | 0.037305867 | 0.755752867 |
| CTBP1;DKFZp434B0914 | -0.558446912 | 0.067480577 |
| SF3B2;DKFZp781L0540 | -0.044711915 | 0.644617092 |
| PDAP1 | -0.141964523 | 0.157380688 |
| SQSTM1;SQSTM1-ALK | 1.164964062 | 0.003218646 |
| MTX1 | -0.008211344 | 0.951017388 |
| HEL-S-77;DCTN2 | 0.248621135 | 0.05327414 |
| NAE1 | -0.155634575 | 0.167665557 |
| HSU53209;TRA2A | -0.137577591 | 0.353015168 |
| SNX1 | -0.009772812 | 0.911949686 |
| KRR1 | 0.178976071 | 0.131594525 |
| CUL4A | -0.140932669 | 0.401812801 |
| RAB31;RAB22A | -0.176641006 | 0.318608953 |
| TPBG | 0.203564967 | 0.394487606 |
| GCS1;MOGS | -0.034166449 | 0.794413615 |
| SPTAN1 | -0.042999008 | 0.670588235 |
| GNL2 | -0.030828574 | 0.836551979 |
| EXOSC2 | -0.159657477 | 0.130706622 |
| BYSL | -0.332651809 | 0.040325564 |
| IDI1 | -0.136132903 | 0.328708842 |
| CKAP5 | 0.081072546 | 0.35863855 |
| CIRBP | -0.012553794 | 0.887643359 |
| COTL1 | 0.386606048 | 0.00617832 |
| HNRNPD;HNRPD | 0.306847187 | 0.097928228 |
| UBAP2L | 0.042232042 | 0.670477248 |
| DYNC1H1 | 0.256004323 | 0.01472438 |
| EIF2B1 | 0.218512435 | 0.051757307 |
| CTTN;EMS1 | 0.518275864 | 0.005253422 |
| RCN2 | -0.460465077 | 0.0063633 |
| TRIM25 | -0.115042254 | 0.36681465 |
| FLNC | 0.15417369 | 0.167924528 |
| FAM50A;FAM50B | 0.291423389 | 0.039659637 |
| FRG1 | -0.344781371 | 0.04890862 |
| GALE | 0.069380551 | 0.525379208 |
| CAPRIN1 | 0.071835317 | 0.470033296 |
| MCM6 | -0.076793577 | 0.516500185 |
| ITPR3 | -0.372728184 | 0.012763596 |
| TRIP12 | 0.280638789 | 0.110321865 |
| SMC1A;DKFZp686L19178 | -0.104719523 | 0.297077321 |
| RRP1B | -0.087791296 | 0.871957085 |
| PDCD11 | 0.071129754 | 0.485571587 |
| BMS1 | -0.379890736 | 0.116167222 |
| DKFZp781P1796;LPIN1 | -0.503261487 | 0.133222346 |
| USP10 | -0.064586395 | 0.587680355 |
| MESDC2 | -0.149295653 | 0.437846837 |
| MVP | 0.028920755 | 0.779097299 |
| ZNF638 | -0.321168491 | 0.511690714 |
| NUMA1 variant protein;NUMA1 | 0.1373864 | 0.211653718 |
| SLMAP | -0.270203051 | 0.278949316 |
| WTAP;DKFZp686F20131 | 0.101495082 | 0.378098409 |
| PSMD6 | 0.055765138 | 0.634924158 |
| FAM175B | 0.331268471 | 0.030743618 |
| 2-Sep | -0.022338329 | 0.880836108 |
| SART3 | -0.026335082 | 0.806474288 |
| LARS2 | 0.300053308 | 0.311468738 |
| KARS | -0.040805205 | 0.673991861 |
| RRS1 | -0.038111508 | 0.799852016 |
| EIF4H;LOC392647 | 0.051609589 | 0.756307806 |
| KIF14 | -0.224123309 | 0.068331484 |
| WDR43 | 0.080622003 | 0.545689974 |
| EEA1 | 0.120572789 | 0.275471698 |
| PDIA6 | -0.077960414 | 0.425120237 |
| PMVK | -0.154225399 | 0.246096929 |
| PLEC | -0.118582045 | 0.166333703 |
| HEL-S-66p;PPA1 | -0.340392002 | 0.006215316 |
| HEL-S-90n;QPRT | -0.143761524 | 0.894080651 |
| RAB35 | 0.442444905 | 0.012319645 |
| HEL-S-84;RCN1 | -0.150418577 | 0.283166852 |
| PCBP1 | -0.246690516 | 0.039955605 |
| PCBP2;PCBP3 | -0.019885233 | 0.828486866 |
| TCEB2 | 0.069085962 | 0.532741398 |
| SF3B3 | -0.03405095 | 0.708028117 |
| KIAA0020 | -0.055716777 | 0.588383278 |
| RSU1 | 0.019284486 | 0.828301887 |
| CNN3 | 0.257107261 | 0.03854976 |
| TRIP13 | -0.052044117 | 0.626415094 |
| ELAVL1 | 0.03946273 | 0.600739919 |
| NSDHL | -0.053330307 | 0.617314095 |
| SLC1A5 | 0.131705159 | 0.310173881 |
| TOMM34 | -0.043300388 | 0.672623011 |
| TBCE | -0.221165423 | 0.142915279 |
| VAMP3;VAMP2 | 0.03320951 | 0.757935627 |
| NEDD8;NEDD8-MDP1 | -0.42772529 | 0.017795043 |
| RAB11B;RAB11A | 0.081885411 | 0.501479837 |
| ADRM1;DKFZp686G2045 | -0.016363332 | 0.878394377 |
| PKN2 | 0.338806919 | 0.253903071 |
| DDB1 | -0.187628238 | 0.124121347 |
| SRSF7 | -0.138414405 | 0.252904181 |
| UBE2S | -0.539283052 | 0.106511284 |
| UPP1 | 0.107232648 | 0.418165002 |
| HNRNPUL2;HNRNPUL2-BSCL2 | 0.105790088 | 0.404809471 |
| LUC7L | -0.038737354 | 0.822123566 |
| PPP1R10 | 0.275142604 | 0.035146134 |
| TUBB6 | -0.41151831 | 0.013577506 |
| TSR1 | -0.381312613 | 0.008065113 |
| RELA | 0.161986889 | 0.171513134 |
| LEPRE1 | 0.117766157 | 0.462005179 |
| TUBB8 | -0.253693569 | 0.142027377 |
| DTD1 | -1.226132794 | 0.075101739 |
| LARP7;HDCMA18P | 0.298256523 | 0.059045505 |
| NADK2;FLJ30596 | 2.120619528 | 0.001738809 |
| DECR2 | 0.252340152 | 0.559859415 |
| DRIP4;PDCD6IP | 0.160188337 | 0.151572327 |
| FAM98B | -0.033936132 | 0.801627821 |
| FBXO22 | -0.092670146 | 0.579985202 |
| C17orf85 | 0.306809457 | 0.23718091 |
| ANP32E | -0.079916823 | 0.484757677 |
| ANP32B | -0.030323799 | 0.804550499 |
| NUP54;DKFZp434L1613 | 0.071406059 | 0.625009249 |
| NSFL1C | 0.160962955 | 0.10873104 |
| SNX5 | -0.386902085 | 0.033740289 |
| C11orf73 | -0.485569817 | 0.109988901 |
| MRPL11 | -0.145702157 | 0.235516093 |
| TTC1 | 0.122407134 | 0.335257122 |
| SLC25A13 | -0.084529932 | 0.461561228 |
| SCFD1 | -0.012261234 | 0.940843507 |
| DDX56 | -0.249159575 | 0.045985942 |
| NDUFA12 | 0.087704632 | 0.529559748 |
| AAAS | -0.351312667 | 0.048538661 |
| CCT7 | -0.006119994 | 0.950980392 |
| RDH14 | 0.241333863 | 0.096152423 |
| MBDin;GPN1 | -0.15777165 | 0.156233814 |
| WDR12 | -0.05549215 | 0.504291528 |
| ufd1;UFD1L | 0.134002309 | 0.289493156 |
| MST065;TOMM22 | -0.008464368 | 0.988309286 |
| C14orf166 | -0.044076883 | 0.69163892 |
| CFL2 | 0.149172571 | 0.294783574 |
| SRPRB | 0.261942922 | 0.097854236 |
| OCIAD2 | -0.830724837 | 0.001849797 |
| SMARCC1 | 0.196954918 | 0.056862745 |
| HSP90AB2P | -0.270857597 | 0.063596004 |
| LGALS8 | 0.502415522 | 0.011653718 |
|  | -0.369540397 | 0.017129116 |
| CAPN2 | -0.451207748 | 0.006067333 |
| ANXA11 | -0.274483926 | 0.07791343 |
| TIA1 | 0.266876472 | 0.141435442 |
| ZNF326 | 0.090363695 | 0.441916389 |
| MTX3 | 0.379959039 | 0.414687384 |
| DKFZp686E1893;NOP9 | -0.398734489 | 0.116315205 |
| DKFZp686E1899;TMOD3 | -0.256074418 | 0.097669256 |
| DKFZp667E1714;MLKL | 0.267937279 | 0.715279319 |
| MIA3 | 0.339877343 | 0.03718091 |
| GNAS;GSA | 0.461442109 | 0.012097669 |
| MED23 | 0.220476426 | 0.269737329 |
| DKFZp564C0482;EXOSC8 | 0.185929879 | 0.319385868 |
| DKFZp762M013;CRNKL1;crn | -0.229287477 | 0.080873104 |
| GRHPR | -0.146424635 | 0.194487606 |
| DNTTIP2 | 0.042322921 | 0.751054384 |
| EXOSC6 | -0.031112929 | 0.785719571 |
| NUP188 | -0.207734502 | 0.114354421 |
| HP1BP3 | 0.452911789 | 0.012948576 |
| NOL9 | -0.17636956 | 0.995153533 |
| THEM4 | -0.158830064 | 0.543174251 |
| UBR4;ZUBR1 | 0.31686359 | 0.022160562 |
| UBAP2 | 0.420520595 | 0.018017018 |
| C1orf57;NTPCR | -0.414472768 | 0.021050684 |
| WDR3 | -0.281005117 | 0.053607103 |
| HIST2H3PS2 | 0.107475508 | 0.444617092 |
| RIF1 | -0.676081941 | 0.004291528 |
| RPRD2 | -0.509678907 | 0.007288198 |
| RNF20 | -0.04017002 | 0.675656678 |
| BROX | 0.172412086 | 0.16600074 |
| LYPLAL1 | 1.146698072 | 0.007917129 |
| RRAGA;RRAGB | 0.824659816 | 0.011579726 |
| RBM17 | -0.001756222 | 0.972031077 |
| FLNA | -0.153718597 | 0.103847577 |
| HCTP4;TPX2 | 0.125210368 | 0.404624491 |
| TXNDC5;STRF8;DKFZp666I134;hCG_1811539 | -0.087241794 | 0.382278949 |
| DKFZp762C1015;XAB2 | 0.182257074 | 0.334665187 |
| INTS3 | -0.080354705 | 0.487902331 |
| DKFZp667H197;RBM12 | 0.091544589 | 0.490381058 |
| KIAA1429 | -0.133093381 | 0.481354051 |
| ATL3 | -0.22857759 | 0.062153163 |
| SH3GL1 | 0.411269601 | 0.008916019 |
| RABGGTA | -0.649020815 | 0.15645579 |
| NAPG | -0.188345199 | 0.111468738 |
| ADFP;PLIN2 | 1.097673735 | 0.000776915 |
| CIAPIN1 | 0.224407982 | 0.164076952 |
| TSPYL;TSPYL1 | -0.530192436 | 0.058379578 |
| CLIC4 | -0.079716102 | 0.511061783 |
| RAB1B | -0.38365354 | 0.05427303 |
| LAMTOR1 | -0.448473221 | 0.02245653 |
| FARSLA;FARSA | 0.115775563 | 0.336145024 |
| TWF2 | -0.139427709 | 0.251868294 |
| SDE2 | -0.670282755 | 0.340510544 |
| STX12 | -0.040261992 | 0.718719941 |
| NCAPG | -0.133637665 | 0.415390307 |
| EXOC4 | 0.352837902 | 0.031483537 |
| ARMC6;DKFZp762A1314 | -0.0483105 | 0.773695893 |
| ZCCHC8 | -0.332744021 | 0.17036626 |
| CDC73 | -0.308663811 | 0.037476878 |
| EDC4 | 0.005162484 | 0.968257492 |
| PRPF8 | -0.204940825 | 0.055900851 |
| FAHD1 | 0.157009132 | 0.133666297 |
| AAGAB | -0.339680259 | 0.188901221 |
| BRAT1 | -0.806461978 | 0.030706622 |
| LARP1 | -0.043698809 | 0.658527562 |
| GNE | 0.285430484 | 0.016167222 |
| FIP1L1 | 0.129304565 | 0.391342952 |
| CWC27 | -0.331847032 | 0.017758047 |
| ABCB10 | -0.032015976 | 0.883314835 |
| IKBIP;IKIP | -0.123160262 | 0.376026637 |
| IKBIP | -1.816804929 | 0.008102109 |
| UBE2R2 | 0.106020604 | 0.348871624 |
| SUPT6H | 0.232967327 | 0.074509804 |
| DDX46 | -0.114061596 | 0.226933037 |
| TRMT10C | -0.278964668 | 0.04354421 |
| EIF3M | -0.090413471 | 0.422789493 |
| CYFIP1 | 0.182269254 | 0.124010359 |
| MOB1A;MOB1B;MOBKL1A;MOB4A | -0.004840046 | 0.993488716 |
| KDM3B;JMJD1B | 0.108875237 | 0.940806511 |
| POGZ | 0.005623106 | 0.944062153 |
| NUFIP2 | 0.231481062 | 0.141065483 |
| TFG;TFG/ALK fusion | 0.307968199 | 0.027598964 |
| MAVS | -0.055921094 | 0.709840917 |
| HDGFRP2 | 0.032140235 | 0.876914539 |
| AK3 | -0.127979784 | 0.584979652 |
| IRF2BP2 | -0.199911937 | 0.109507954 |
| PATL1 | 0.146189368 | 0.224528302 |
| DPP9 | -0.722993566 | 0.030558639 |
| PABPN1 | 0.006704495 | 0.96045135 |
| PBRM1;PB1 | -0.022879215 | 0.87354791 |
| C12orf10;MST024 | -0.375411003 | 0.007991121 |
| ZNF598 | -0.081962671 | 0.517129116 |
| ALYREF | -0.048174552 | 0.711283759 |
| HOOK3 | 0.232357657 | 0.303107658 |
| THOC6 | -0.169060807 | 0.194043655 |
| CARM1 | -0.065071683 | 0.674435812 |
| DDX42 | -0.094290443 | 0.550499445 |
| DNAAF5;FLJ20397 | -0.650729314 | 0.004846467 |
| CRLF3 | -0.014873598 | 0.929078801 |
| AHNAK2 | 0.067091443 | 0.620680725 |
| CCDC50 | 0.088024445 | 0.566888642 |
|  | -0.183117205 | 0.37954125 |
| CCAR1 | -0.054613533 | 0.590418054 |
| DNAJC10 | 0.097506986 | 0.457787643 |
| RHOT2 | -0.079366604 | 0.428856826 |
| MRPL41 | -0.014018262 | 0.902108768 |
| FTSJ3 | -0.085727974 | 0.40854606 |
| GSPT2 | 0.352349888 | 0.129041805 |
| EXOC8 | 0.236714125 | 0.087717351 |
| PM20D2 | 0.08441354 | 0.37654458 |
| ALDH16A1 | -0.387881664 | 0.016056234 |
| NUP93 | -0.070051814 | 0.466740659 |
| TOMM5 | 0.29618535 | 0.086459489 |
| CISD2 | -0.42054246 | 0.006585276 |
| JAGN1 | 0.317428326 | 0.041324454 |
| MRPL50 | 0.094468281 | 0.536034036 |
| EMC1 | 0.296755609 | 0.010247873 |
| PRPF38A | 0.100505101 | 0.594672586 |
| SPATA5 | 0.031818926 | 0.812652608 |
| NHLRC2 | -0.248703758 | 0.045504994 |
| COLGALT1 | 0.057240476 | 0.612356641 |
| SCCPDH | 0.34280962 | 0.026304107 |
| SERBP1;DKFZp686P17171 | 0.058162288 | 0.609433962 |
| PDPR | 0.048616869 | 0.721531632 |
| DDX20 | -0.152010698 | 0.162486127 |
| NGDN | -0.425377152 | 0.095597484 |
| NUP37 | -0.348252738 | 0.014243433 |
| ATL2 | 0.139337442 | 0.247243803 |
| PLBD2 | 0.088717195 | 0.559711432 |
| THOC2 | -0.126196301 | 0.321420644 |
| TMEM167A | 0.019935583 | 0.829004809 |
| STT3B | 0.026977939 | 0.820680725 |
| PNPT1 | 0.065826896 | 0.558157603 |
| BICD2 | 0.003225465 | 0.968886422 |
| PLEKHO2 | 0.18344838 | 0.37317795 |
| DDX54 | -0.010316443 | 0.933370329 |
| BRIX1;BXDC2 | -0.162035775 | 0.36045135 |
| UTP15 | -0.122243692 | 0.360747318 |
| GPX8 | -0.421636281 | 0.040806511 |
| NUP210 | -0.052337306 | 0.678209397 |
| IPO4 | -0.646799838 | 0.003033666 |
| FBLIM1 | 0.184513707 | 0.279615242 |
| BRK1 | -0.159197648 | 0.352830189 |
| UBLCP1 | 0.160158034 | 0.237106918 |
| PCNP | 0.320298181 | 0.056529782 |
| ATXN2L | 0.365989011 | 0.025083241 |
| HINT2 | 0.28977725 | 0.045837958 |
| AHCTF1 | -0.134927183 | 0.162893082 |
| IRGQ | 0.033800148 | 0.82563818 |
| H1FX | -0.143771301 | 0.259119497 |
| GBF1 | -0.126542425 | 0.460266371 |
| LARP4B | 0.274934984 | 0.057824639 |
| NUP205 | -0.290221283 | 0.013762486 |
| MRPS31 | -0.177140302 | 0.283129856 |
| HEL-68;ARPC1A | -0.022302142 | 0.663152053 |
| HDAC2 | -0.207561303 | 0.071143174 |
| STAM | 0.016404806 | 0.859896411 |
| RAD50 | 0.228147889 | 0.079245283 |
| GLG1 | -0.347420322 | 0.029448761 |
| USP7 | 0.054402634 | 0.66163522 |
| CUL5 | -0.029641318 | 0.924972253 |
| MYDGF | -0.142155512 | 0.31890492 |
| RPL36AL | -0.153014432 | 0.374139845 |
| ZNF622 | 0.247693508 | 0.068775435 |
| CIRH1A | 0.12669398 | 0.202552719 |
| CCDC47 | -0.110713926 | 0.270070292 |
| MRPL24 | 0.25694821 | 0.041287458 |
| LRRC59 | 0.126590209 | 0.201849797 |
| RPE;RPEL1 | -0.008565749 | 0.971883093 |
| C18orf25 | 0.427588908 | 0.014650388 |
| SH3KBP1 | 0.124234324 | 0.302922679 |
| DCPS | -0.227875752 | 0.30754717 |
| FAF2 | 0.31319205 | 0.038956715 |
| RCN3 | -0.411524605 | 0.030225675 |
| RMDN1 | -0.05752623 | 0.67573067 |
| SEH1L | -0.129498403 | 0.398520163 |
| MRPL53 | -0.222632756 | 0.326378098 |
| L3HYPDH | 0.44464485 | 0.018460969 |
| PTCD3 | -0.131453624 | 0.328412875 |
| EDC3 | -0.162749563 | 0.282611913 |
| POP1 | 0.185442431 | 0.218719941 |
| S100A16 | -0.611491706 | 0.004624491 |
| PGM2 | 0.161632978 | 0.081576027 |
| LTV1 | -0.553132345 | 0.008657048 |
| PAGE5 | -0.049732571 | 0.813355531 |
| ZC3HAV1L | 0.238435151 | 0.516241213 |
| ERO1L | 0.068987713 | 0.545024047 |
| PGAM5 | 0.225397507 | 0.058009619 |
| DDRGK1 | -0.046860654 | 0.745800962 |
| USMG5 | -0.007067477 | 0.921309656 |
| THOC3 | -0.393419837 | 0.417018128 |
| CDK5RAP3 | 0.242909285 | 0.15763966 |
| USP47 | 0.170911775 | 0.159230485 |
| HEL164;PBK | -0.460842267 | 0.016870144 |
| SNX27 | -0.024371508 | 0.871328154 |
| PRRC1 | -0.06594031 | 0.602700703 |
| DOCK7 | -0.051128645 | 0.809322974 |
| IPO9 | -0.12886831 | 0.258342582 |
| QKI | -0.001580718 | 0.990529042 |
| FMNL2;DKFZp762B245;FMNL3 | -0.231931007 | 0.081169071 |
| VPS35;DKFZp686O2462 | 0.263381241 | 0.030373659 |
| PURB | 0.027789151 | 0.814687384 |
| NUDCD1 | -0.105840895 | 0.378209397 |
| WRNIP1 | -1.145012321 | 0.034443211 |
| SIN3A | 0.099639012 | 0.334480207 |
| MMS19 | -0.154574285 | 0.221827599 |
| PSMD1 | -0.087478174 | 0.370921199 |
| PFDN5 | -0.129802088 | 0.196707362 |
| RNF2 | -0.21117707 | 0.101257862 |
| HEL-S-67p;PARK7 | 0.095688223 | 0.30954495 |
| S100A13 | -0.206839818 | 0.148168701 |
| DNAJC7 | -0.032071913 | 0.739585646 |
| C12orf57 | 0.032179966 | 0.846318905 |
| PHB2 | 0.122761753 | 0.247132815 |
| HNRNPAB | -0.048042714 | 0.68827229 |
| MIPEP | 0.167855605 | 0.313688494 |
| C19orf43 | -0.203761878 | 0.258823529 |
| DPH2 | -0.49830047 | 0.013799482 |
| MYBBP1A | -0.264352661 | 0.021790603 |
| FARSB | 0.120954316 | 0.353718091 |
| CPPED1 | 0.055726194 | 0.640399556 |
| ERP44 | 0.248790809 | 0.032075472 |
| ALG1 | 0.018535656 | 0.864853866 |
| DIDO1 | 0.1109782 | 0.510654828 |
| FAM103A1 | -0.345167182 | 0.21154273 |
| PI4K2A | -0.238681083 | 0.204920459 |
| NDUFAF3 | 0.126925177 | 0.306067333 |
| DOHH | -0.072424057 | 0.589937107 |
| TUBB6 | -0.344116335 | 0.01809101 |
| PDCD10 | 0.088940886 | 0.425194229 |
| MRI1 | -0.293835162 | 0.068960414 |
| TRAP1 | 0.128973521 | 0.188161302 |
| NOC4L | 0.105684263 | 0.867110618 |
| UTP14A | -0.021499943 | 0.879948206 |
| SPATA5L1 | 0.09442415 | 0.512171661 |
| NUP85 | -0.207599035 | 0.088790233 |
| ELOVL1 | 0.517160039 | 0.034924158 |
| TARS2 | -0.223596343 | 0.352571217 |
| RBM4;RBM4B | 0.079454087 | 0.604439512 |
| SF3B5 | -0.398222216 | 0.456566778 |
| SRRT | 0.203127384 | 0.058342582 |
| CECR5 | -0.09102603 | 0.533888272 |
| MAK16 | -0.475890496 | 0.006289308 |
| EIF2A | -0.085520114 | 0.481982982 |
| MRPL20 | -0.248864632 | 0.079578246 |
| MRPL13 | 0.008056354 | 0.994709582 |
| MRPL9 | 0.071780526 | 0.563448021 |
| NIFK | -0.07892257 | 0.659785424 |
| SRXN1 | 3.150955669 | 0.001627821 |
| MRPS26 | 0.09799889 | 0.382574917 |
| MRPL37 | -0.169861789 | 0.253422124 |
| TBL1XR1 | -0.423363368 | 0.156048835 |
| API5 | -0.024724363 | 0.800073992 |
| DPY30;LOC84661 | -0.244750255 | 0.832556419 |
| UBE2O | -0.050045919 | 0.663263041 |
| POLR1E | -0.241329501 | 0.057195708 |
| EGLN1 | 0.113775876 | 0.503144654 |
| TMEM126A | -0.417802551 | 0.021716611 |
| LSG1 | -0.037896689 | 0.65327414 |
| NAT10 | 0.258850295 | 0.073991861 |
| ILKAP;ILKAP3 | 0.04024271 | 0.842656308 |
| IRF2BPL | -1.047998723 | 0.03081761 |
| NUCKS1;NUCKS | -0.424405659 | 0.006400296 |
| SLK | 0.038468749 | 0.753644099 |
| PDCL3 | -0.16118308 | 0.190085091 |
| RAB3GAP2 | -0.030479334 | 0.799149094 |
| DHX36 | -0.41911852 | 0.007658158 |
| MRPL46 | 0.544728688 | 0.160488346 |
| C11orf68 | -0.334704033 | 0.211061783 |
| BOLA2;BOLA2B | 0.263355198 | 0.101849797 |
| TMX1;TXNDC | -0.006985662 | 0.940510544 |
| PTPN23 | 0.173596648 | 0.095153533 |
| UNC45A | 0.053842657 | 0.614798372 |
| CHMP4B | 0.016011827 | 0.87217906 |
| POFUT1 | 0.118575504 | 0.348686644 |
| RNPEP | -0.05832577 | 0.687310396 |
| SENP3;SENP3-EIF4A1 | -0.277831614 | 0.089715131 |
| SMARCAD1 | -0.709055673 | 0.196152423 |
| CCDC86 | 0.068841933 | 0.623159452 |
| RPAP3 | -0.338545599 | 0.026785054 |
| DCTPP1 | -0.311166943 | 0.087495376 |
| WDR26 | 0.2404208 | 0.071587125 |
| PTGES2 | 0.103325615 | 0.581132075 |
| PHAX | -0.203099179 | 0.196337403 |
| ACAD9 | -0.385366325 | 0.026859046 |
| NOL11 | 0.162759064 | 0.130891602 |
| SLC25A22 | 0.211415142 | 0.059970403 |
| PANK3;PANK2;PANK1 | 0.028028184 | 0.772770995 |
| CNOT10 | 0.790132692 | 0.014502405 |
| SFXN1 | 0.142589267 | 0.171809101 |
| ELP3 | -0.693799308 | 0.002034776 |
| POLR1B | -0.206074689 | 0.147428783 |
| PPCS | 0.634901171 | 0.038068812 |
| UPF2 | 0.10719795 | 0.329078801 |
| GNB4 | -0.962506826 | 0.050055494 |
| RRAGC;RRAGD | 0.357314615 | 0.021346652 |
| ALG5 | 0.343406719 | 0.128560858 |
| NCOA5 | -0.449360614 | 0.020680725 |
| MRPL47 | -0.284511549 | 0.57791343 |
| CHMP1A | 0.327866107 | 0.020495745 |
| APMAP | 0.19916462 | 0.110062893 |
| RAB18 | -0.222494333 | 0.054568997 |
| SSU72 | -0.018454534 | 0.878246393 |
| EXOSC4 | -0.047875139 | 0.712208657 |
| NOP10 | 0.134957427 | 0.203921569 |
| OSGEP | 0.025105117 | 0.866333703 |
| NXT1;NXT2 | 0.330493793 | 0.207695154 |
| RIC8A | -0.207421803 | 0.144210137 |
| LZTFL1 | 0.172801153 | 0.288383278 |
| TIGAR | 0.404478996 | 0.174879763 |
| RPRD1B | -0.169734269 | 0.165593785 |
| HEL-S-8a;NIT2 | 0.302659446 | 0.02881983 |
| EXOSC3 | -0.898668317 | 0.040140585 |
| XPNPEP1 | 0.187252692 | 0.228597854 |
| BIRC6 | -0.387484606 | 0.097299297 |
| PDLIM7 | 0.127283556 | 0.146947836 |
| DDX21 | 0.033478854 | 0.75445801 |
| HEL-S-100;NANS | 0.04394372 | 0.728079911 |
| AASDHPPT | -0.165085619 | 0.3236404 |
| MRPL17 | -0.697336423 | 0.083573807 |
| ATG3 | -0.547154493 | 0.052682205 |
| SACM1L | 0.149931838 | 0.323233444 |
| ECHDC1 | -0.320571143 | 0.01572327 |
| MDN1 | -0.330112337 | 0.181243063 |
| IMP3 | -0.175230904 | 0.243100259 |
| SETD4 | -0.118326588 | 0.26163522 |
| ATAD3A;ATAD3B | 0.377956755 | 0.007066223 |
| RBM22 | 0.067920407 | 0.609064003 |
| SLTM | 0.125684987 | 0.398631151 |
| C1orf123 | 0.15907171 | 0.394709582 |
| NHP2 | -0.175012157 | 0.161043285 |
| ADPRHL2 | -0.439475519 | 0.045320015 |
| 5-Mar | 0.063600866 | 0.608213097 |
| CDKN2AIP | 0.456897374 | 0.008324084 |
| FAM120A | -0.095647782 | 0.437550869 |
| IGF2BP1 | 0.195771653 | 0.073103959 |
| HSPBP1 | -0.364070431 | 0.024935257 |
| MYOF | 0.375451578 | 0.005882353 |
| OGFR | -0.156595992 | 0.279208287 |
| CHMP5 | -0.129964951 | 0.362893082 |
| CWC15;HSPC148 | -0.379732513 | 0.193007769 |
| MRPL15 | -0.16356626 | 0.223381428 |
| HACD3 | -0.117375503 | 0.320754717 |
| NDUFA13 | -0.17391362 | 0.256936737 |
| KCMF1 | 0.643241789 | 0.018312986 |
| RAI14 | -0.169245598 | 0.121864595 |
| VAPA | -0.047709427 | 0.743877174 |
| ABRACL | 0.164497934 | 0.339215686 |
| DIP2B | 0.005067583 | 0.900554939 |
| BCCIP | -0.32376236 | 0.039585646 |
| ATXN10 | -0.43550571 | 0.00554939 |
| NCDN | 0.21479637 | 0.106770255 |
| COMMD3;COMMD3-BMI1 | -0.883436173 | 0.016685165 |
| GNG12 | 0.145153401 | 0.232630411 |
| EIF3K | -0.07237505 | 0.555826859 |
| DNAJB11 | -0.01305051 | 0.905623381 |
| UBA2 | 0.182897672 | 0.058231595 |
| NXF1;DKFZp667O0311 | -0.036027735 | 0.684720681 |
| PEF1 | -0.556416763 | 0.031631521 |
| UQCR10 | 0.272079828 | 0.055382908 |
| CGGBP1 | -0.076907832 | 0.544654088 |
| SRP68 | 0.179518239 | 0.073769885 |
| CYB5R1 | 0.92434327 | 0.002219756 |
| PUF60 | -0.041801985 | 0.68745838 |
| BAZ1B | -0.133711778 | 0.366222716 |
| TRMT6 | -0.265491288 | 0.048871624 |
| DBNL | -0.119511911 | 0.29345172 |
| STOML2 | -0.012570949 | 0.903921569 |
| PARP4 | -0.166280254 | 0.416537181 |
| MCTS1 | 0.24208484 | 0.039067703 |
| NFU1 | -0.423542816 | 0.12863485 |
| PRPF19 | 0.109866051 | 0.364261931 |
| NENF | 0.017731223 | 0.841213467 |
| STUB1 | -0.270317321 | 0.01709212 |
| DUSP12 | 0.166993075 | 0.441842397 |
| COPS3 | -0.350284755 | 0.050684425 |
| SHOC2 | -0.122380112 | 0.805253422 |
| RUVBL2 | -0.091010938 | 0.345024047 |
| PLAA | 0.116043571 | 0.333555309 |
| NUDC;NPD011 | -0.15685506 | 0.214872364 |
| VDAC3 | -0.210951441 | 0.05863855 |
| DRG1 | -0.029283389 | 0.721864595 |
| NCKAP1 | 0.250126061 | 0.034406215 |
| EPB41L3 | -0.304698446 | 0.010284869 |
| DIS3;KIAA1008 | -0.067026108 | 0.549870514 |
| RCL1 | -0.277057066 | 0.738475768 |
| LAMTOR2 | -0.131915871 | 0.423418424 |
| POLDIP2 | -0.051557926 | 0.812837588 |
| CARHSP1 | -0.638252467 | 0.043988161 |
| THRAP3 | 0.134877732 | 0.264187939 |
| NOP58 | -0.056361574 | 0.741250462 |
| YARS2 | -0.042073671 | 0.826156123 |
| ACOT9 | -0.147754065 | 0.34354421 |
| LSM2 | -0.217737089 | 0.163263041 |
| LUC7L2 | -0.047751523 | 0.645098039 |
| TMED5 | 0.506458757 | 0.200739919 |
| SF3B6 | 0.022916825 | 0.817610063 |
| REXO2 | 0.224776491 | 0.11472438 |
| RRP15 | -0.050596751 | 0.65645579 |
| NOP16 | 0.075121364 | 0.61054384 |
| UFC1 | -0.217493669 | 0.274065853 |
| FAM96B | -0.814262708 | 0.033370329 |
| FIS1 | -0.172863292 | 0.167628561 |
| STRAP | -0.033668667 | 0.757343692 |
| RTCB | 0.146601693 | 0.118793933 |
| RABGAP1 | 0.109894201 | 0.492304846 |
| RPL36 | 0.014569319 | 0.897447281 |
| CHTOP | -0.114218983 | 0.300036996 |
| TCAF1 | -0.31905461 | 0.156751757 |
| RBM19 | -0.328965118 | 0.352127266 |
| USP15 | -0.016710692 | 0.858305586 |
| LAS1L | -0.896385018 | 0.015427303 |
| ARIH1 | 0.406259865 | 0.048945616 |
| LSM4 | -0.194536786 | 0.468146504 |
| SAMM50 | -0.18769983 | 0.290048095 |
| SUPT16H | 0.157057323 | 0.237254902 |
| TIMM8B | -0.43086923 | 0.030114687 |
| UCHL5 | -0.192973116 | 0.098335183 |
| CD2AP | -0.067135713 | 0.798446171 |
| TIMM13 | -0.257028868 | 0.063559009 |
| TRPV2 | 0.265643537 | 0.222197558 |
| COPG1;COPG | -0.036964748 | 0.701812801 |
| CFAP20 | 0.049044717 | 0.639511654 |
| SPCS1 | -2.518949736 | 0.000850906 |
| MTCH2 | 0.089733881 | 0.433555309 |
| ARFGEF2;ARFGEF1 | 0.215904477 | 0.546577876 |
| MAD1L1 | -0.602253586 | 0.116463189 |
| DYNC1LI1;DKFZp686A1525 | 0.057762283 | 0.626970033 |
| CHCHD2;CHCHD2P9 | 0.028078725 | 0.858157603 |
| SLC4A7 | 0.252979956 | 0.192526822 |
| WASF2 | 0.396098639 | 0.00854606 |
| IVNS1ABP;DKFZp686K06216 | -0.43873815 | 0.126748058 |
| PSMC3 | 0.03145555 | 0.72563818 |
| ACIN1;DKFZp667N107 | 0.108986693 | 0.327155013 |
| HSPE1-MOB4 | 0.418740218 | 0.034073252 |
| HLA-A | -0.439370927 | 0.011024787 |
| GPX1 | -0.789556608 | 0.005068442 |
