# Supplementary material for: Effects of parthenolide on amino acid metabolism and oxidative stress in lung adenocarcinoma based on quantitative proteomic analysis, targeted amino acid metabolomics, network pharmacology, and experimental validation
Source: Front Oncol. 2025 Sep 1;15:1642866. doi: 10.3389/fonc.2025.1642866 (PMC12433850; doi:10.3389/fonc.2025.1642866)
Supplement: Supplementary file 2 [file Table2.docx]

Proteins with significant differences after administration

| Gene.names | Control.1 | Control.2 | Control.3 | Treat.1 | Treat.2 | Treat.3 |
| --- | --- | --- | --- | --- | --- | --- |
| LTB4DH | 5103700 | 2097698.973 | 2230857.405 | 5776500 | 6067400 | 6038800 |
| PSAT1 | 73610000 | 80077000 | 83512000 | 124600000 | 131340000 | 137430000 |
| SERPINB8 | 13237000 | 17224000 | 15991000 | 25697000 | 23120000 | 26026000 |
| CASP8 | 76156000 | 64409000 | 64241000 | 27251000 | 41223000 | 51137000 |
| KIAA0971 | 4296000 | 2870100 | 2641700 | 2186400 | 2540000 | 573122.4563 |
| POLR3C | 2942000 | 3148500 | 3061100 | 1044583.032 | 3346500 | 764166.7788 |
| POLD1 | 11990000 | 10633000 | 11777000 | 6353000 | 6903900 | 5720900 |
| CPT1A | 9018300 | 8436900 | 9535300 | 14614000 | 15688000 | 14400000 |
| CCNK | 1968400 | 2326600 | 1711786.069 | 824290.7543 | 742263.451 | 1645400 |
| LDLR | 4471100 | 4468500 | 4673900 | 1047166.277 | 2733100 | 3626200 |
| RAB3A | 64887000 | 63055000 | 67228000 | 988827.6337 | 1104641.749 | 1027545.267 |
| POLE3 | 3804159.5 | 3751900 | 4080600 | 1350535.788 | 1099661.71 | 3227600 |
| TRIM32 | 4463600 | 3878700 | 2977900 | 789093.8995 | 1043916.906 | 974658.5283 |
| UBE2T | 11403000 | 13128000 | 14797000 | 6675200 | 6847800 | 6532000 |
| ATAD2 | 2180900 | 1884400 | 2638800 | 670858.9848 | 1545666.934 | 777458.1505 |
| ATP6V1C1 | 16419000 | 17108000 | 17984000 | 23538000 | 26634000 | 28986000 |
| RBPJ | 2876100 | 3263100 | 2950800 | 2509300 | 711991.0213 | 660765.745 |
| NDUFA4 | 7321100 | 8502100 | 10042000 | 5389200 | 4915100 | 5199300 |
| LUZP1 | 1091978.724 | 1010804.165 | 1045985.81 | 2686700 | 1795800 | 1715700 |
| GLRX | 1087055.718 | 1767400 | 2472200 | 3015800 | 2719800 | 2903200 |
| KRT18 | 4450900 | 5102400 | 4444700 | 3340100 | 1833398.978 | 1412770.036 |
| ANAPC7 | 3748700 | 3575200 | 3054500 | 2827800 | 917157.2498 | 2201900 |
| TXNRD1 | 85262000 | 89835000 | 111650000 | 250180000 | 264680000 | 285430000 |
| PIR | 2161100 | 2547500 | 2643400 | 4660500 | 6516900 | 6304000 |
| ANPEP | 3.55E+08 | 341970000 | 286080000 | 188260000 | 191270000 | 195370000 |
| ALDH1A3 | 34951000 | 37023000 | 27814000 | 22254000 | 20245000 | 22998000 |
| DAXX | 2809600 | 1659948.441 | 3142900 | 1860200 | 1290803.86 | 1499214.99 |
| SCARB2 | 4451900 | 3894000 | 4129300 | 6374100 | 6915200 | 5911700 |
| PSPH | 7908900 | 6507100 | 8827900 | 12306000 | 11858000 | 12110000 |
| TSC22D1 | 4555600 | 5637000 | 2057700 | 1997694.182 | 982276.2472 | 1971664.358 |
| LAMP1 | 41731000 | 41928000 | 35807000 | 58482000 | 59856000 | 68326000 |
| ARHGEF7 | 3928600 | 4415500 | 4315600 | 6654000 | 5880200 | 6789900 |
| GBA | 10407000 | 7916600 | 9466500 | 16946000 | 17610000 | 13846000 |
| UHRF1 | 14285000 | 14576000 | 19732000 | 9402400 | 11881000 | 10301000 |
| MAGT1 | 2945600 | 3183000 | 671317.5091 | 4967700 | 4320600 | 4429000 |
| POLA1 | 1763700 | 2317900 | 1648900 | 867203.6157 | 1418900 | 1270700 |
| COG1 | 1766400 | 1854400 | 1287300 | 1146400 | 934952.942 | 1164800 |
| TRAPPC3 | 703825.7029 | 2685200 | 1078374.516 | 5588000 | 6911700 | 4104300 |
| SBDS | 12086000 | 14181000 | 16605000 | 24964000 | 27647000 | 23532000 |
| KIAA1524 | 11614000 | 10280000 | 9873200 | 5509500 | 5875900 | 5060500 |
| FHL2 | 2411000 | 2937800 | 2948000 | 4352900 | 4691100 | 4794600 |
| RAB3B | 3220200 | 3497400 | 2473300 | 978755.0928 | 1035247.02 | 916230.0265 |
| NPC1 | 57965000 | 57375000 | 44749000 | 84611000 | 75846000 | 82683000 |
| HELLS | 10921000 | 10191000 | 12577000 | 5745500 | 5234400 | 5039900 |
| CYP51A1 | 10774000 | 10426000 | 11315000 | 4026100 | 4240100 | 3642900 |
| STXBP1 | 1812392.061 | 956959.3299 | 3410200 | 5414800 | 5160200 | 4605600 |
| HEL-S-103 | 130140000 | 120870000 | 150390000 | 316030000 | 314810000 | 314200000 |
| RBFOX2 | 2611100 | 1705400 | 2039400 | 884264.9869 | 2241806.045 | 871411.7276 |
| LGALS3BP | 4305900 | 764015.965 | 1072620.019 | 3871100 | 3695200 | 4188600 |
| PCK2 | 10524000 | 8718100 | 8080100 | 14647000 | 13736000 | 14429000 |
| GFPT2 | 36029000 | 35970000 | 33655000 | 24485000 | 24101000 | 21479000 |
| FOSL1 | 2971700 | 2150700 | 977920.4463 | 3244900 | 3334100 | 3236000 |
| ELAC2 | 6756500 | 6709700 | 8294900 | 3676000 | 5330200 | 4523800 |
| OAT | 53937000 | 55802000 | 47294000 | 30854000 | 30454000 | 32396000 |
| PSMF1 | 3962600 | 4784600 | 4037800 | 1052153.052 | 859127.2935 | 3855800 |
| GOT1 | 74862000 | 78542000 | 86422000 | 132840000 | 139090000 | 131810000 |
| HSPA13 | 3459400 | 3465900 | 3015800 | 5533500 | 4907100 | 5439200 |
| CALD1 | 6415500 | 6882600 | 6927600 | 3534100 | 3988800 | 4258400 |
| MICAL2 | 5004700 | 4744900 | 4819000 | 693596.5836 | 2158475 | 4174200 |
| TES | 3359300 | 3615500 | 3790600 | 4858300 | 6327000 | 5790800 |
| ARF5 | 1023378.761 | 4942500 | 1050946.654 | 8080900 | 7258700 | 7520200 |
| MRPS16 | 5380400 | 4224700 | 5315100 | 4311000 | 873860.6026 | 966437.0839 |
| NUDT5 | 152630000 | 156850000 | 135470000 | 91141000 | 91799000 | 103800000 |
| C18orf55 | 3536800 | 3746900 | 4130900 | 3254400 | 1231454.183 | 1037197.138 |
| GNPDA1 | 27381000 | 19211000 | 20115000 | 35584000 | 38414000 | 37335000 |
| PPAT | 9243400 | 10417000 | 11349000 | 5262100 | 4660400 | 5867900 |
| MALT1 | 4226100 | 2985100 | 3175500 | 1902800 | 2180300 | 2064200 |
| TFRC | 328710000 | 326420000 | 298630000 | 122840000 | 132190000 | 113890000 |
| PRIM2 | 6859100 | 6152600 | 5385500 | 2427700 | 3072900 | 2482700 |
| TYMS | 11039000 | 19578000 | 23099000 | 8099400 | 8341200 | 7228800 |
| HBS1L | 2890400 | 2070600 | 2956000 | 1660500 | 450842.5373 | 914790 |
| MYO1B | 17819000 | 18453000 | 14455000 | 9129400 | 12109000 | 10924000 |
| PSMD10 | 762184.8316 | 1930526.965 | 7148700 | 8752600 | 8580600 | 8322500 |
| DDAH1 | 2589000 | 2683700 | 2057189.561 | 3831900 | 3218900 | 3997300 |
| HMOX1 | 914290 | 968010 | 974180 | 289280000 | 286450000 | 335360000 |
| SNX9 | 9149700 | 7485200 | 8170000 | 13992000 | 13280000 | 13223000 |
| ERO1LB | 11062000 | 10848000 | 10940000 | 1106235.896 | 1197170.33 | 10510000 |
| PSMG1 | 4881600 | 7783900 | 4330800 | 4048000 | 3621900 | 3429500 |
| NUBP2 | 4282000 | 5434200 | 6101000 | 3508700 | 3345100 | 3236700 |
| CALM3 | 174100000 | 148160000 | 169510000 | 121150000 | 71663000 | 114130000 |
| TBRG4 | 4375500 | 3466800 | 3107900 | 662693.0016 | 3666100 | 1062897.772 |
| TUBGCP2 | 9871500 | 8136900 | 8494100 | 5142300 | 5865900 | 5788000 |
| SLC35B2 | 7632000 | 6651200 | 7625500 | 11540000 | 10846000 | 11434000 |
| CHURC1-FNTB | 5450000 | 6031400 | 4869200 | 818135.2227 | 1496525.913 | 3731290.83 |
| NQO1 | 34529000 | 36406000 | 38533000 | 66018000 | 65162000 | 69400000 |
| TOE1 | 2167300 | 1744000 | 1549700 | 614290.2323 | 1301951.517 | 1330656.913 |
| ASNS | 44223000 | 47529000 | 59556000 | 76608000 | 82134000 | 77808000 |
| NIT1 | 27252000 | 24718000 | 20745000 | 35272000 | 35095000 | 44068000 |
| SLC2A3 | 15932000 | 14384000 | 12242000 | 28596000 | 28386000 | 26455000 |
| USP48 | 4549200 | 4327000 | 3823900 | 1210503.747 | 827860.1715 | 1176767.328 |
| FANCI | 4882100 | 3678200 | 2858800 | 1353784.125 | 1785700 | 1609300 |
| PCYT1A | 11924000 | 10483000 | 12401000 | 21110000 | 18668000 | 21678000 |
| RPL22L1 | 21144000 | 18262000 | 25902000 | 13408000 | 12595000 | 12875000 |
| MRPS15 | 5500400 | 5970000 | 6127600 | 1134360.778 | 2258253.385 | 6877300 |
| IREB2 | 1183447.663 | 899809.6384 | 771329.2077 | 16693000 | 18897000 | 18397000 |
| ISOC1 | 4737500 | 4291300 | 3776800 | 1283466.559 | 2965700 | 3675500 |
| RRM2 | 13677000 | 14456000 | 22088000 | 9386300 | 10478000 | 9840000 |
| PPP1R2 | 3110800 | 2722900 | 3483300 | 1135114.994 | 2941000 | 2002400 |
| CLNS1A | 12362000 | 14129000 | 13128000 | 610697.8564 | 1040310.933 | 10382000 |
| TUBA1C | 60382000 | 70750000 | 62160000 | 46736000 | 42728000 | 37682000 |
| MAX | 645251.2419 | 947231.2897 | 1421242.186 | 3462800 | 2883800 | 3042300 |
| SPC24 | 3765300 | 4284900 | 3730300 | 2163992.32 | 2233000 | 2201400 |
| TAX1BP3 | 3151300 | 3421300 | 3483600 | 2208444.673 | 842877.6865 | 2636900 |
| TTI1 | 1540100 | 2043300 | 2240300 | 1817300 | 839911.5476 | 1118393.911 |
| SPAG9 | 21563000 | 22988000 | 28206000 | 41814000 | 40749000 | 38964000 |
| FADS1 | 2247600 | 2353400 | 3100300 | 796039.5895 | 1108377.473 | 1985816.739 |
| CDC123 | 13733000 | 16600000 | 18951000 | 9110900 | 13557000 | 9597800 |
| NDUFB8 | 818367.4021 | 948918.6563 | 1280959.099 | 4021800 | 3965900 | 3769800 |
| KAT7 | 3456500 | 3668400 | 3217800 | 2554700 | 1404547.767 | 2526600 |
| HEL-75 | 40473000 | 42866000 | 44146000 | 92658000 | 76756000 | 83025000 |
| IL1B | 90570000 | 76434000 | 53542000 | 31880000 | 29745000 | 32479000 |
| H1F0 | 34156000 | 38477000 | 47158000 | 70637000 | 62164000 | 57780000 |
| JUNB | 8443300 | 6293900 | 781710.3196 | 11341000 | 12521000 | 14492000 |
| IRP1 | 9489900 | 9107500 | 4093600 | 3991000 | 3473500 | 2969800 |
| ITGA6 | 53846000 | 53407000 | 54357000 | 28090000 | 28364000 | 26494000 |
| RRM1 | 45935000 | 49172000 | 48604000 | 27940000 | 26110000 | 31182000 |
| HEL-S-10 | 14871000 | 16186000 | 14086000 | 30229000 | 31590000 | 29453000 |
| HEL57 | 46335000 | 48063000 | 33511000 | 26358000 | 30777000 | 27525000 |
| SFN | 192910000 | 185340000 | 199370000 | 329850000 | 316330000 | 333550000 |
| CDA | 4917400 | 5236400 | 3267900 | 2456200 | 2283500 | 2409200 |
| ARL2 | 2573600 | 2305700 | 2245000 | 1637300 | 1597500 | 1099200 |
| POLR2I | 3363500 | 3360100 | 3809100 | 1269048.338 | 881407.647 | 601403.9001 |
| TAGLN2 | 223810000 | 233560000 | 154970000 | 131360000 | 132540000 | 132020000 |
| MKI67 | 12262000 | 11640000 | 13463000 | 8523600 | 7810300 | 8131700 |
| GCLM | 15245000 | 11792000 | 14871000 | 21258000 | 25333000 | 27705000 |
| RPL37 | 48881000 | 43801000 | 40173000 | 1050756.679 | 1290716.635 | 31669000 |
| PTPN12 | 25139000 | 25706000 | 20615000 | 13675000 | 15432000 | 16284000 |
| ASPH | 4036800 | 2613800 | 2900300 | 5422000 | 4671900 | 4554400 |
| SQSTM1 | 11516000 | 10439000 | 17372000 | 28818000 | 26157000 | 33207000 |
| NADK2 | 1098922.962 | 938624.8111 | 2199269.816 | 7027200 | 5953300 | 5444600 |
| OCIAD2 | 8954200 | 9052500 | 8270200 | 5179600 | 5104000 | 4490500 |
| RIF1 | 9522700 | 10395000 | 8250300 | 5410800 | 6388100 | 5830400 |
| LYPLAL1 | 830280.7227 | 671731.2073 | 1995400 | 2554100 | 2614300 | 2575100 |
| RRAGA | 1645952.944 | 3613000 | 2809400 | 4922900 | 4328800 | 5038300 |
| ADFP | 52459000 | 49102000 | 51180000 | 114530000 | 101030000 | 111320000 |
| BRAT1 | 3009100 | 3306200 | 3285400 | 2315900 | 2392100 | 781510.9184 |
| IKBIP | 13292000 | 9383000 | 7849200 | 657381.1628 | 1588579.533 | 6418300 |
| DPP9 | 5271800 | 3472700 | 4576200 | 3394200 | 3143900 | 1532100 |
| DNAAF5 | 6022300 | 5072500 | 5769100 | 3147100 | 3573400 | 4021100 |
| IPO4 | 59605000 | 62218000 | 59578000 | 37249000 | 42409000 | 36202000 |
| S100A16 | 43504000 | 35484000 | 40547000 | 27165000 | 27200000 | 23873000 |
| WRNIP1 | 6112600 | 6137800 | 2366500.514 | 4340000 | 1384305.83 | 885249.4107 |
| SRXN1 | 938681.0624 | 2204100 | 4461100 | 20299000 | 22254000 | 24988000 |
| IRF2BPL | 5848200 | 5824300 | 6445000 | 3695700 | 713522.2101 | 4353100 |
| CNOT10 | 1631120.336 | 1131068.28 | 2301500 | 2546200 | 3573500 | 2636600 |
| ELP3 | 1719600 | 1613700 | 1798500 | 1049100 | 1089700 | 1033800 |
| PPCS | 689876.3393 | 1417600 | 1726474.198 | 2134500 | 1828078.679 | 1990900 |
| EXOSC3 | 5350100 | 5954000 | 5414300 | 3963100 | 811242.3226 | 4193100 |
| KCMF1 | 2325000 | 2176200 | 2215900 | 4674700 | 2941300 | 2875000 |
| COMMD3 | 4439200 | 4737900 | 2827300 | 1404482.647 | 3087900 | 2014900 |
| CYB5R1 | 5216300 | 5967100 | 4565300 | 10538000 | 9450200 | 9900000 |
| CARHSP1 | 34532000 | 17069000 | 23060000 | 14363000 | 14363000 | 19243000 |
| FAM96B | 3404600 | 4121600 | 4521400 | 1134561.512 | 1303897.13 | 4413000 |
| LAS1L | 2959200 | 3209000 | 3529200 | 2925400 | 1087233.165 | 1197112.621 |
| SPCS1 | 4126600 | 6652300 | 4628400 | 726225.7127 | 763592.8594 | 1198291.893 |
| GPX1 | 4144200 | 4464900 | 3784900 | 2959700 | 1944300 | 2266200 |
| GCTG | 4434118 | 4054713 | 3175710 | 1864897 | 1055389 | 2076231 |
