# Supplementary material for: Effects of parthenolide on amino acid metabolism and oxidative stress in lung adenocarcinoma based on quantitative proteomic analysis, targeted amino acid metabolomics, network pharmacology, and experimental validation
Source: Front Oncol. 2025 Sep 1;15:1642866. doi: 10.3389/fonc.2025.1642866 (PMC12433850; doi:10.3389/fonc.2025.1642866)
Supplement: Supplementary file 3 [file Table3.docx]

Expression levels of amino acid metabolites after administration

| Component Name | A1 | A2 | A3 | A4 | A5 | A6 | B1 | B2 | B3 | B4 | B5 | B6 |
| --- | --- | --- | --- | --- | --- | --- | --- | --- | --- | --- | --- | --- |
| Glycine | 3.310719647 | 4.239442559 | 2.12044727 | 1.55135378 | 1.193281352 | 2.433821252 | 2.199169709 | ND | ND | 2.449515975 | 2.927292365 | 2.521642211 |
| Serine | 4.463390959 | 5.231553431 | 4.055929579 | 4.07241631 | 3.609589008 | 5.166331491 | 6.384073135 | 3.127354233 | 2.706837691 | 6.142045527 | 5.888245363 | 5.154255056 |
| Methionine | 0.946026815 | 1.41887846 | 0.896493356 | 0.870800142 | 1.274032838 | 1.120044157 | 1.682878731 | 0.92672564 | 0.473279335 | 0.928033981 | 1.015161337 | 1.043436383 |
| Proline | 16.87343652 | 18.80766893 | 14.96047353 | 13.43614643 | 13.86350288 | 13.46382052 | 6.650617641 | 4.322108469 | 4.02980008 | 6.77157372 | 6.969853693 | 6.660173471 |
| Leucine | 4.72441112 | 5.95321955 | 4.467613802 | 4.367711406 | 4.533296074 | 5.527038291 | 6.64618643 | 3.262441754 | 2.212936285 | 4.206961312 | 4.155605832 | 4.36362675 |
| Creatine | 0.617770014 | 0.862228872 | 0.565229663 | 0.495912831 | 0.755852807 | 0.472552555 | 0.245269654 | 0.202712693 | 0.128587736 | 0.207263874 | 0.224014868 | 0.235387641 |
| Glutamic | 160.3004799 | 187.9142082 | 130.8617513 | 118.2815555 | 112.9870364 | 113.7991726 | 39.67505407 | 22.42652514 | 21.93070074 | 33.61541433 | 35.45400049 | 37.51987655 |
| Phenylalanine | 2.461431175 | 2.939375325 | 2.309994734 | 2.224042589 | 2.064390359 | 3.393464056 | 3.812525491 | 1.898886498 | 1.456056948 | 2.701648605 | 2.947913445 | 2.701268417 |
| Lysine | 2.77307308 | 2.904557134 | 2.458979475 | 2.428246455 | 1.858594067 | 3.095956805 | 3.056392797 | 1.206163917 | 0.911709908 | 1.97556825 | 2.077591708 | 1.911329455 |
| Aminobutyric | 0.523956604 | 0.601695328 | 0.448714459 | 0.39839983 | 0.380869884 | 0.335616918 | 0.080717187 | ND | ND | 0.081496273 | 0.091768847 | 0.097788551 |
| Argine | 9.838120906 | 11.18808584 | 9.25978238 | 8.647487201 | 6.674037779 | 9.240291565 | 5.851545707 | 2.678791443 | 2.747849159 | 5.545487239 | 5.314781857 | 5.364203254 |
| Tryptophan | 0.598249314 | 0.60993264 | 0.495744709 | 0.457440097 | 0.496852285 | 0.584370466 | 0.441704419 | 0.180664846 | 0.150420986 | 0.269070388 | 0.282176353 | 0.290078719 |
| Tyrosine | 1.913104678 | 2.769018022 | 1.862245454 | 1.767018617 | 2.548131351 | 2.216569547 | 3.096398837 | 1.837259799 | 1.027341615 | 1.855121008 | 1.989403842 | 2.066768221 |
| Histidine | 0.796455663 | 1.160674983 | 0.722799797 | 0.710398063 | 0.975620582 | 0.819216797 | 0.883301333 | 0.539323726 | 0.322610168 | 0.631678296 | 0.664530079 | 0.693660389 |
| Valine | 1.005558528 | 1.453298524 | 0.97297726 | 0.937287737 | 1.391779024 | 1.260980406 | 2.009517477 | 1.292121514 | 0.698343076 | 1.219195622 | 1.2954229 | 1.318747765 |
| Ornithine | 1.011405085 | 1.396670694 | 0.979809597 | 0.928088293 | 1.14808533 | 0.709875011 | 0.321950343 | 0.426888382 | 0.366201951 | 0.681300809 | 0.557287358 | 0.551427711 |
| Alanine | 3.300103365 | 3.844246068 | 3.02436973 | 2.807213345 | 2.791983086 | 3.261130495 | 3.689231553 | 1.806103737 | 1.532939256 | 3.022453381 | 3.194130768 | 2.854958901 |
| Taurine | 15.38832823 | 19.35029397 | 14.08667358 | 12.02754233 | 14.01220344 | 11.54410501 | 4.923144368 | 3.319754659 | 3.128383278 | 4.741955152 | 4.94564178 | 5.382026849 |
| Isoleucine | 2.981826759 | 3.551004977 | 2.691754593 | 2.586224152 | 2.420607613 | 2.904363302 | 2.689882482 | 1.292061812 | 1.078057769 | 2.026804441 | 2.0509465 | 1.977657366 |
| Aspartic | 8.436342935 | 12.20129716 | 9.413355114 | 9.582103518 | 7.879279383 | 6.903558983 | 2.389984824 | 2.124708113 | 1.821033909 | 4.199083964 | 3.843230196 | 4.41553396 |
| Threonine | 3.017828515 | 3.593258509 | 2.722095006 | 2.622770329 | 2.311474935 | 3.460826635 | 4.063453944 | 2.069895941 | 1.751754011 | 3.431080393 | 3.448450173 | 3.220115935 |
| glutamine | 2.82717564 | 2.921036669 | 2.563668145 | 2.520418769 | 1.960709945 | 3.208106862 | 3.122315074 | 1.21908475 | 0.934064483 | 1.984538572 | 2.155268122 | 1.95292133 |
| asparagine | 3.460779808 | 3.855978205 | 3.011251155 | 2.852552967 | 2.49872172 | 3.026077202 | 2.774578521 | 1.228823119 | 1.087669111 | 2.144029896 | 2.178533629 | 2.022127676 |
