# Supplementary figures and images for: Effects of parthenolide on amino acid metabolism and oxidative stress in lung adenocarcinoma based on quantitative proteomic analysis, targeted amino acid metabolomics, network pharmacology, and experimental validation
Source: Front Oncol. 2025 Sep 1;15:1642866. doi: 10.3389/fonc.2025.1642866 (PMC12433850; doi:10.3389/fonc.2025.1642866)

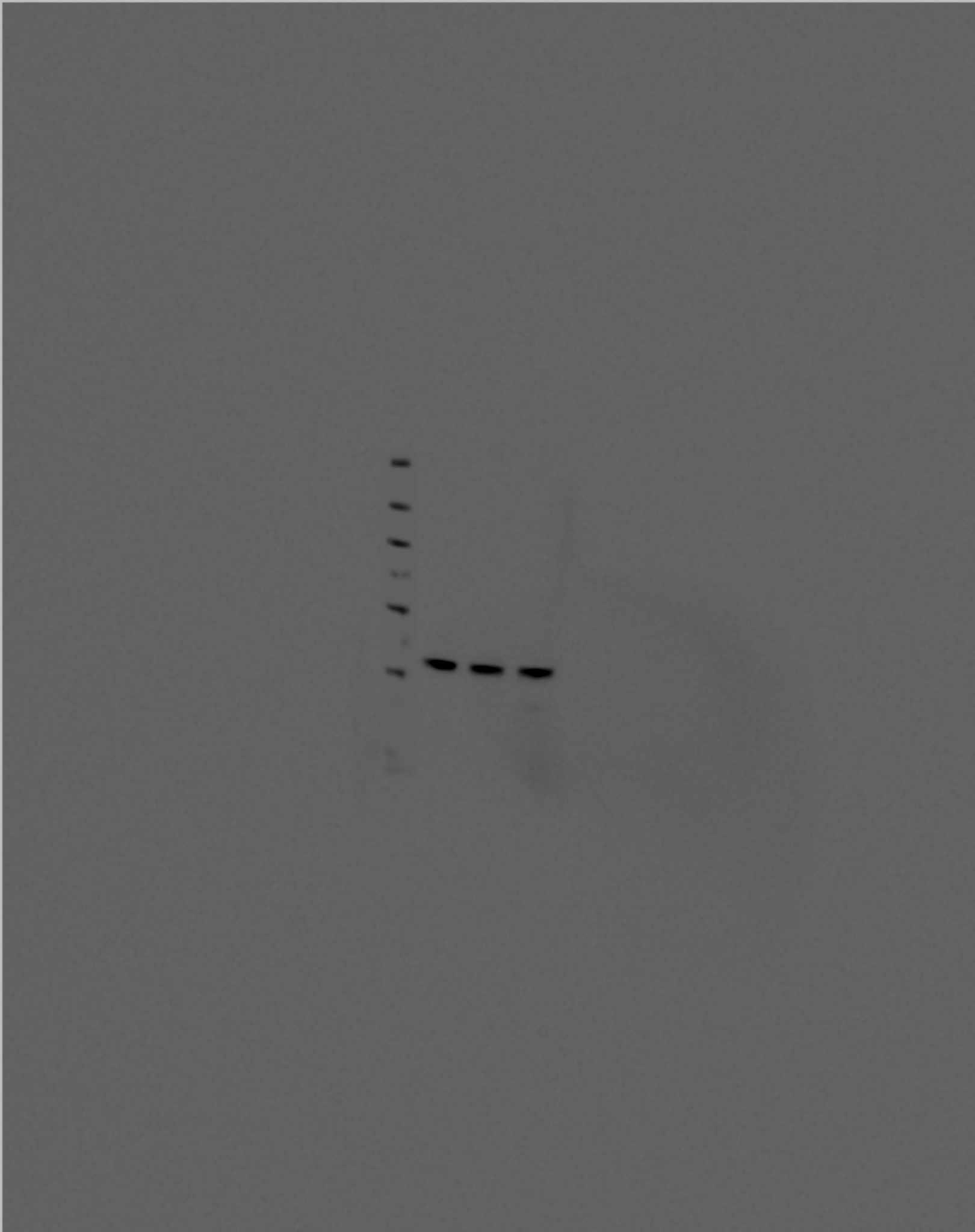

Supplement: Supplementary file 5 [file Image1.jpeg]

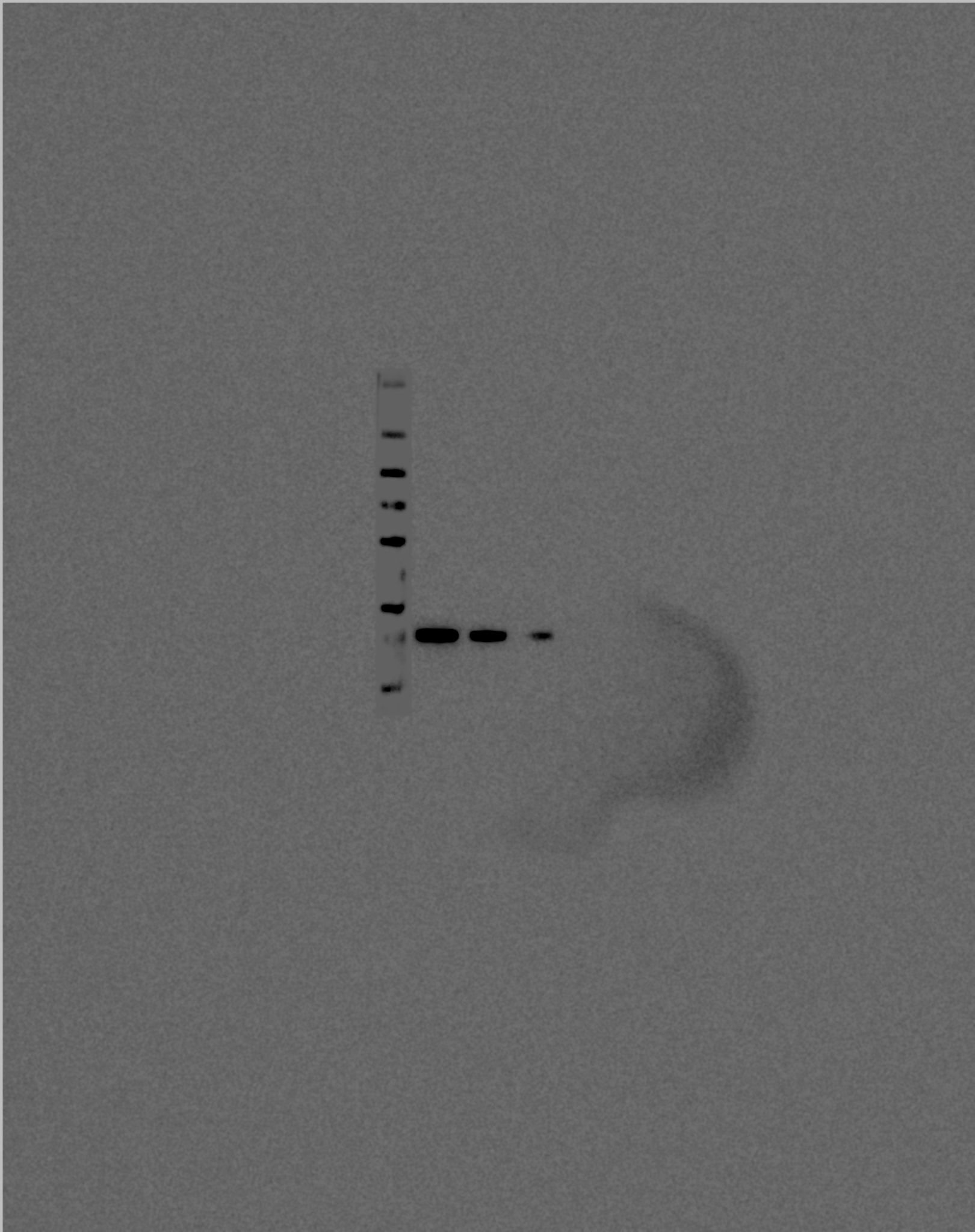

Supplement: Supplementary file 6 [file Image2.jpeg]
